# Assessing signals of selection and historical demography to develop conservation strategies in the Chilean emblematic *Araucaria araucana*

**DOI:** 10.1038/s41598-021-98662-w

**Published:** 2021-10-15

**Authors:** Glenda Fuentes, Fidelina González, Javier Saavedra, Patricio López-Sepúlveda, Pedro F. Victoriano, Tod F. Stuessy, Eduardo Ruiz-Ponce

**Affiliations:** 1 Departamento de Botánica, Facultad de Ciencias Naturales y Oceanográficas, Universidad de Concepción, Concepción, Chile; 2 Departamento de Biología Celular, Facultad de Ciencias Biológicas, Universidad de Concepción, Concepción, Chile; 3 Departamento de Agronomia, Universidade Estadual de Maringá, Av. Colombo 5790, Maringá, PR Brasil; 4 Departamento de Zoología, Facultad de Ciencias Naturales y Oceanográficas, Universidad de Concepción, Concepción, Chile; 5 Herbarium and Department of Evolution, Ecology, and Organismal Biology, The Ohio State University, Columbus, OH 43210 USA; 6 Department of Botany and Biodiversity Research, University of Vienna, 1030 Vienna, Austria

**Keywords:** Plant sciences, Environmental sciences

## Abstract

Loss of genetic diversity reduces the ability of species to evolve and respond to environmental change. *Araucaria araucana* is an emblematic conifer species from southern South America, with important ethnic value for the Mapuche people (Pehuenche); the Chilean Government has catalogued its conservation status as vulnerable. Climatic fluctuations were potentially a major impact in the genetic variation within many tree species. In this context, the restricted geographic distribution of *A. araucana* in Chile appears to be a consequence of the Last Glacial Maximum (LGM). During the past two centuries, strong human intervention has also affected the geographical distribution and population sizes of *A. araucana*. Reduction of population size may cause loss of genetic diversity, which could affect frequency of adaptive loci. The aims of this study were to know the existence of potential loci under selection and populations with genetic, demographic disequilibrium in the Chilean distribution of *A. araucana*. Based on 268 polymorphic AFLP loci, we have investigated potential loci under selection and genetic, demographic disequilibrium within seven Chilean populations of *Araucaria araucana*. Correlation of 41 outlier loci with the environmental variables of precipitation and temperature reveals signatures of selection, whereas 227 neutral loci provide estimates of demographic equilibrium and genetic population structure. Three populations are recommended as priorities for conservation.

## Introduction

Genetic structure within and among populations is modeled by the interaction of genetic drift, gene flow, mutation, and natural selection^1^. Molecular data have helped to identify the effects that natural history traits, phylogeographic history, and environmental factors have had on the population genetic structure of plants^1–4^. Climatic parameters are key to determining the distribution of plant species^5^, especially in the current context of climate change^4,6,7^.

In fact, the present geographical distribution and genetic variation within and among populations of tree species may have been a consequence of climate fluctuations during the Quaternary^8^, resulting in historical demographic events such as population contractions or expansions^9^.

The Last Glacial Maximum (LGM) modified the geographical distribution of many plant species because of ice advancing on both hemispheres^10^. Using paleobotanical information and geomorphic evidence, the existence of plant refugia in Europe as well as in North and South America during the Quaternary has been proposed^10,11^. These refugia would have been areas that remained as patches free of ice^12^, and recolonization of glaciated areas would have occurred from refugia after glacial retreat^10,13^, as demonstrated in conifers in the Northern Hemisphere during Quaternary LGM^14^. Inferences from the fossil record during the LGM in Chile, in the high elevation forests of the Coastal and Andean ranges, suggest discontinuous plant distributions for species such as *Fitzroya cupressoides*, *Austrocedrus chilensis* and *Araucaria araucana*^15^. For this latter species, the LGM would likely have modified not only its geographical distribution but also number and sizes of populations^16,17^, which might be reflected in current patterns of genetic variation^18–22^. Three groups of structured Chilean populations of *Araucaria araucana*, based on isozymes, have been shown to be congruent with the glacial refugia hypothesis^19^. Fragmented populations of the species in Argentina, based on five haplotypes of non-coding chloroplast DNA, show low genetic differentiation and structure^20–22^.

Genetic diversity studies with different markers have allowed estimation of genetic diversity in *A. araucana* from both neutral and adaptive loci. Neutral loci, e.g. from RAPDs, cannot detect adaptive genetic divergence across the Andean Ranges. Adaptive variability can be estimated using outlier loci that show signals of selection^23–25^, such as in relation to drought tolerance, which would be important for conservation^18^. Potential loci under selection (outlier loci) are portions of the genome that show different patterns of variation from those of neutral loci and can be identified through statistical methods^26–31^. Statistical correlation between the presence of outlier loci and environmental parameters allows assessment of environmental factors driving selection^26,32–36^. Therefore, it is possible not only to identify potential loci under selection but also to correlate these loci with environmental parameters, which vary greatly within the geographical distribution (Andean and Coastal) of *Araucaria araucana*.

**Figure 1:**
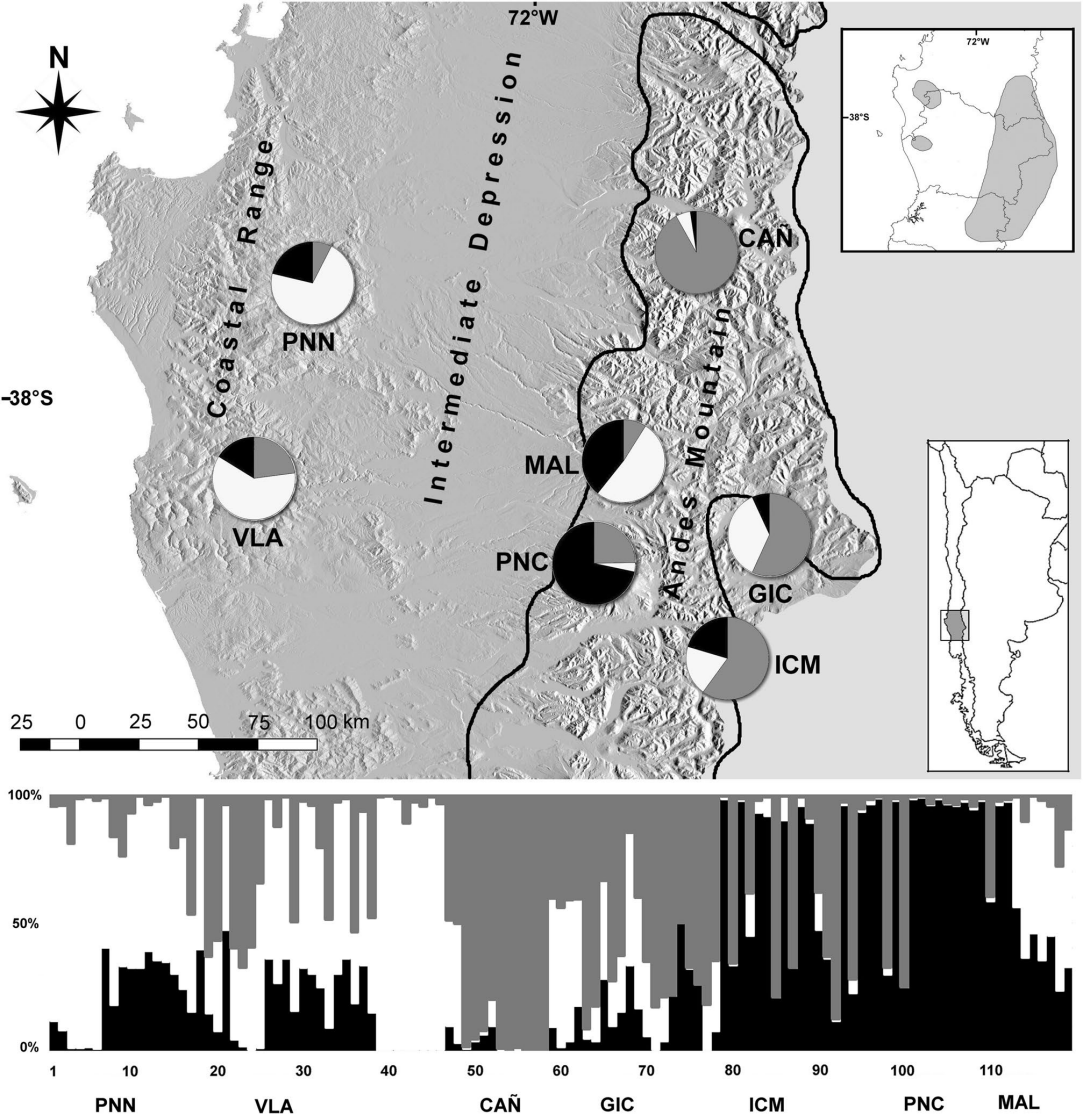
Distribution of collection sites of *Araucaria araucana*. Circles indicate the proportions of haplotypes in each site. Dark line indicates limit of the ice sheet during LGM^89^. Right-upper box shows the *Araucaria araucana* distribution^90^. Right-lower box shows the Chilean geographical context of studied sites. Below-barplot indicates the individual genetic composition of *K* = 3 genetic group. Map was made using free and Open Source Software licensed under the GPL inkscape v. 1 (https://inkscape.org/).

*Araucaria araucana* displays a disjointed geographical distribution (Coastal-Andean), being found in the Andes between 37°24′S and 40°03′S, and in the Coastal range only in two small locations, between 37°30′S and 38°S and about 38°30′. The Longitudinal Chilean Valley (Intermediate Depression) separates the Coastal and Andean ranges (Fig. 1). Differences in environment in which *A. araucana* grows mainly involve temperature, soil type, duration of snow cover, and precipitation^37,43,44^.

As a result of unsustainable management during the past two centuries^37–39^, Chilean *A. araucana* populations have suffered a drastic reduction in size^40^. For this reason, the Chilean government now prohibits the cutting of this species^41^ and has catalogued it as vulnerable, according to the International Union for Conservation of Nature and Natural Resources^42^ and the Chilean government Supreme Decree Law DS 51 of 2008 (Ministerio de Medio Ambiente de Chile. 2008. Decreto Supremo N° 51/2008. Diario Oficial de Chile. Junio 30, 2008: 4).

Multiple glacial events have historically impacted habitats of *Araucaria araucana*. In the Southern Hemisphere, it has been proposed that the LGM took place ca. 20.000 years bp, termed Llanquihue III^45^. This last glacial period not only caused important climatic changes, such as decrease in maximum temperature and rainfall patterns, but it also would have affected plant distributions^15^. *Araucaria araucana* populations appear to have been disturbed in the LGM (including tardiglacial and postglacial: 14,000 to 6000 years bp), not only in their geographical distribution^46^, but also in their genetic patterns^18–22^. During this period, important climatic variations occurred that influenced the distribution of flora in the South-Central areas of Chile^46,47^. However, based on geomorphological and palynological evidence, some places in both the Coastal and Andean regions have been proposed as glacial refugia^48^. The consequences of climate change during the Quaternary have been evaluated in several species, and the existence of multiple refugia in the temperate forests of Southern Chile and marginal zones with Argentina has been hypothesized. An example is *Fitzroya cupressoides* (Cupressaceae)*,* which has been studied using isozyme^49^ and RAPD markers^11^. These studies concluded that multiple refugia existed during the LGM north of Patagonia. In *Araucaria araucana* the existence of glacial refugia has also been proposed because of evidence gathered from RAPD markers^18^ and allozyme studies^19^.

Taking into account the available information on genetic variation in *A. araucana*^18–22,50^, and considering environmental and geographical differences in the distributions where this species grows, different genetic diversity patterns might be expected between populations in Coastal and Andean ranges. This information is important for conservation strategies^24^. In this context, we hypothesize that distributions of outlier loci in *Araucaria araucana*, might be correlated with environmental factors, such as temperature and humidity, which are known to have high impact on distributions of plant species^4,5,35^. On the other hand, because of climatic changes caused by glaciation, it might be expected to find populations in recolonized areas due to expansion from populations acting as refugia^51,52^. This is an important aspect to consider in these populations because they might have been more susceptible to loss of alleles^53–56^, including outlier loci with adaptative significance. Based on this background information, amplified fragment length polymorphism (AFLP) markers were employed for the following objectives: (1) to detect potential loci under selection, (2) to correlate loci under selection with climatic parameters, and (3) to assess if existing populations of *A. araucana* are in genetic demographic disequilibrium.

## Results

### Signals of natural selection through detection of outlier loci and their correlation with environmental variables

Each fragment represents an AFLP locus. Selective amplifications yielded 268 polymorphic fragments across 119 samples (100% polymorphic); each fragment represents an AFLP locus (binary data matrix with polymorphic loci is available in Supplementary Table S1 online). Forty-one outlier loci were found, assuming a 95% and 99% confidence level (*p* < 0.05 and *p* < 0.01). A list of outlier loci is available in Supplementary Table S2 online. Sixteen outlier loci were distributed at higher *F*_*st*_ values than expected under neutrality (two loci highly significant, *p* < 0.01, loci 53 and 109, in the Coastal range), 25 loci were distributed at lower *F*_*st*_ values than expected under neutrality (4 loci highly significant, *p* < 0.01, loci 180, 181, 194 and 254, both in the Coastal and Andean range). Correlation among *F*_*st*_ and *He* values of loci is available in Supplementary Fig. S1 online. Five outlier loci were found only in the Andes Mountains (loci 7, 23, 67, 123 and 142) and four loci were found only in the Coastal range (loci 53, 63, 109 and 138). Thirty-two outlier loci were found in both geographical ranges (Andes and Coastal; see Supplementary Table S2 online). Three environmental variables were selected (Table 1) from nineteen environmental variables available from Worldclime http://www.cru.uea.ac.uk/ and http://badc.nerc.ac.uk/data/cru/)^57^. The selected variables were correlated with outlier loci. From the 41 outlier loci, 6 of them (loci 26, 53, 65, 185, 239, and 259) showed significant correlation with precipitation (BIO18, Table 2) and temperature variables (BIO2, Table 2). Locus 65 was correlated with precipitation of the coldest quarter (BIO19). Locus 53 was correlated with the temperature variable (BIO2) and is present only in the Coastal distribution range. Locus 259, which was present in both distribution ranges, was highly correlated with variable BIO2 (Table 2).

**Table 1:** Selected environmental variables from PCA groups. Abb: Abbreviation from Worldclime^57^.

Description	Abb
Mean Diurnal Range Temperature (Mean of monthly (max- min))	BIO2
Precipitation of Warmest Quarter (mm)	BIO18
Precipitation of Coldest Quarter (mm)	BIO19

**Table 2:** Distribution of highly significant outlier loci in collected sites, correlated with environmental variables (Confidence level, gray = 95%; black = 99%). 0 = Locus absent; 1 = Locus present

Outlier loci	BIO2	BIO18	BIO19	PNN	VLA	CAÑ	GIC	ICM	PNC	MAL
Locus 26	gray			1	1	0	0	1	1	1
Locus 53	gray			1	1	0	0	0	0	0
Locus 65		gray	gray	1	1	1	1	1	1	1
Locus 185		gray		1	1	1	1	1	1	1
Locus 239		gray		1	1	1	1	1	1	1
Locus 259	black			1	1	1	1	1	1	1

### Estimation of spatial distribution of genetic variation (genetic groups)

The reduced data matrix with 227 loci, by excluding outlier loci, was employed for estimating genetic groups and population structure (AMOVA). From the resulting data it was determined that three is the most probable number of genetically homogeneous groups (*K* = 3, Ln’|k| 2407.355; Table 3; see Supplementary Fig. S4 online). According to the barplot in Fig. 1, group 1 includes only PNC (Conguillio National Park), Group 2 consists of PNN (Nahuelbuta National Park), VLA (Villa Las Araucarias), and MAL (Malalcahuello), and group 3 included CAÑ (Cañicú), GIC (Galletué-Icalma) and ICM (Icalma-Melipeuco). The Heidelberg and Welch diagnostic test confirmed the stability and null autocorrelation of Gibbs chains for each *F*_*st*_ value (*p* = 0.05), validating the model used for structure analyses. Figure 1 shows the resulting barplot of genetic groups and the geographical distribution of haplotype proportions among studied sites.

**Table 3:** Populations belonging to each genetic group. Values of mean, median and mode, Bayesian credibility intervals (Confidence level, 95% of probability) of the *F*_*st*_ values for each genetic group (*K* = 3).

Genetic group	Population	Mean	Median	Mode	IC 95%	
					Lower	Upper
Group 1	PNC	0.527	0.527	0.5273	0.483	0.572
Group 2	PNN, VLA, MAL	0.108	0.108	0.1093	0.082	0.135
Group 3	CAÑ, GIC, ICM	0.4162	0.416	0.4148	0.379	0.454

Results from AMOVA show that the highest level of genetic diversity resides within sites (74.79%), with *F*st = 0.252 (*p* = 0.001; Table 4). The analysis of the Andean Mountain localities, as a group, gave a higher population structure than those of the Coastal range (Table 4).

**Table 4:** Distribution of genetic diversity obtained from AMOVA.

Source of variation	Degree of freedom	Percentage of variation
**Species**		
Among groups	2	9.75
Among sites within each group	4	15.46
Within sites	114	74.79
*F*_*sc*_ 0.171 *p* value = 0.000 *F*_*st*_ 0.252 *p* value = 0.000 *F*_*ct*_ 0.097 *p* value = 0.053		
**Coastal**		
Among sites	1	3.94
Within sites	45	96.06
*F*_*st*_ 0.039 *p* value = 0.008		
**Andes**		
Among sites	5	28.24
Within sites	65	71.76
*F*_*st*_ 0.282 *p* value = 0.000		

### Demographics

Mismatch analysis (excluding the outlier loci) shows that the distribution of frequencies of the number of differences between pairs of haplotypes was unimodal in two localities VLA and PNC and multimodal distribution was found in the remaining localities (Supplementary Fig. S2 online). The results of Fu´s neutrality *Fs* test^58^ proved to be consistent with those of mismatch distribution analysis, showing *P* values near to 0.02, for demographic expansion in the two sites, which displayed unimodal distributions (Table 5).

**Table 5:** Demographic parameters: *r* (generation number after expansion), *Ѳ*_*0*_ (population size before expansion), *Ѳ*_*1*_ (population size after expansion), *Fs* (Fu’s neutrality test).

Locality	*r*	*Ѳ*_*0*_	*Ѳ*_*1*_	*Fs*	*p* value
PNN	40.00	302.397	4423.972	−0.594	0.323
VLA	40.00	302.397	4423.972	−4.561	0.05
CAÑ	16.0	71.999	3662.382	−0.587	0.236
GIC	40.00	302.397	4423.972	1.844	0.561
ICM	40.00	302.397	4423.972	0.036	0.354
PNC	40.00	57.599	7262.381	−6.946	0.020
MAL	56.00	14.399	3662.382	1.172	0.426

## Discussion

The current distribution of *Araucaria araucana* could be a remnant of a more extensive past distribution^46,47^. This past distribution would have been severely reduced, not only due to glacial and post-glacial events during the last 20,000 years, but also due to other factors such as volcanism and human influence (fires, exploitation of forest products, and soil management practices)^18,37^. Lara et al.^59^, established that only 52% of the native *Araucaria araucana* forest that existed prior to the colonization by Europeans is still present, accounting for 261,073 hectares, 47% of which are currently protected by the Chilean Government^60^. In this context, important results have been obtained from several studies that have estimated genetic distribution patterns for conservation purposes^18–22,50^**.** However, two aspects are important to consider in population/species conservation: loss of haplotypes of loci under selection^61^, and current genetic demographic equilibrium^62^.

The results from the present work show the existence of loci under selection that are correlated with climatic variables of temperature and precipitation. On the other hand, some localities are revealed to be in genetic demographic disequilibrium. These two aspects have high relevance for making decisions on the establishment of protected areas for this species.

Loci under selection can be defined as those portions of the genome that are under selective pressures^23^ and that allow species to adapt to environmental changes^26^. Habitat reduction and population size reduction are important factors in the loss of diversity, with loci under selection, generally being haplotypes or alleles in low frequency, within a population^24^. Identification of loci under selection within a population, therefore, becomes an important criterion for development of conservation strategies^63^. Considering that environmental parameters are key in determining plant distributions^5,64^, the correlation between genetic and environmental parameters is also relevant for conservation. In this context, 32 outlier loci were shared in both geographic distributional ranges of *Araucaria araucana*. Five outlier loci (loci 7, 23, 67, 123 and 142) were found only in the Andean range and four (loci 53, 63,109 and 138) were found in the Coastal range. For *Araucaria araucana*, locus 53 was correlated with the temperature variable (BIO2). Temperature has been recognized as an important factor in selection, responsible for population differentiation on altitudinal and latitudinal clines^4^. Within the Andean distribution, temperatures range from −5 °C to −10 °C in the winter to 30 °C in the summer. On Nahuelbuta Mountain (PNN), the range of temperature variation is lower than that in the Andes Mountains, from −1 °C in the winter to 9 °C in the summer [43; pg. 376].

On the other hand, loci 65, 185 and 239 are correlated with precipitation variable (BIO18), possibly related to two sources of differentiation. The first is variation in precipitation registered between the Andean and Coastal ranges. For the Andes an annual rainfall between 1000 and 4500 mm is observed, depending on altitude. On Nahuelbuta Mountain, rainfall ranges from 1300 to 3000 mm annually^43^. The second source of differentiation relates to marked differences in precipitation within the Andes. On the westernmost areas, precipitation varies between 2000 to 4500 mm, however, on higher altitude (Eastern areas) near the limit with Argentina, the annual rainfall average is 1000 to 1900 mm (e.g., in Lonquimay)^65,66^, and snow is more prevalent than rain. Differences in environmental variables found in the coastal range are less pronounced than in the Andean range, potentially favoring the selection of alleles correlated with such environmental traits as has been hypothesized in the mountain rainy neotropical forest^17^. In the Andes, local adaptation to specific different climatic conditions favors a higher degree of structure among the different sites within this range (as shown in Table 4).

Outlier loci could be important for adaptation to local environmental conditions, which are highly variable between geographical ranges. Our results showed five outlier loci (loci 7, 23, 67, 123 and 142) distributed only in the Andean range (Supplementary Table S2 online), and five correlated with precipitation and temperature (Table 2). These findings could be very important in view of global climate change, especially as relating to factors of temperature and precipitation, which could seriously affect physiology of the plants^67^. Correlation of outlier loci or adaptive loci with temperature has been demonstrated in conifer species under conditions of global climate change^68^.

The detection of loci correlated with different environmental conditions, usually found in low frequencies, is important for confronting rapid changes in climatic conditions^4^. Because *Araucaria araucana* has been catalogued as a vulnerable species, it is important to maintain population sizes to help preserve loci that may confer adaptive advantage. In the case of *Fagus sylvatica*, it has been reported that an evolutionary response in a short period of time will depend upon the current available variation pool present within the species distributional range^35^. Based on the relationship between current genetic structure in *A. araucana* and glacial and post-glacial events, three genetic groups were found: Group 1 consists of only one Andean population (PNC), Group 2 includes localities from both Andean and Coastal ranges (PNN, VLA, MAL), and Group 3 contains only Andean populations (CAÑ, GIC, ICM). These results are consistent with the existence of multiple glacial refuges within the Andean Range. This hypothesis is in accordance with those of other studies on different animal and plant species, in which the existence of multiple refugia in the Andes has been postulated^11,19,69–71^. In addition, two of the evaluated localities are in demographic disequilibrium (expansion), and one of them comprises a unique genetic group (PNC). The grouping of some Andean and Coastal populations of *A. araucana* in the same genetic set, based on neutral genes, seems not very logical. However, the above could be explained by referring to probable distributional dynamics associated with glacial periods, which historically would have allowed gene flow between populations of both mountain ranges. During glacial periods, highland vegetation would have colonized the lower areas of the Intermediate Depression, bringing together populations from the Andes and the Coast. For example, according to this, for certain lineages of the lizard *Liolaemus pictus*, closely associated with *Nothofagus* and *Araucaria* forests, there is phylogeographic evidence of the absence of reciprocal monophyly between currently disjunct populations in both mountain ranges, which suggests that historically they would have experienced Andes-Costa gene exchange^72^. The results of selection signals, and the distribution of neutral genetic variation within and among Coastal and Andean populations (Group 2), strengthen the need for conservation strategies within the species.

The distribution of frequencies of differences between haplotype pairs (mismatch) for two sampled sites, Araucaria Village and Conguillio National Park, was unimodal (Supplementary Fig. S2 online). Likewise, Fu test^58^ values for these sites are the lowest and probably in demographic disequilibrium (Table 5). Furthermore, the Northernmost distribution (Cañicú) contains the lowest genetic diversity (a genetic bottleneck; data not presented here), in conformity with results from other authors^18,19,21,22^.

Based on these results, there are three important geographical areas of *Araucaria araucana* to be considered. First, Araucaria Village (VLA), which was declared “Patrimonio Nacional” by the Chilean government in 2020 (http://patrimonio.bienes.cl/patrimonio/villa-las-araucarias/), and their individuals have been declared an endangered species by the Government of Chile. This population has experienced a demographic expansion (demographic disequilibrium) that affected the allele frequence patterns. This site is the lowest altitude within the range of distribution of the species, which could have important adaptive consequences due to outlier loci present only in the Coastal range (Nahuelbuta Mountain and Villa Las Araucarias). This would be important in terms of global climate change, as well as conservation priorities, because this site could be regarded as a “genetic sink” for adaptation within the range of the species. Furthermore, high levels of genetic diversity have been documented for this area, compared to other sites^18,19^. Araucaria Village is in a non-protected area and hence completely exposed to human intervention, which could lead to serious negative factors for reproductive dynamics of this species, resulting in loss of loci with adaptive value.

Second, Cañicú (CAÑ) is another important geographical area, the northernmost distribution for this species, and which presents the lowest genetic diversity indices^18,19^. This site exhibits an important difference between the effective population size before and after the beginning of the expansion (*Ѳ*_*0*_–*Ѳ*_*1*_), suggesting the occurrence of a genetic bottleneck, as reported by Martin et al.^22^, which has been corroborated by previously documented low levels of genetic variation^18,19^. Such genetic particularity is relevant, considering that outlier loci (probably under selection) were documented for this locality in the present research.

Third, another important area is Conguillio National Park (PNC), due to genetic differences found in this area that constitute a different genetic group also in demographic disequilibrium. Although some of this area is under protection (Conguillio National Park), it may be necessary for conservation purposes to increase the size of the reserve in this zone of *Araucaria* Forest because of demographic characteristics that make this area susceptible to climate change^73^.

Regarding the relationship between distribution of outlier loci in different sampling sites, we found that the clearest pattern separates the Coastal range from the Andean range. There are 4 loci that are distributed only in the coastal sites and five only in the Andes, which could reflect local adaptations to different temperatures. Another interesting pattern is that most of outlier loci are present only on the western slope of the Andean range, which are postulated as recolonized areas from Andean refugia^22^ (see Distribution of outlier loci in Suppl. Material Fig. S6). Furthermore, the distribution of the values of climatic variables correlated with outlier loci does not show concordance with the genetic groupings. It can also be observed that for the loci correlated with the temperature variable (BIO2), locus 53 has the highest allelic frequencies in the Coastal population VLA and disappears in the Andes range distribution (see Supplementary Table S2, online). For the variable precipitation (BIO18), a frequency value of 1 is recorded for locus 185 in the locality of Malalcahuello and for locus 239 and locus 65 values over 0.5 are recorded in the localities of Conguillio and Malalcahuello (Table 2) (see Supplementary Fig. S8 online).

In summary, based on the results of this study, we suggest the following localities should be included as priority protected areas: Villa Las Araucarias and Cañicú-Ralco. It would also be highly desirable to expand geographic sampling in future studies, especially on the western slope areas that have been recolonized from glacial refugia in the Andes range, and which have shown different frequencies of outlier loci in response to temperature and precipitation.

## Materials and methods

### Ethics statement

The sampling in this study is in compliance with relevant institutional, national, and international guidelines and legislation. This study was conducted in accordance with all Chilean Republic laws. For our research activities, we collected under a permit authorized by CONAF (Corporación Nacional Forestal) for allowing removal of leaf samples from *Araucaria* trees inside protected areas^19^.

### Sampling

Based on previous results of genetic structure in *Araucaria araucana*^22^ and considering that this species is catalogued as vulnerable^42^, only some of the existing localities were considered in this study. The localities were selected based on the following criteria: first, both Coastal and Andean distributional ranges are highly structured, with different patterns of genetic diversity^22^. Due to very different environmental conditions between both distributional ranges, adaptative variation could be postulated between Coastal and Andean populations^24^, and the Coastal range has been postulated as having harbored glacial refugia^22,48^.

Second, in the Coastal range, the unique two places where *A. araucana* grows were selected, Villa Las Araucarias (unprotected area) and Nahuelbuta Mountain, specifically Nahuelbuta National Park (a protected area). Different patterns of genetic diversity have been postulated for these sites. Villa las Araucaria is located in the southernmost border of the coastal distributional range and Nahuelbuta Mountain is in the center of the Coastal distribution of this species^45^. Villa Las Araucarias showed lower genetic diversity and higher genetic structure than Nahuelbuta Mountain^22,74^.

Third, some areas of distribution in the Andes were free of ice during the LGM, specifically, the northernmost Andean distribution and Galletué-Icalma Valley^48^ and have been postulated as glacial refugia for some tree species (e.g., *Fitzroya cupressoides*^11^). Westernmost Andean localities as Conguillio National Park and Malalcahuello could have been recolonized from these refugial areas^22^. Different patterns of genetic diversity have been documented between glacial refugia and recolonized areas with the former exhibiting higher genetic diversity than the latter^10,75^. Information about outlier loci from different sites with different genetic patterns is listed in Table 6.

**Table 6:** Abbreviations (Abb) and locations of the collection sites, altitude (meters above sea level), geographic coordinates and total number of individuals (N).

Abb	Location/characteristics	Altitude	Coordinates	N
PNN	Parque Nacional Nahuelbuta/ Protected, centre of Coastal distribution, glacial refuge	1300	37°78′30″S–72°998′22″W	15
VLA	Villa Las Araucarias/unprotected, southernmost Coastal distribution, glacial refuge	1000	38°48′97″S–73°24′54″W	32
CAÑ	Cañicú/unprotected, Northernmost Andean distribution, glacial refuge	1000	37°82′82″S–71°59′05″W	11
GIC	Among Galletué-Icalma/Unprotected, Western Andean distribution, glacial refuge	1300	38°04′07″S–71°27′55″W	6
ICM	Among Icalma -Melipeuco/ Unprotected, Western Andean distribution, glacial refuge	1300	38°70′35″S–71°33′11″W	12
PNC	Parque Nacional Conguillío/ Protected, Estern Andean distribution, recolonized	1100	38°66′66″S–71°65′00″W	36
MAL	Among Malalcahuello-Lonquimay/ Unprotected, Eastern Andean distribution, recolonized	1040	38°47′22″S–71°57′18″W	7

### DNA extraction

Young leaves of *A. araucana* were obtained from 119 trees from seven sites in the Chilean distributional range, five sites within the Andean distributional range, and two sites within the Coastal range (Table 6; Fig. 1). The number of trees included in this analysis per sampling site varied from 7 to 45, we chose trees separated by at least 7 m to prevent sampling of clones from the same individual. The analyses were carried out at the species, genetic group, and mountain range level without considering the origin of the individuals sampled by geographical location, but rather the totality of individuals in the sample. Twenty-five individuals are the minimum recommended for the type of marker used in this work^76^. In the Coastal range, one site is protected by the Chilean government (Nahuelbuta National Park) and another is an unprotected site (Villa Las Araucarias). In the Andean range one site included a protected site (Conguillio National Park) and four are unprotected sites, two of them having been proposed as ice-free sites during the LGM (Galletué-Icalma and Icalma-Melipeuco)^48,77^ and supported by genetic evidence^19^.

Total DNA was extracted following the CTAB method^78^ with some modifications^79^. Before extraction, pulverized leaves obtained by grinding the tissue with liquid nitrogen were suspended in HEPES buffer and centrifuged at 10,000 rpm for 5 min. Extracted DNA was treated with RNase at 37 °C for 30 min. The quality of the extracted DNA was assessed by running an aliquot on a 1% agarose gel. DNA concentration was quantified by UV spectrophotometry (UV 160, Shimadzu) and samples were stored at −20 °C until needed.

### AFLP amplification

AFLP was performed according to protocol described by^70,80^, which consists of three steps. First, digestion-ligation of 0.5 µg genomic DNA was carried out using *EcoRI* and *MseI* at 37 °C for 2 h. Adaptors were ligated to the ends of each digested DNA fragment (*EcoRI* and *MseI* adaptors). Second, a preselective PCR was performed using primers complementary to the adaptors plus one selective base (*EcoRI* + A, and *MseI* + C). Third, the final (selective) PCR with primers containing three selective bases (*EcoRI* + 3 and *MseI* + 3) was performed using the preselective PCR product as template (diluted 1:10 in 1xTE buffer). Sixteen combinations of selective primers were tested, from which EcoRI-ACT/MseI-CAG and EcoRI-ACC/MseI-CTG were selected because they resulted in the clearest and most reproducible amplification patterns. Both amplification reactions were performed in an Eppendorf Master Cycler Gradient thermalcycler. PCR fragments from the selective step were sized on an Applied Biosystems Prism 310 sequencer with an internal standard and analyzed on GeneScan 2.1 (Applied Biosystems). Results were then imported to Genographer (version 1.1.0, Montana State University 1998; http://hordeum.msu.montana.edu/genographer/; https://sourceforge.net/projects/genographer/). Each AFLP fragment was coded as either present = 1 or absent = 0 in all samples, yielding a binary data matrix. All AFLP experiments were done in the Department of Botany and Biodiversity Research, University of Vienna, Vienna, Austria.

### Data analysis

#### Signals of natural selection through detection of outlier loci and their correlation with environmental variables

Identification of potential loci under selection and their frequencies was performed using Arlequin 3.5.2^81^. Outlier loci with higher or lower *F*_*ST*_ values than under neutral expectations, and which fell outside 95% confidence levels, were assumed to be under directional or balancing selection.

The environmental variables were obtained from Worldclim^57^. To select environmental variables with less correlation with each other, a Principal Component Analysis (PCA) was carried out. The PCA and the standardization of the data were performed in R, versión 4.0.2^82^. Using the “prcomp” function. The variables selected from the PCA analysis (BIO19, BIO28, BIO2) were correlated with the allele frequencies in each of the loci under selection and tests with Pearson’s or Spearman’s correlation coefficient depending on whether the data were or not normally distributed. Analysis was performed in R, version 4.0.2 using the Rcomdr package^82^ (see Supplementary Table S3 online).

#### Determination of spatial distribution of genetic groups from neutral loci

Information on neutral loci, especially how they are distributed, is also a contribution to understanding the conservation status of a plant species^24^. To determine genetic groups, only neutral loci were used, as recommended by STRUCTURE software, to avoid underestimation or overestimation of genetic parameters (see Supplementary Fig. S3 online).

After removal of outlier loci from the original data matrix, analyses of genetic structure were made with STRUCTURE 2.3.3^83^. Twenty iterations per run using the no admixture model, assuming several gene pools (*K*) between 2 and 8, were done (see Supplementary Figure S5 online). Simulations included 50,000 Markov Chain Monte Carlo (MCMC) steps following a period of 5000 burn-in and sampling each 100 iterations, to avoid autocorrelation of chains. *K* was estimated^84^. The simulations for the selected *K* were repeated, using 1 × 10^6^ MCMC steps with 200,000 iterations as burn-in. The convergence diagnosis and output analysis (CODA) package for R-project was then used to test the null autocorrelation of Gibbs chains^85^. Bayesian credible intervals (95%) for *Fst* values were calculated.

#### Estimation of demographic parameters

Demographic parameters were estimated from a Mismatch Distribution analysis using the program Arlequin 3.5^81^. These included: (1) the number of generations since expansion (*τ*), (2) pre-expansion population size (*θ*_*0*_), and (3) post-expansion population size (θ_1_)^86^. Additionally, selective neutrality using Fu’s *Fs* statistic^58^, which is sensitive to signals of population expansion, was tested.

Storz and Beaumont^9^ state that the current genetic structure and variability of many species has been defined by historical demographic events such as population retractions or expansions, deriving from climatic changes or bottlenecks. From studies of genetic variation in natural populations, tools have been developed, such as the analysis of distributional frequencies of paired differences of haplotypes (Mismatch Distribution)^87^, which allow detection of historical population variations that may have been a consequence of events such as glaciations^88^. This analysis allows the construction of a histogram, where multimodal-type distributions represent populations that have not undergone changes in their population sizes, which has been considered demographic equilibrium. Unimodal-type distributions are typical of populations that have recently experienced variations in their size or genetic demographic disequilibrium^87^.

## Supplementary Information

Supplementary Information.
