# Supplementary material for: Assessing signals of selection and historical demography to develop conservation strategies in the Chilean emblematic Araucaria araucana
Source: Sci Rep. 2021 Oct 15;11:20504. doi: 10.1038/s41598-021-98662-w (PMC8521589; doi:10.1038/s41598-021-98662-w)

Supplementary Information

Assessing signals of selection and historical demography to develop conservation strategies in the Chilean emblematic *Araucaria araucana*

Glenda Fuentes

Fidelina González

Javier Saavedra

Patricio López- Sepúlveda

Pedro Victoriano

Tod F. Stuessy

Eduardo Ruiz-Ponce

Supplementary Table S1. Binary data matrix of polymorphic AFLPs loci.

Site	Ind.	Locus																			
		1	2	3	4	5	6	7	8	9	10	11	12	13	14	15	16	17	18	19	20
	1	0	1	1	1	1	1	0	0	1	1	0	1	0	0	1	1	1	1	1	0
	2	1	0	0	1	1	1	0	0	0	1	0	0	0	1	1	1	1	0	1	0
	3	1	1	0	1	1	0	0	0	0	1	0	1	0	1	1	1	1	0	0	0
	4	0	1	1	1	1	1	0	0	0	1	0	1	0	1	1	1	1	0	0	0
	5	1	1	0	1	1	1	0	0	0	1	0	1	0	1	0	1	1	0	0	0
	6	1	1	1	1	1	1	0	0	0	1	0	1	0	1	1	1	1	0	0	0
	7	1	1	1	1	0	1	0	0	1	1	0	1	0	1	1	1	1	1	1	0
PNN	8	1	1	0	0	0	1	0	0	1	1	0	1	0	1	1	1	1	1	1	0
	9	1	0	1	1	0	0	0	0	1	1	0	0	0	1	1	1	1	0	1	0
	10	1	1	1	1	1	1	0	0	1	1	0	1	0	1	1	1	1	0	1	0
	11	1	1	1	1	1	1	0	0	1	1	0	1	0	1	1	1	1	0	0	0
	12	1	1	1	1	1	1	0	0	1	1	0	1	0	1	1	1	0	0	0	0
	13	1	0	1	0	1	0	0	0	1	1	0	0	0	1	1	1	0	0	1	0
	14	1	1	1	1	1	1	0	0	1	1	0	1	0	1	1	1	1	0	1	0
	15	1	1	1	0	1	0	0	0	1	1	0	1	0	1	1	1	1	0	0	0
	1	1	1	1	1	1	1	0	0	1	1	0	0	0	0	0	0	0	0	0	0
	2	1	1	0	1	0	1	0	0	1	1	0	0	0	0	0	1	1	0	1	0
	3	1	1	1	1	0	1	0	0	1	1	0	1	0	1	1	1	0	1	1	0
	4	0	0	0	1	0	0	0	0	0	0	0	0	0	1	1	0	0	1	0	0
	5	0	0	1	1	0	0	0	0	0	0	0	0	0	1	1	1	0	0	0	0
VLA	6	1	1	0	1	1	0	0	0	1	1	0	0	0	1	1	1	0	0	1	0
	7	0	1	0	0	1	0	0	0	1	0	0	0	0	0	0	0	0	0	1	0
	8	1	1	1	1	1	0	0	0	0	0	0	0	0	0	1	1	0	1	0	0
	9	0	0	1	1	0	0	0	0	1	0	0	0	0	0	0	1	0	1	0	0
	10	1	0	0	0	1	1	0	0	0	1	0	1	0	0	1	0	1	1	0	0
	11	0	1	1	1	1	1	0	0	1	1	0	1	0	1	1	1	1	0	1	0

Supplementary Table S1. Continued.

Site	Ind.	Locus																			
		21	22	23	24	25	26	27	28	29	30	31	32	33	34	35	36	37	38	39	40
PNN	1	0	1	0	1	0	1	1	0	1	1	0	0	0	1	1	1	0	0	1	0
	2	0	1	0	1	1	1	1	1	1	1	1	1	0	1	1	1	0	1	1	1
	3	0	1	0	0	0	0	1	0	1	1	0	0	0	0	1	0	0	0	0	0
	4	1	1	0	0	1	0	1	0	1	1	1	0	0	0	1	1	0	0	1	1
	5	0	1	0	0	1	0	1	0	1	1	0	0	0	0	1	0	0	0	1	1
	6	0	1	0	0	1	0	1	0	1	1	0	0	0	0	0	0	0	0	0	1
	7	1	0	0	1	1	0	1	1	1	1	1	1	0	0	1	1	0	0	0	1
	8	1	1	0	1	1	0	1	1	1	0	1	1	0	1	0	1	0	1	1	0
	9	1	1	0	1	1	0	1	0	0	0	0	1	0	1	1	1	0	1	1	1
	10	1	1	0	1	1	0	1	0	0	0	0	0	0	0	0	1	0	0	0	1
	11	1	1	0	0	1	0	1	0	0	0	0	1	0	1	1	1	0	1	1	1
	12	1	0	0	0	1	0	1	1	1	0	0	0	0	0	1	1	0	1	0	1
	13	1	1	0	1	1	0	1	0	0	0	0	1	0	0	1	1	0	1	1	1
	14	1	1	0	1	1	0	1	1	1	0	0	1	0	1	1	1	0	1	1	1
	15	1	1	0	1	1	1	1	0	1	0	0	0	0	1	0	1	0	1	1	1
VLA	1	1	0	0	0	1	0	0	0	0	0	1	1	0	0	0	0	0	1	0	1
	2	1	1	0	0	1	0	0	1	0	0	0	0	0	1	0	0	0	1	1	1
	3	1	1	0	1	1	0	1	0	1	1	1	1	0	1	1	0	0	0	1	1
	4	0	0	0	0	1	0	1	0	1	0	1	1	0	1	0	0	0	0	0	0
	5	1	0	0	0	0	0	0	0	0	1	0	0	0	0	0	0	0	0	0	0
	6	1	0	0	0	1	0	0	1	0	0	1	0	0	1	0	0	0	1	0	1
	7	0	0	0	1	1	0	0	1	1	0	0	1	0	0	1	1	0	0	1	1
	8	0	1	0	1	1	0	0	1	0	0	0	0	0	0	0	0	0	0	0	0
	9	0	0	0	0	0	0	1	0	0	0	0	0	0	0	0	0	0	0	1	0
	10	0	1	0	0	0	0	1	1	0	1	0	0	0	0	1	0	0	0	1	1
	11	1	1	0	0	1	0	1	1	1	1	1	1	0	0	1	1	0	1	1	1

Supplementary Table S1. Continued.

Site	Ind.	Locus																			
		41	42	43	44	45	46	47	48	49	50	51	52	53	54	55	56	57	58	59	60
PNN	1	0	0	1	1	1	1	0	0	1	0	0	0	1	1	1	0	1	0	1	1
	2	0	0	0	1	1	1	1	1	1	0	1	1	1	1	1	1	1	0	0	1
	3	1	0	0	1	1	0	0	1	0	0	0	1	1	0	1	1	1	1	1	0
	4	0	0	0	1	1	1	1	1	1	0	0	1	1	1	1	0	1	0	0	1
	5	1	0	1	1	1	1	1	1	0	0	0	1	1	0	1	1	1	0	0	1
	6	1	0	1	1	1	1	0	1	0	0	0	1	1	0	0	1	0	1	0	1
	7	0	0	0	1	1	1	1	0	0	0	1	0	1	1	1	1	1	1	1	0
	8	0	0	0	1	1	0	0	1	1	0	1	1	1	1	0	1	1	0	0	0
	9	0	0	1	1	1	0	1	0	1	0	0	1	1	1	0	1	0	0	0	0
	10	1	0	1	1	1	1	0	1	1	0	0	1	0	1	1	1	0	0	0	0
	11	0	0	0	1	1	1	1	0	1	0	0	1	1	1	0	1	0	0	0	0
	12	1	0	0	1	1	0	1	1	1	0	0	1	1	1	0	1	1	0	0	0
	13	1	0	0	1	1	0	0	0	0	0	0	1	1	1	0	1	1	1	0	0
	14	1	0	0	1	1	0	1	0	0	0	1	1	0	1	1	1	1	0	0	0
	15	0	0	0	1	1	0	0	1	0	0	0	0	1	0	0	1	0	0	0	0
VLA	1	1	0	1	1	1	1	1	1	1	0	1	1	0	1	1	1	0	0	1	0
	2	0	0	0	0	0	0	1	1	1	0	1	1	1	0	0	0	1	0	0	0
	3	1	0	1	1	0	1	1	0	1	0	0	1	1	1	1	1	1	0	1	0
	4	0	0	0	0	0	0	0	0	1	0	0	0	0	0	0	0	0	0	0	0
	5	0	0	0	1	0	0	1	0	0	0	1	1	1	0	0	1	0	0	0	0
	6	1	0	1	1	1	1	1	0	1	0	1	1	1	1	1	1	1	1	1	0
	7	0	0	0	1	0	0	0	0	0	0	1	1	1	0	1	0	0	0	0	0
	8	0	0	1	0	1	0	0	0	0	0	1	1	1	0	0	1	1	0	0	0
	9	0	0	1	1	1	1	0	0	0	0	0	0	1	1	0	0	0	1	0	0
	10	0	0	0	1	1	1	0	1	1	0	1	0	1	0	0	0	1	1	1	1
	11	0	0	0	1	1	1	0	0	1	0	1	1	1	1	1	1	1	0	0	0

Supplementary Table S1. Continued.

Site	Ind.	Locus																			
		61	62	63	64	65	66	67	68	69	70	71	72	73	74	75	76	77	78	79	80
PNN	1	0	0	0	0	0	1	0	0	0	1	0	0	1	0	1	0	0	0	0	1
	2	0	1	1	1	1	1	0	1	1	1	1	1	1	0	1	0	0	0	0	1
	3	0	0	1	0	1	0	0	0	1	0	0	1	0	0	0	0	0	0	1	0
	4	0	0	1	0	0	1	0	0	0	0	0	1	0	0	1	0	0	0	0	1
	5	0	0	1	0	0	1	0	0	0	1	0	1	0	0	0	0	0	0	1	1
	6	0	0	0	0	0	1	0	0	0	0	0	1	0	0	0	0	0	0	0	0
	7	0	1	1	0	1	1	0	0	0	0	1	1	0	0	0	1	0	0	0	1
	8	0	0	1	1	1	1	0	1	0	0	0	1	1	0	1	0	0	0	1	0
	9	0	0	0	0	1	1	0	0	0	0	1	1	1	0	0	0	0	0	1	0
	10	0	1	1	1	1	1	0	0	0	0	0	0	0	0	0	0	0	0	0	1
	11	0	1	1	1	0	1	0	0	0	0	1	1	1	0	0	0	0	0	1	1
	12	0	1	1	1	1	1	0	1	0	0	1	0	1	0	1	1	0	0	1	0
	13	0	1	0	1	0	1	0	0	0	0	1	1	1	0	0	0	0	0	0	0
	14	0	0	1	1	1	1	0	0	0	0	1	1	1	0	0	0	0	0	1	0
	15	0	1	0	1	1	1	0	1	0	0	1	0	1	0	0	0	0	0	1	0
VLA	1	0	0	1	0	1	0	0	0	0	0	1	1	1	0	1	0	0	0	1	1
	2	0	1	1	0	1	1	0	0	0	0	0	0	0	0	0	0	0	0	0	0
	3	0	1	1	0	0	0	0	0	1	0	1	0	1	0	1	0	0	0	1	0
	4	0	1	1	1	1	1	0	0	0	0	1	0	0	0	0	1	0	0	0	1
	5	0	1	0	0	0	0	0	0	0	0	0	1	0	0	1	0	0	0	0	0
	6	0	0	0	0	1	1	0	1	0	0	1	1	1	0	1	1	0	0	1	1
	7	0	1	1	0	1	1	0	0	0	0	0	1	0	0	0	0	0	0	0	0
	8	0	1	1	0	1	0	0	0	0	0	0	0	0	0	0	0	0	0	0	0
	9	0	0	0	0	0	0	0	0	0	0	0	0	0	0	0	0	0	0	0	1
	10	0	0	0	0	0	1	0	1	0	0	0	0	0	0	0	0	0	0	0	1
	11	0	1	1	0	1	1	0	0	0	0	1	1	1	0	1	0	0	0	1	0

Supplementary Table S1. Continued.

Site	Ind.	Locus																			
		81	82	83	84	85	86	87	88	89	90	91	92	93	94	95	96	97	98	99	100
	1	0	0	0	1	1	1	1	1	0	1	1	1	1	1	1	1	1	1	1	1
	2	1	0	1	1	1	1	1	1	0	1	1	1	1	1	0	1	1	1	0	1
	3	1	0	1	1	0	0	1	0	0	1	0	0	1	1	0	1	1	1	0	0
	4	0	0	1	0	1	1	0	0	0	1	0	0	1	1	1	1	0	1	1	1
	5	1	0	1	1	1	1	0	0	1	1	0	0	1	1	0	1	1	1	0	1
	6	0	0	1	1	1	1	0	0	0	1	0	0	1	1	0	1	1	1	1	1
	7	1	0	0	0	1	1	0	0	1	0	1	1	0	1	1	1	1	1	0	1
PNN	8	1	0	0	0	1	0	0	0	1	0	0	0	1	1	1	0	0	1	1	0
	9	1	0	1	1	0	0	0	0	1	0	0	1	1	1	1	0	1	1	0	1
	10	1	0	1	1	1	0	0	1	0	0	1	1	1	0	1	0	0	0	1	0
	11	1	0	1	1	0	0	0	0	1	0	0	1	0	1	1	1	1	1	1	1
	12	1	0	0	1	0	0	0	0	1	0	0	1	0	1	1	1	0	1	0	1
	13	1	0	1	1	0	0	0	0	1	0	1	1	0	1	1	1	1	1	1	0
	14	1	0	1	1	1	0	0	0	1	0	0	1	0	1	1	1	1	1	1	0
	15	0	0	0	1	0	0	0	0	1	0	0	1	0	0	1	0	1	1	0	0
	1	1	0	0	0	1	0	0	1	1	0	1	1	0	1	1	1	1	0	1	0
	2	1	0	0	0	0	0	0	0	1	0	0	1	0	0	1	1	0	1	0	0
	3	1	0	1	1	1	0	0	1	1	1	0	1	1	1	1	1	1	1	1	1
	4	0	0	0	0	0	0	0	1	0	0	0	1	0	0	1	1	0	0	0	0
	5	0	0	0	0	1	1	0	0	0	0	0	0	0	1	0	1	0	1	1	1
VLA	6	1	0	0	1	1	0	0	1	1	1	1	1	1	0	1	1	1	0	0	1
	7	1	0	0	1	1	0	0	0	1	0	1	1	0	1	1	0	0	1	0	0
	8	0	0	0	0	0	0	0	0	0	0	0	1	0	0	0	0	0	0	1	1
	9	0	0	0	0	0	0	0	0	0	0	0	0	0	1	0	0	0	0	0	0
	10	0	0	1	0	0	1	0	0	0	1	1	0	1	1	0	1	0	0	0	1
	11	1	0	1	0	0	0	0	1	1	0	1	1	0	1	1	0	1	1	1	1

Supplementary Table S1. Continued.

Site	Ind.	Locus																			
		101	102	103	104	105	106	107	108	109	110	111	112	113	114	115	116	117	118	119	120
PNN	1	0	1	1	1	1	0	0	1	1	0	0	0	0	0	1	0	1	1	0	1
	2	0	1	1	0	1	1	1	0	1	1	1	0	1	1	1	0	1	1	1	0
	3	1	0	1	0	1	0	0	1	1	0	0	0	0	1	0	1	1	0	0	1
	4	1	1	1	0	1	0	0	1	0	0	1	0	0	1	0	0	1	1	0	1
	5	1	1	1	0	1	1	0	0	1	0	1	0	0	1	0	1	1	0	0	1
	6	1	1	1	0	1	0	0	0	0	0	0	0	0	1	0	1	1	1	0	1
	7	1	1	0	1	0	0	1	0	1	1	0	0	1	1	1	1	1	1	1	1
	8	0	1	0	0	0	1	1	0	1	1	1	0	0	1	0	1	0	1	0	1
	9	0	1	0	1	0	0	0	0	1	0	0	0	0	0	1	1	0	1	0	1
	10	1	1	0	0	1	0	0	1	1	0	0	0	0	1	0	1	0	1	0	1
	11	1	1	0	1	0	0	1	0	1	1	0	0	1	1	1	1	1	1	0	1
	12	0	1	0	1	1	0	0	1	1	0	0	0	1	1	1	1	1	1	0	1
	13	1	1	0	1	0	0	1	0	1	0	0	0	1	0	1	1	1	1	0	1
	14	1	1	0	1	0	1	1	0	1	1	0	0	1	1	1	1	1	1	0	1
	15	1	1	0	0	1	0	0	0	0	0	0	0	1	0	1	1	0	1	0	1
VLA	1	0	1	1	1	0	1	0	0	1	1	0	0	0	0	0	1	1	1	0	1
	2	1	1	0	1	0	0	0	0	0	0	0	0	0	1	0	1	0	1	0	1
	3	1	1	0	1	0	0	0	1	1	0	0	0	0	1	1	1	1	1	0	1
	4	0	0	1	1	0	0	0	0	1	1	0	0	0	1	0	0	0	0	1	1
	5	1	0	0	0	0	0	0	0	1	0	0	0	0	0	1	1	0	1	0	1
	6	1	1	1	1	1	0	1	1	1	1	1	0	0	0	1	0	1	1	1	1
	7	0	0	0	1	0	0	1	1	0	0	0	0	0	0	0	1	0	0	0	1
	8	0	0	0	1	0	0	0	0	1	1	0	0	0	1	1	1	0	1	0	1
	9	0	0	0	0	1	0	0	1	1	0	0	0	0	0	0	1	0	1	0	0
	10	0	0	1	0	1	1	0	0	0	0	1	0	0	1	1	1	0	0	0	1
	11	1	1	0	1	0	0	0	0	1	1	0	0	1	0	1	1	1	1	0	1

Supplementary Table S1. Continued.

Site	Ind.	Locus																			
		121	122	123	124	125	126	127	128	129	130	131	132	133	134	135	136	137	138	139	140
PNN	1	1	1	0	0	0	0	1	0	0	0	0	0	0	0	1	0	0	1	0	0
	2	1	1	0	1	1	1	1	1	0	0	0	0	0	0	1	1	0	0	0	0
	3	0	0	0	0	1	1	1	1	0	1	1	1	1	0	0	0	0	1	1	0
	4	0	1	0	0	1	1	1	1	0	1	1	1	0	0	1	0	0	1	1	0
	5	1	0	0	0	0	1	1	1	0	1	1	1	1	1	1	0	0	1	1	0
	6	1	0	0	1	0	1	1	0	0	1	1	1	1	1	0	0	0	1	1	0
	7	1	1	0	0	1	1	0	1	0	1	0	1	0	1	0	0	0	1	1	0
	8	0	1	0	1	1	1	1	0	0	0	0	0	0	1	0	0	0	1	1	0
	9	0	1	0	0	1	1	0	0	0	0	0	0	0	0	0	0	0	0	0	0
	10	0	1	0	0	0	1	0	0	0	1	1	1	1	0	1	1	0	0	0	0
	11	1	1	0	0	1	1	1	1	0	1	1	0	0	1	0	0	0	1	1	0
	12	0	1	0	0	1	1	1	1	0	1	1	1	0	1	1	0	0	1	1	0
	13	1	1	0	0	1	1	1	1	0	1	1	0	0	0	1	0	0	1	1	0
	14	1	1	0	0	1	1	0	1	0	1	1	1	0	0	1	0	0	1	1	0
	15	0	1	0	1	1	1	0	1	0	1	0	0	0	0	1	0	0	1	0	0
VLA	1	1	1	0	0	1	1	0	0	0	1	1	0	1	1	0	1	0	1	1	0
	2	0	1	0	0	1	0	0	0	0	1	0	0	0	0	0	0	0	1	0	0
	3	1	1	0	0	1	1	1	1	0	1	0	0	1	1	1	1	0	1	0	0
	4	1	1	0	0	0	0	1	0	0	0	1	1	0	0	0	0	0	0	0	0
	5	1	1	0	0	0	1	1	0	0	0	1	1	0	1	1	0	0	1	1	0
	6	1	1	0	1	0	0	0	0	0	1	0	1	1	1	1	1	0	0	1	0
	7	0	1	0	0	0	1	0	0	0	0	0	0	1	1	0	0	0	0	0	0
	8	0	1	0	0	0	1	0	0	0	0	0	0	0	0	0	0	0	1	0	0
	9	0	1	0	0	0	1	0	0	0	0	0	0	0	1	0	0	0	0	0	0
	10	0	1	0	1	1	1	1	0	0	0	0	0	0	1	0	0	0	1	0	0
	11	1	1	0	0	1	1	1	1	0	0	1	0	0	0	0	0	0	1	1	0

Supplementary Table S1. Continued.

Site	Ind.	Locus																			
		141	142	143	144	145	146	147	148	149	150	151	152	153	154	155	156	157	158	159	160
	1	0	0	0	1	0	0	0	0	0	0	1	1	1	1	0	0	1	1	0	0
	2	0	0	1	1	0	0	0	0	1	1	0	0	0	0	0	0	0	1	1	0
	3	0	0	0	1	0	0	1	0	0	0	0	0	0	1	0	0	0	1	0	0
	4	0	0	0	0	0	0	1	0	0	0	0	1	1	1	1	0	1	1	1	0
	5	0	0	1	0	0	0	1	0	0	0	0	1	1	1	1	0	1	1	0	0
	6	0	0	1	0	0	0	1	0	0	0	0	0	1	1	1	0	1	1	1	0
	7	0	0	0	1	0	0	0	1	0	1	1	1	0	0	1	1	1	0	0	1
PNN	8	1	0	1	0	0	0	0	1	0	0	1	1	0	0	0	0	1	0	0	0
	9	0	0	0	0	0	0	0	0	0	0	1	1	0	0	1	0	0	0	0	1
	10	0	0	0	0	0	0	0	1	1	1	1	1	1	1	1	1	0	0	1	1
	11	0	0	1	0	0	0	0	1	0	0	1	1	1	1	1	1	0	1	0	1
	12	0	0	1	0	0	0	0	1	0	0	1	1	0	1	1	1	0	1	0	1
	13	0	0	1	1	0	0	0	1	0	0	1	1	0	1	1	1	1	0	0	0
	14	0	0	0	1	0	0	0	0	0	1	1	1	0	1	1	0	0	0	0	1
	15	0	0	0	0	0	0	1	1	0	1	1	1	0	1	1	1	0	1	0	1
	1	0	0	1	1	1	0	0	1	0	0	1	1	0	1	0	0	0	0	0	0
	2	0	0	0	0	0	0	0	1	0	0	1	1	1	0	0	0	0	0	0	1
	3	0	0	1	1	0	0	0	0	0	0	1	1	0	1	0	1	0	0	0	0
	4	0	0	0	0	1	0	0	1	1	1	1	1	1	0	1	1	0	0	1	0
	5	0	0	1	0	1	0	0	0	0	0	1	1	1	0	0	1	0	0	0	0
VLA	6	0	0	1	1	1	0	0	1	0	0	1	1	0	0	1	0	1	0	0	1
	7	0	0	0	1	0	0	0	0	0	0	1	1	1	0	0	0	0	0	0	0
	8	1	0	0	0	0	0	0	0	0	0	1	1	0	1	1	0	0	0	1	1
	9	0	0	0	0	1	0	0	0	0	0	0	1	1	1	0	0	1	0	0	0
	10	0	0	0	1	0	0	1	0	1	0	0	1	1	0	0	0	1	1	1	0
	11	0	0	0	1	0	0	0	1	0	1	1	1	0	1	1	1	0	0	0	1

Supplementary Table S1. Continued.

Site	Ind.	Locus																			
		161	162	163	164	165	166	167	168	169	170	171	172	173	174	175	176	177	178	179	180
	1	0	1	1	1	1	0	0	1	1	1	1	1	0	0	0	1	0	0	0	0
	2	1	0	0	0	0	0	1	0	0	0	0	0	0	0	0	1	0	0	0	1
	3	0	0	1	0	1	0	0	1	0	0	0	0	0	0	0	0	0	0	0	1
	4	0	1	1	1	1	0	0	1	0	0	0	1	1	1	1	1	1	1	1	1
	5	0	0	1	1	1	0	0	1	0	0	0	1	1	0	1	1	0	1	0	0
	6	1	0	1	1	1	0	0	1	1	1	0	1	1	0	1	1	0	0	0	0
	7	0	1	1	0	1	1	0	1	0	1	0	1	1	1	1	1	1	0	0	0
PNN	8	0	1	0	0	1	1	1	0	1	1	1	1	0	1	0	0	0	0	0	0
	9	1	1	0	0	0	0	1	0	1	1	0	1	1	1	0	0	0	0	0	0
	10	0	1	1	1	1	1	1	0	0	1	0	1	1	1	1	1	0	0	0	1
	11	0	1	0	0	1	1	1	1	1	1	1	1	1	1	1	1	1	0	0	0
	12	1	1	0	0	0	1	1	0	0	1	0	0	1	1	1	1	0	0	0	0
	13	1	1	1	0	1	1	1	0	1	1	0	1	0	1	1	1	1	0	0	0
	14	1	1	0	0	1	0	1	0	1	1	1	1	1	1	1	1	0	0	0	0
	15	0	1	0	0	0	1	1	1	1	1	1	1	0	1	0	0	0	0	0	1
	1	1	1	1	0	0	0	1	1	1	0	0	1	1	1	1	1	0	0	0	0
	2	1	1	0	0	0	0	1	0	1	1	0	0	1	1	1	0	0	0	0	0
	3	1	1	0	0	0	0	1	0	0	0	1	1	1	1	1	0	0	0	0	0
	4	0	0	0	0	0	1	1	0	0	1	1	0	1	1	1	0	0	0	1	1
	5	1	0	0	1	1	1	1	1	1	1	0	1	1	1	1	1	0	0	1	0
VLA	6	1	1	0	0	0	1	1	1	0	1	1	1	1	1	1	1	0	0	0	0
	7	1	0	0	0	0	0	0	1	0	1	0	1	0	0	0	0	0	0	0	0
	8	1	0	0	0	0	0	0	0	0	0	0	0	0	0	0	0	0	0	0	0
	9	0	0	1	1	1	0	0	1	1	0	0	0	0	0	0	0	0	0	0	0
	10	0	0	1	1	0	0	0	0	0	0	0	1	0	0	0	1	1	0	0	0
	11	1	1	0	0	1	1	1	0	1	1	1	1	1	1	1	1	0	0	0	0

Supplementary Table S1. Continued.

Site	Ind.	Locus																			
		181	182	183	184	185	186	187	188	189	190	191	192	193	194	195	196	197	198	199	200
	1	0	0	1	0	1	0	0	1	1	1	1	0	0	1	1	1	1	1	1	0
	2	1	0	1	0	0	0	0	0	0	1	1	0	1	1	0	0	0	0	0	0
	3	1	0	0	1	1	0	0	0	0	1	1	0	0	1	0	0	0	1	0	0
	4	1	0	0	1	1	0	0	0	1	0	1	1	1	1	0	0	1	1	1	0
	5	1	0	0	1	1	0	0	0	1	1	1	1	1	1	0	0	1	1	1	0
	6	1	0	0	1	1	0	0	0	0	1	1	1	0	1	0	1	1	1	1	0
	7	1	1	0	0	0	1	1	1	0	1	0	1	0	1	1	0	1	0	0	0
PNN	8	0	1	0	1	0	0	1	1	1	1	1	0	0	0	1	0	1	0	0	0
	9	0	1	0	0	1	1	1	1	1	0	0	0	0	1	1	0	1	1	0	0
	10	1	1	0	0	0	1	0	1	1	1	0	0	0	1	1	1	1	0	0	1
	11	1	1	0	1	1	1	1	1	1	1	1	1	0	1	1	0	1	1	0	1
	12	1	1	1	0	1	0	1	1	1	1	1	1	1	1	1	0	1	1	0	1
	13	1	1	0	0	1	0	1	1	0	1	1	1	1	1	1	0	0	0	0	1
	14	1	1	0	0	1	1	1	1	0	0	1	1	1	1	1	0	1	0	0	0
	15	1	1	0	0	1	0	1	1	1	1	1	1	0	1	1	0	1	1	0	1
	1	1	1	1	0	0	0	1	0	0	1	1	0	0	0	1	0	0	1	0	1
	2	1	1	1	0	1	0	0	1	1	0	1	1	0	1	1	1	0	0	0	1
	3	1	1	1	1	1	0	1	1	1	1	0	1	1	0	1	0	1	1	0	1
	4	1	1	1	1	0	1	1	0	0	1	1	1	1	0	1	0	0	0	1	0
	5	1	1	0	0	0	1	1	1	1	1	0	0	0	0	0	0	1	0	1	0
VLA	6	1	1	0	0	1	1	0	1	0	0	1	1	0	1	1	0	1	0	0	1
	7	1	1	0	1	0	0	0	1	0	0	0	1	0	1	1	0	0	1	0	0
	8	1	1	0	0	0	0	1	0	0	0	0	0	0	0	0	0	0	0	0	0
	9	1	0	0	1	1	1	0	0	0	1	1	0	0	0	0	0	1	1	0	0
	10	1	1	0	1	1	0	0	1	1	0	1	1	0	1	1	1	1	1	0	0
	11	1	1	0	0	1	0	1	1	0	0	0	1	1	1	1	0	0	1	0	0

Supplementary Table S1. Continued.

Site	Ind.	Locus																			
		201	202	203	204	205	206	207	208	209	210	211	212	213	214	215	216	217	218	219	220
PNN	1	0	0	0	0	1	0	1	1	0	0	0	0	0	0	0	0	1	0	1	1
	2	0	0	0	0	1	1	1	1	0	0	0	0	0	0	0	0	0	0	0	1
	3	0	0	0	0	1	1	1	0	0	0	1	0	0	0	0	0	0	0	0	1
	4	0	0	0	0	1	1	1	1	0	0	1	0	0	0	0	0	0	0	1	1
	5	1	0	0	1	1	1	1	1	0	0	1	0	0	1	0	1	1	0	1	1
	6	1	0	0	0	1	1	1	1	0	0	1	0	0	0	0	1	1	0	1	1
	7	0	1	0	1	1	0	0	1	0	0	0	1	0	0	1	1	1	1	1	1
	8	1	1	0	1	1	1	0	0	0	0	0	1	0	0	1	0	1	1	1	1
	9	1	1	0	1	1	0	0	1	0	0	1	0	0	0	1	0	1	1	1	1
	10	0	1	0	1	0	1	1	1	0	1	0	1	0	0	0	0	1	0	1	1
	11	1	1	1	1	1	1	1	1	0	0	1	1	0	0	1	0	0	1	1	1
	12	1	1	0	1	1	0	0	1	0	0	1	1	0	0	1	1	1	0	1	1
	13	1	1	1	1	1	0	0	1	0	0	1	1	0	0	1	0	1	0	1	1
	14	1	1	1	1	1	1	0	1	0	0	1	0	0	0	1	0	1	0	1	1
	15	1	1	0	0	1	0	0	1	1	1	0	0	0	0	1	0	1	0	1	1
VLA	1	0	1	1	1	1	0	0	0	0	0	1	0	0	0	0	1	0	1	0	1
	2	1	1	0	1	1	0	0	0	0	0	1	1	0	0	1	0	0	1	1	1
	3	1	1	0	0	1	1	0	1	0	0	0	1	0	0	1	1	1	1	1	1
	4	1	0	0	0	1	0	0	0	0	0	0	0	0	0	1	1	0	1	1	1
	5	0	1	0	0	0	0	0	0	0	0	0	0	1	1	1	1	0	0	0	1
	6	1	1	1	1	1	0	0	1	0	0	1	0	0	0	1	1	1	0	1	1
	7	0	0	0	0	1	0	0	1	0	0	0	0	0	0	1	0	1	0	0	1
	8	0	1	0	0	0	0	0	0	0	0	1	1	0	0	1	0	1	1	0	1
	9	0	0	0	0	1	0	0	1	0	0	0	1	1	1	0	1	1	0	0	1
	10	1	0	1	0	1	0	1	1	0	0	1	1	0	0	0	0	1	1	1	1
	11	1	1	1	1	1	0	0	1	0	0	1	0	0	0	1	1	0	1	1	1

Supplementary Table S1. Continued.

Site	Ind.	Locus																			
		221	222	223	224	225	226	227	228	229	230	231	232	233	234	235	236	237	238	239	240
PNN	1	1	1	1	1	0	0	0	0	1	0	1	0	1	0	1	0	1	1	1	0
	2	0	0	1	1	1	1	0	0	1	0	1	0	1	0	0	0	1	1	0	1
	3	1	0	1	0	1	0	0	0	1	1	1	0	1	0	1	0	1	1	1	1
	4	1	0	1	1	0	0	0	0	1	1	1	0	1	0	1	0	1	1	1	0
	5	1	1	1	1	1	0	0	0	1	1	1	0	1	0	1	0	1	1	1	0
	6	1	0	1	1	0	0	0	0	1	1	1	0	1	0	1	0	1	1	1	0
	7	1	1	1	1	0	0	0	1	1	1	0	0	0	1	0	1	1	1	0	1
	8	1	1	0	1	1	0	0	1	0	1	0	0	0	0	0	0	1	1	0	1
	9	1	1	0	1	0	0	0	1	1	1	0	0	0	1	0	1	1	1	0	1
	10	1	1	1	1	1	0	0	1	1	1	0	1	0	1	0	0	1	0	0	1
	11	1	1	0	1	1	0	0	1	1	1	0	0	0	1	0	1	1	1	0	1
	12	0	1	0	1	0	0	0	1	1	1	0	0	0	1	0	1	1	1	0	1
	13	1	1	0	0	1	0	0	1	0	1	0	1	0	1	0	0	1	1	0	1
	14	1	1	0	1	1	0	0	1	1	1	1	1	1	1	0	1	1	1	0	1
	15	1	1	0	0	0	0	0	1	1	1	0	1	1	1	0	0	1	1	0	1
VLA	1	1	1	0	0	0	0	0	1	1	1	1	0	1	1	0	1	1	0	0	1
	2	1	1	0	1	0	0	0	1	0	1	1	0	0	1	0	0	1	1	1	1
	3	1	1	1	1	1	0	0	1	1	1	1	1	1	1	1	1	1	1	1	1
	4	0	0	0	0	0	0	1	0	0	1	1	0	0	1	0	0	1	1	0	1
	5	0	0	0	0	1	0	0	1	0	1	0	0	0	0	0	0	0	0	0	1
	6	1	1	0	0	0	0	0	1	1	1	0	0	0	1	0	1	1	0	0	1
	7	0	0	1	0	1	0	0	1	0	1	0	0	1	1	0	0	1	0	1	1
	8	0	0	0	0	0	0	0	0	0	1	0	0	1	1	0	0	1	0	1	0
	9	1	0	1	1	0	0	0	0	1	1	1	0	0	0	0	0	0	0	0	0
	10	1	0	1	1	0	0	0	0	1	0	1	0	1	0	0	1	0	1	1	0
	11	1	1	0	0	1	0	0	1	1	1	1	0	0	1	0	1	1	1	0	1

Supplementary Table S1. Continued.

Site	Ind.	Locus																			
		241	242	243	244	245	246	247	248	249	250	251	252	253	254	255	256	257	258	259	260
PNN	1	1	0	0	1	0	0	0	1	0	1	1	1	0	1	0	1	1	1	1	0
	2	1	0	1	0	1	0	0	1	0	1	1	1	0	1	0	0	0	1	1	0
	3	1	1	0	0	0	1	1	1	0	1	0	1	0	1	0	1	1	0	1	0
	4	1	1	0	0	1	1	1	1	0	1	1	1	0	1	0	0	1	1	1	0
	5	1	0	1	1	0	1	1	1	0	1	1	1	1	1	0	0	1	0	1	0
	6	1	0	1	1	0	1	0	1	0	1	1	1	0	1	0	0	1	1	1	0
	7	0	1	0	0	1	1	1	0	1	1	1	0	1	1	1	1	0	1	1	0
	8	1	0	0	0	0	1	1	1	1	1	1	0	1	0	1	1	0	1	1	0
	9	0	1	1	0	1	1	1	0	1	1	1	0	0	1	1	1	0	1	1	0
	10	0	1	0	0	1	1	1	0	1	1	1	0	0	1	1	1	0	1	1	1
	11	1	1	1	0	1	1	1	0	1	1	1	0	0	1	1	1	0	1	0	0
	12	1	1	1	0	1	1	1	0	1	1	1	0	0	1	1	1	0	1	1	0
	13	1	1	1	0	1	0	1	0	1	1	1	0	1	1	1	1	0	1	1	0
	14	1	1	0	1	1	0	1	0	1	1	1	0	0	1	1	1	0	1	1	0
	15	0	0	0	0	1	1	1	0	1	1	1	0	0	1	1	0	0	1	1	0
VLA	1	1	0	1	0	1	1	1	1	1	0	1	0	0	1	1	1	0	1	1	1
	2	1	1	1	0	1	1	1	0	0	1	1	0	0	1	1	1	0	1	1	0
	3	1	1	0	0	1	1	1	0	1	0	1	0	1	1	1	1	0	1	0	0
	4	0	1	0	0	0	1	1	0	0	0	1	0	0	1	1	1	0	1	1	0
	5	0	0	0	0	0	1	1	0	1	0	1	0	0	1	1	0	0	1	1	0
	6	1	1	0	0	1	1	1	0	1	1	1	0	1	1	1	1	0	1	1	0
	7	0	0	0	0	0	1	1	0	1	1	1	0	0	1	1	0	0	1	1	0
	8	1	0	0	1	0	1	1	0	1	0	1	0	0	1	1	1	0	1	1	0
	9	0	0	0	0	0	0	0	0	1	1	0	1	0	0	0	0	1	0	1	0
	10	1	0	0	0	0	1	0	1	1	1	0	1	0	1	0	0	0	1	1	0
	11	0	1	0	0	1	1	1	0	1	1	1	0	0	1	1	0	0	1	1	0

Supplementary Table S1. Continued.

Site	Ind.	Locus 261	262	263	264	265	266	267	268
PNN	1	0	0	1	0	1	0	0	1
	2	1	1	1	1	1	0	0	0
	3	0	0	1	0	1	0	1	1
	4	1	0	1	0	1	0	1	1
	5	0	1	1	0	1	0	1	1
	6	0	0	1	1	1	1	1	1
	7	1	0	0	1	1	0	1	1
	8	1	0	0	1	0	0	0	1
	9	0	0	0	1	0	0	0	1
	10	0	0	0	1	0	0	0	1
	11	1	0	0	1	0	0	1	1
	12	1	0	0	1	0	0	0	1
	13	1	0	0	1	1	0	0	1
	14	1	0	0	1	0	0	1	1
	15	1	0	0	1	0	0	1	1
VLA	1	0	0	1	0	0	1	1	0
	2	0	0	1	0	0	1	1	0
	3	0	0	1	0	0	1	1	0
	4	0	0	1	0	0	0	1	0
	5	1	0	1	0	0	0	1	1
	6	0	0	1	0	0	1	1	0
	7	0	0	1	0	0	1	1	0
	8	0	0	1	0	0	0	0	0
	9	0	0	0	1	0	0	0	0
	10	0	0	1	1	0	0	1	0
	11	0	0	1	0	0	1	1	0

Supplementary Table S1. Continued.

Site	Ind.	Locus																			
		1	2	3	4	5	6	7	8	9	10	11	12	13	14	15	16	17	18	19	20
VLA	12	1	1	1	1	1	1	0	0	1	1	0	1	0	1	1	1	1	0	1	0
	13	0	1	1	1	1	1	0	0	1	1	0	1	0	1	1	1	1	0	1	0
	14	1	1	0	1	0	1	0	0	0	0	0	0	0	1	0	1	0	0	0	0
	15	1	1	1	1	1	1	0	0	1	1	0	1	0	0	1	1	1	1	1	0
	16	0	1	1	1	1	1	0	0	1	1	0	1	0	1	0	1	1	0	1	0
	17	0	0	1	1	1	0	0	0	1	0	0	0	0	1	0	1	1	0	1	0
	18	1	1	1	1	0	1	0	0	1	1	0	1	0	1	1	1	0	0	0	0
	19	0	1	1	1	1	1	0	0	1	1	0	1	0	1	1	1	1	0	1	0
	20	0	1	1	1	1	1	0	0	1	1	0	1	0	1	1	1	1	0	1	0
	21	0	1	1	1	1	1	0	0	1	1	0	1	0	1	0	0	1	1	0	0
	22	1	0	0	1	0	0	0	0	1	1	0	1	0	1	1	0	1	0	0	0
	23	0	0	1	1	1	1	0	0	1	1	0	1	0	1	0	1	1	0	1	0
	24	0	1	0	1	1	0	0	0	1	1	0	1	0	1	0	1	1	0	1	0
	25	1	1	0	1	1	1	0	0	1	1	0	0	0	1	1	1	1	1	1	0
	26	0	1	0	1	1	1	0	0	1	1	0	1	0	1	1	1	1	0	1	0
	27	1	1	0	1	1	0	0	0	1	1	0	0	0	1	1	1	0	0	1	0
	28	0	1	1	1	1	1	0	0	1	1	0	0	0	1	1	1	0	0	1	0
	29	1	1	0	1	1	0	0	0	1	1	0	1	0	1	0	1	1	1	1	0
	30	1	1	0	1	1	1	0	0	1	1	0	1	0	1	1	1	1	0	1	0
	31	1	1	0	1	1	1	0	0	1	1	0	1	0	1	0	1	0	0	1	0
	32	1	0	0	0	1	1	0	0	1	1	0	0	0	0	1	1	0	0	0	0
CAÑ	1	1	1	1	1	0	0	0	0	1	0	0	0	0	1	1	0	1	0	1	1
	2	0	0	0	0	0	0	0	0	0	0	0	0	0	0	0	0	0	0	0	0
	3	0	0	0	0	0	0	0	0	0	0	0	0	0	0	0	0	0	0	0	0
	4	0	0	0	0	0	0	0	0	0	0	0	0	0	0	0	0	0	0	0	0
	5	1	0	0	1	0	0	0	0	0	0	0	1	0	0	1	0	1	0	0	0

Supplementary Table S1. Continued.

Site	Ind.	Locus																			
		21	22	23	24	25	26	27	28	29	30	31	32	33	34	35	36	37	38	39	40
	12	1	1	0	1	1	0	1	0	0	1	0	1	0	1	1	1	0	1	1	1
	13	1	1	0	1	1	0	1	0	1	1	0	1	0	1	1	1	0	0	1	1
	14	0	0	0	1	1	0	0	0	0	0	0	1	0	0	0	0	0	0	0	1
	15	0	0	0	1	1	0	1	1	1	1	0	1	0	1	1	0	0	1	1	1
	16	1	1	0	1	1	0	1	0	1	0	0	1	0	1	1	1	0	1	0	1
	17	1	1	0	0	1	0	1	0	0	1	0	1	0	1	0	0	0	1	0	1
	18	0	0	0	0	1	0	1	0	1	1	0	1	0	0	0	0	0	1	0	0
	19	1	1	0	0	1	0	1	1	0	1	1	1	0	1	1	1	0	1	1	1
	20	1	1	0	1	1	0	1	1	0	0	0	1	0	0	1	0	0	0	1	1
	21	0	0	0	1	1	1	0	0	0	0	0	0	0	0	0	1	0	0	0	1
VLA	22	1	0	0	1	1	0	0	1	0	0	1	1	0	0	1	1	0	1	1	1
	23	1	1	0	1	1	0	1	1	1	0	1	1	0	0	0	0	0	1	1	1
	24	1	1	0	0	1	0	1	0	1	1	0	1	0	1	1	1	0	1	1	1
	25	1	1	0	0	1	0	1	1	0	0	0	1	0	1	1	0	0	1	1	1
	26	1	1	0	1	1	0	1	0	0	1	0	1	0	1	0	1	0	0	1	1
	27	1	1	0	1	1	0	1	0	0	0	0	1	0	0	0	0	0	0	1	1
	28	1	1	0	1	1	0	1	0	1	0	0	1	0	0	1	1	0	0	1	1
	29	1	1	0	1	1	0	1	0	0	0	0	1	0	1	0	0	0	0	1	1
	30	1	1	0	0	1	0	1	0	1	1	1	1	0	1	1	0	0	1	0	1
	31	0	1	0	0	1	0	1	0	1	1	1	1	0	1	0	0	0	1	1	1
	32	0	0	0	0	0	0	1	0	0	0	0	1	0	0	0	0	0	0	1	1
	1	0	0	0	1	1	0	0	0	1	0	1	1	0	0	0	1	0	0	1	0
	2	0	0	0	0	0	0	0	0	0	0	0	0	0	0	0	0	0	0	1	0
CAÑ	3	0	0	0	0	0	0	0	0	0	0	0	0	0	0	0	0	0	0	0	0
	4	1	0	0	0	0	0	0	0	0	0	0	0	0	0	0	0	0	0	0	0
	5	1	0	1	1	0	0	1	0	1	0	0	0	0	0	0	0	0	0	1	0

Supplementary Table S1. Continued.

Site	Ind.	Locus																			
		41	42	43	44	45	46	47	48	49	50	51	52	53	54	55	56	57	58	59	60
	12	0	0	0	1	1	0	0	0	1	0	0	0	1	1	0	1	0	0	0	0
	13	1	0	0	1	0	0	0	0	1	0	0	1	1	1	1	1	1	0	0	0
	14	0	0	0	0	0	1	0	0	0	0	1	0	1	0	0	1	0	0	1	0
	15	1	0	0	1	0	0	0	1	1	0	1	1	1	1	0	1	1	0	0	0
	16	1	0	0	1	1	1	0	0	1	0	0	1	1	0	1	1	0	0	0	0
	17	0	0	1	1	1	1	1	0	1	0	1	1	0	0	0	1	0	0	0	0
	18	1	0	0	0	0	0	0	0	1	0	1	0	0	0	0	1	0	0	0	0
	19	1	0	0	1	0	0	0	0	1	0	0	1	1	1	0	1	1	0	0	0
	20	0	0	0	1	0	0	0	0	1	0	0	1	1	1	0	1	0	0	0	0
	21	0	0	0	1	1	0	0	0	1	0	1	1	0	0	0	1	0	0	0	0
VLA	22	1	0	1	1	0	1	0	1	0	0	1	0	0	0	1	1	1	0	0	0
	23	1	0	0	0	0	1	0	1	0	0	0	1	0	0	0	0	0	0	1	0
	24	0	0	0	1	1	1	0	0	1	0	0	1	1	1	0	1	0	0	0	0
	25	1	0	0	1	0	1	0	1	0	0	0	1	1	1	1	1	0	0	0	0
	26	1	0	0	1	0	1	0	1	1	0	1	1	0	1	0	1	1	0	0	0
	27	0	0	0	1	0	0	0	1	1	0	0	1	1	1	0	1	0	0	0	0
	28	1	0	0	1	0	0	1	0	1	0	0	1	1	0	1	1	1	0	1	0
	29	0	0	0	1	0	1	0	0	1	0	0	1	1	1	0	1	1	0	0	0
	30	1	0	1	1	1	1	0	0	1	0	1	0	0	1	1	1	1	0	0	0
	31	0	0	1	1	0	1	0	0	1	0	0	1	1	1	0	1	1	1	0	0
	32	0	0	0	1	1	1	0	0	1	0	0	1	1	1	0	1	0	0	0	0
	1	0	0	0	0	1	1	1	1	1	1	0	1	0	0	0	0	1	0	0	1
	2	0	0	0	0	0	0	0	0	0	0	0	0	0	0	0	0	0	0	0	1
CAÑ	3	0	0	0	0	0	0	0	0	0	0	0	1	0	0	0	0	0	0	0	1
	4	0	0	0	0	0	0	0	0	0	0	0	0	0	0	0	0	0	0	0	1
	5	0	0	1	1	0	0	1	0	1	0	0	0	0	0	1	0	0	1	0	1

Supplementary Table S1. Continued.

Site	Ind.	Locus																			
		61	62	63	64	65	66	67	68	69	70	71	72	73	74	75	76	77	78	79	80
	12	0	1	1	0	0	1	0	0	0	0	1	1	1	0	0	0	0	0	1	0
	13	0	1	1	0	0	1	0	0	0	0	1	1	1	0	1	0	0	0	0	0
	14	0	1	1	0	1	1	0	0	1	0	0	0	0	0	1	0	0	0	1	1
	15	0	1	1	0	1	1	0	0	0	0	1	1	1	0	0	0	0	0	1	0
	16	0	1	1	0	1	1	0	0	0	0	1	1	1	0	1	0	0	0	1	0
	17	0	0	1	0	1	1	0	0	0	0	1	1	1	0	1	1	0	0	0	0
	18	0	0	0	1	0	1	0	1	0	0	0	1	0	0	0	0	0	0	0	0
	19	0	1	0	0	1	1	0	0	0	0	1	1	1	0	0	0	0	0	0	0
	20	0	1	0	0	1	1	0	0	1	0	1	1	1	0	0	0	0	0	0	0
	21	0	1	1	0	1	1	0	0	0	0	0	0	0	0	0	0	0	0	1	0
VLA	22	0	0	1	1	1	1	0	0	0	0	0	1	1	0	0	0	0	0	1	1
	23	0	0	1	0	0	1	0	0	0	0	0	0	1	0	0	0	0	0	0	0
	24	0	1	1	0	1	1	0	0	0	0	1	1	1	0	1	0	0	0	1	0
	25	0	1	1	0	0	1	0	0	0	0	1	1	1	0	1	0	0	0	1	0
	26	0	1	1	0	0	1	0	0	0	0	1	1	1	0	0	0	0	0	1	0
	27	0	1	1	0	1	1	0	0	0	0	1	1	1	0	1	0	0	0	0	0
	28	0	1	1	0	0	1	0	0	0	0	1	1	1	0	1	0	0	0	1	0
	29	0	1	1	0	1	1	0	0	0	0	1	1	1	0	0	0	0	0	1	1
	30	0	1	1	0	0	1	0	0	0	1	1	1	0	0	1	0	0	0	1	1
	31	0	1	1	1	1	1	0	0	0	0	1	1	1	0	0	0	0	0	1	1
	32	0	1	0	0	1	1	0	0	0	0	1	0	0	0	1	0	0	0	1	0
	1	1	1	0	0	0	0	1	0	0	0	1	1	0	1	0	0	1	1	1	1
	2	0	0	0	0	1	1	0	1	0	0	1	0	1	0	0	0	0	0	0	0
CAÑ	3	0	0	0	0	1	1	0	0	0	0	1	0	0	1	0	0	0	0	0	0
	4	0	0	0	0	0	1	1	0	0	0	1	1	0	0	0	0	0	0	0	1
	5	0	0	0	0	1	1	1	1	0	0	1	0	1	1	0	0	0	0	0	0

Supplementary Table S1. Continued.

Site	Ind.	Locus																			
		81	82	83	84	85	86	87	88	89	90	91	92	93	94	95	96	97	98	99	100
	12	0	0	1	0	0	0	0	0	0	0	1	1	0	0	1	1	0	1	0	1
	13	1	0	1	0	0	0	0	0	1	0	1	1	0	1	1	1	1	1	0	1
	14	0	0	0	0	0	0	0	1	1	0	0	1	0	0	1	1	1	0	0	0
	15	1	0	0	1	1	0	0	1	1	0	1	1	0	1	1	1	1	1	1	0
	16	0	0	0	1	0	0	0	1	0	0	0	1	1	1	1	1	0	1	1	0
	17	1	0	1	1	1	0	0	1	1	0	0	1	0	1	1	1	0	1	0	1
	18	1	0	0	0	0	0	0	0	0	0	0	1	0	0	0	0	1	1	0	0
	19	1	0	1	0	0	0	0	0	1	0	1	1	0	1	1	0	1	1	0	1
	20	1	0	1	1	0	0	0	0	1	0	1	1	0	1	1	1	1	1	1	1
	21	0	0	0	0	0	0	1	0	0	0	0	0	1	0	0	1	0	0	0	0
VLA	22	1	0	0	0	1	0	0	1	1	1	0	1	0	0	1	0	1	1	1	1
	23	0	0	0	0	0	0	0	1	0	0	0	1	0	0	0	1	0	0	0	0
	24	1	0	0	1	0	0	0	1	1	0	0	1	0	1	1	1	1	1	1	1
	25	1	0	1	1	0	0	0	0	1	0	1	1	0	1	1	1	1	1	1	1
	26	1	0	0	0	0	0	0	1	1	0	1	1	0	1	1	1	1	1	1	1
	27	1	0	1	0	0	0	0	1	1	0	1	1	1	1	1	0	1	1	1	1
	28	1	0	0	1	0	0	0	1	1	0	1	1	1	1	1	1	0	1	1	1
	29	1	0	0	1	0	1	0	0	1	0	1	1	0	1	1	1	1	1	1	1
	30	1	0	1	1	1	0	0	1	1	0	1	1	0	1	1	1	1	0	0	1
	31	1	0	1	0	0	0	0	0	1	0	1	1	1	1	1	1	0	1	1	1
	32	1	0	0	1	0	0	0	0	1	0	1	1	0	0	1	1	1	0	1	1
	1	0	0	1	0	0	0	1	0	0	0	0	0	0	0	1	0	0	1	0	0
	2	0	0	0	0	0	0	0	0	0	0	0	0	0	0	0	0	0	1	0	0
CAÑ	3	0	0	0	0	0	0	0	0	0	1	0	0	0	0	0	0	0	0	0	0
	4	0	0	0	0	0	0	0	1	0	1	0	0	0	0	0	0	0	0	0	0
	5	1	0	0	1	0	0	0	0	0	0	1	0	0	1	0	0	0	0	0	0

Supplementary Table S1. Continued.

Site	Ind.	Locus																			
		101	102	103	104	105	106	107	108	109	110	111	112	113	114	115	116	117	118	119	120
	12	1	1	0	0	0	0	1	0	1	0	0	0	0	0	1	0	0	1	0	1
	13	1	1	0	1	0	1	0	1	1	1	0	0	0	1	1	1	1	1	0	1
	14	0	1	0	0	1	0	0	1	1	0	0	0	1	0	0	1	1	1	1	1
	15	1	1	0	1	0	0	0	1	1	0	0	0	1	0	0	1	1	1	0	1
	16	0	1	0	1	0	0	1	0	1	0	0	0	1	1	1	1	1	1	0	1
	17	1	1	0	0	0	0	1	1	0	0	0	0	1	0	1	1	1	1	1	1
	18	0	0	0	0	0	1	1	1	0	0	0	0	0	1	0	1	0	1	1	1
	19	1	1	0	1	0	1	1	0	1	0	0	0	1	0	1	1	1	1	1	1
	20	1	1	0	1	0	0	0	0	1	1	0	0	0	0	1	1	1	1	0	1
	21	0	0	0	1	0	0	1	0	0	0	0	0	1	0	1	1	1	1	1	1
VLA	22	0	1	0	1	0	1	1	0	1	1	0	0	1	1	1	0	0	1	0	1
	23	1	0	0	0	0	1	0	1	1	1	0	0	1	0	0	1	1	0	0	1
	24	1	1	0	1	1	0	1	0	1	1	0	0	1	1	1	0	1	1	0	1
	25	0	1	0	1	0	1	1	0	1	1	0	0	0	1	1	1	1	1	0	1
	26	1	1	0	1	0	1	1	0	1	0	1	0	1	0	1	1	0	1	1	1
	27	0	1	0	1	0	1	0	0	1	1	0	0	1	1	1	1	0	1	0	1
	28	0	1	0	1	1	1	1	1	1	1	0	0	1	1	1	1	0	1	1	1
	29	0	1	0	1	0	1	1	0	1	1	0	0	0	1	1	0	0	1	1	1
	30	0	1	0	1	0	1	1	0	0	0	0	0	0	0	1	1	1	1	0	1
	31	1	1	0	1	0	0	0	0	0	1	0	0	0	0	1	0	0	1	0	1
	32	1	1	0	1	0	1	0	0	1	0	0	0	0	0	0	1	0	1	0	1
	1	0	0	0	0	0	0	0	0	0	0	0	0	0	0	1	0	1	1	1	0
	2	0	0	0	0	0	0	0	0	0	0	0	0	0	0	0	0	0	0	0	0
CAÑ	3	0	0	0	0	0	0	0	0	0	0	0	0	0	0	0	0	0	0	0	0
	4	0	0	0	0	0	0	0	0	0	0	0	0	0	0	0	0	0	0	0	0
	5	0	0	0	0	0	0	0	0	0	0	0	0	1	0	0	0	1	1	0	1

Supplementary Table S1. Continued.

Site	Ind.	Locus																			
		121	122	123	124	125	126	127	128	129	130	131	132	133	134	135	136	137	138	139	140
VLA	12	0	1	0	0	1	1	0	0	0	0	0	0	0	0	0	0	0	0	0	0
	13	0	1	0	0	1	1	0	1	0	1	1	0	0	1	0	0	0	1	1	0
	14	0	1	0	1	1	1	1	0	0	1	0	1	0	0	0	0	0	1	0	0
	15	0	1	0	0	1	1	0	0	0	1	0	0	0	0	0	1	0	1	1	0
	16	1	0	0	0	1	1	1	1	0	1	1	0	0	1	1	0	0	0	1	0
	17	0	1	0	0	1	0	1	1	0	0	1	0	0	1	1	0	0	1	1	0
	18	1	1	0	0	1	1	0	0	0	0	0	0	0	0	0	0	0	0	1	0
	19	0	0	0	0	1	1	1	0	0	1	1	0	0	0	1	0	0	1	1	0
	20	1	1	0	0	1	1	1	0	0	1	1	1	0	0	0	0	0	1	1	0
	21	0	1	0	0	0	1	0	0	0	0	0	1	0	1	0	0	0	0	0	0
	22	1	1	0	0	1	1	0	1	0	1	0	1	0	1	0	0	0	1	1	0
	23	0	1	0	0	0	0	0	0	0	0	0	0	0	0	1	0	0	0	0	0
	24	1	1	0	0	1	1	0	1	0	1	1	1	0	1	1	0	0	1	1	0
	25	1	0	0	0	1	1	0	1	0	1	1	1	1	0	0	0	0	1	1	0
	26	1	1	0	0	1	1	1	1	0	1	0	1	1	1	1	0	0	0	1	0
	27	1	1	0	0	1	1	0	1	0	1	1	1	0	1	1	0	0	1	1	0
	28	1	1	0	0	1	1	0	1	0	1	1	1	0	1	1	1	0	1	0	0
	29	1	1	0	1	1	1	0	1	0	0	1	0	1	1	0	0	0	1	1	0
	30	1	1	0	1	1	1	0	1	0	0	1	1	0	1	1	1	0	1	1	0
	31	1	1	0	0	1	1	0	1	0	0	1	1	1	1	1	0	0	1	1	0
	32	0	1	0	0	1	0	0	0	0	1	0	0	1	0	1	0	0	1	1	0
CAÑ	1	1	0	1	0	0	0	0	0	0	0	0	0	1	0	0	0	0	0	0	0
	2	0	0	0	0	0	0	0	0	0	0	0	0	0	0	0	0	0	0	0	0
	3	0	0	0	0	0	0	0	0	0	0	0	0	0	0	0	0	0	0	0	0
	4	0	0	0	0	0	0	0	0	0	0	0	0	0	0	0	0	0	0	0	0
	5	0	1	0	0	0	0	0	0	0	0	0	0	0	0	0	0	0	0	0	0

Supplementary Table S1. Continued.

Site	Ind.	Locus																			
		141	142	143	144	145	146	147	148	149	150	151	152	153	154	155	156	157	158	159	160
	12	0	0	1	0	0	0	0	0	1	0	1	1	0	0	1	0	1	0	0	0
	13	0	0	1	1	0	0	0	1	0	1	1	1	0	1	1	1	0	0	0	0
	14	0	0	0	0	0	0	0	1	0	0	1	1	0	0	1	1	1	1	1	0
	15	0	0	1	0	0	0	0	1	0	1	1	1	0	0	1	1	0	0	0	0
	16	0	0	0	1	0	0	0	1	0	1	1	1	1	1	1	0	0	0	0	1
	17	0	0	0	1	0	0	1	1	0	1	1	1	1	0	1	1	0	0	0	1
	18	0	0	0	0	0	0	0	0	0	0	1	1	1	0	1	1	1	1	0	0
	19	0	0	1	1	0	0	0	1	1	1	1	1	1	0	1	1	1	0	0	0
	20	0	0	1	1	0	0	0	1	0	1	1	1	0	1	0	1	0	0	0	1
	21	0	0	0	0	0	0	1	0	0	0	1	0	0	0	1	1	0	0	1	1
VLA	22	1	0	0	1	0	0	0	1	0	0	1	0	0	0	1	1	1	0	0	0
	23	0	0	0	0	0	0	0	0	1	0	1	1	0	0	1	1	0	0	0	0
	24	0	0	1	0	0	0	1	0	0	0	0	1	1	1	0	0	0	1	0	0
	25	0	0	1	0	0	0	1	0	0	0	1	1	1	1	0	0	0	1	0	1
	26	0	0	0	1	0	0	0	0	0	0	0	0	0	1	0	0	0	1	0	0
	27	0	0	1	1	0	0	1	0	0	0	0	0	0	0	0	0	0	1	0	0
	28	0	0	1	1	0	0	0	0	0	0	0	1	1	1	0	0	0	1	0	0
	29	1	0	1	1	0	0	1	0	0	0	0	1	0	1	0	0	0	1	1	1
	30	0	0	1	1	0	0	0	0	0	0	0	0	1	1	1	1	1	1	1	0
	31	0	0	1	0	1	0	1	0	0	0	0	1	1	1	1	0	0	1	0	0
	32	0	0	1	0	0	0	0	0	1	1	0	0	0	0	0	0	0	0	0	0
	1	0	0	0	0	0	0	0	0	0	0	0	1	0	0	1	0	0	1	0	0
	2	0	0	0	0	0	0	0	0	0	0	0	0	0	0	0	0	0	0	0	0
CAÑ	3	0	0	0	0	0	0	0	0	0	0	0	1	0	0	0	0	0	0	0	1
	4	0	0	0	0	0	0	0	0	0	1	1	1	0	1	0	0	0	0	0	1
	5	0	0	0	0	0	0	0	0	0	0	1	1	0	0	0	0	0	0	0	1

Supplementary Table S1. Continued.

Site	Ind.	Locus																			
		161	162	163	164	165	166	167	168	169	170	171	172	173	174	175	176	177	178	179	180
	12	0	1	0	0	1	1	1	0	1	1	1	1	1	1	1	1	0	0	0	0
	13	1	1	1	0	1	1	1	1	1	1	1	1	0	1	1	1	0	0	0	0
	14	0	1	0	1	0	1	1	1	1	1	0	1	0	1	0	0	0	0	0	1
	15	0	1	0	1	1	1	1	0	1	1	0	1	1	1	1	1	0	0	1	0
	16	1	1	0	0	1	1	1	1	1	1	0	1	1	1	1	1	0	0	0	1
	17	1	1	0	0	1	1	1	0	0	0	0	1	1	1	1	0	0	0	1	1
	18	1	1	0	1	1	1	0	1	1	1	0	1	0	0	1	1	1	0	0	1
	19	1	1	1	0	1	1	1	0	1	1	0	1	1	1	0	1	0	0	0	0
	20	1	1	1	1	1	1	1	0	1	1	1	1	1	1	1	1	0	0	0	0
	21	1	1	0	0	0	1	1	0	0	0	0	0	0	0	0	0	0	0	0	0
VLA	22	1	0	0	0	0	0	1	1	1	0	0	1	1	1	1	1	0	0	1	0
	23	0	1	1	0	1	0	1	1	0	1	1	0	1	0	1	0	0	0	0	1
	24	0	0	0	1	1	0	0	1	1	0	0	1	1	0	0	0	0	0	0	0
	25	0	0	1	0	1	0	0	1	1	0	0	1	0	0	0	1	0	1	1	0
	26	0	0	1	1	1	0	0	1	0	0	0	1	0	0	0	1	0	0	0	0
	27	0	0	0	0	0	0	0	0	0	0	0	0	0	0	0	0	0	0	0	0
	28	0	0	1	1	0	0	0	0	0	0	0	0	0	0	0	0	0	0	0	0
	29	0	0	1	1	1	0	0	0	0	0	0	0	0	0	0	1	0	0	0	0
	30	0	0	1	1	1	0	0	1	0	0	0	0	0	0	0	1	0	0	0	0
	31	0	0	1	1	1	0	0	0	0	0	0	0	0	0	0	1	0	0	0	0
	32	0	1	1	1	1	1	0	1	1	0	0	0	0	0	0	0	0	0	0	0
	1	1	1	1	0	0	1	0	1	1	0	0	1	0	0	0	0	1	0	0	1
	2	1	1	0	0	0	0	1	0	0	1	0	0	0	0	0	0	0	0	0	1
CAÑ	3	0	0	0	0	0	1	1	0	1	0	0	0	0	1	0	0	0	0	0	0
	4	1	1	0	0	1	0	1	1	1	1	0	1	1	0	0	0	0	0	0	0
	5	1	0	0	0	0	1	1	0	0	0	0	0	1	0	0	0	0	0	0	0

Supplementary Table S1. Continued.

Site	Ind.	Locus																			
		181	182	183	184	185	186	187	188	189	190	191	192	193	194	195	196	197	198	199	200
	12	0	1	0	0	1	0	1	1	1	0	1	1	0	1	1	0	1	1	1	0
	13	1	1	0	0	0	1	1	1	1	1	1	1	1	1	1	0	1	1	1	0
	14	0	1	1	0	1	0	0	0	1	0	0	0	0	0	1	0	1	0	0	0
	15	1	1	0	0	1	0	0	1	0	0	1	0	1	1	1	0	1	1	1	1
	16	1	1	0	1	1	1	0	1	1	0	0	1	0	1	1	0	0	1	1	1
	17	1	1	0	0	1	0	0	1	0	0	0	1	0	1	1	1	1	1	0	0
	18	0	1	0	0	1	0	1	1	0	0	0	1	1	1	0	1	1	0	0	0
	19	1	1	0	1	0	0	1	1	0	1	1	1	1	1	1	0	0	1	0	1
	20	1	1	0	0	1	1	1	1	1	0	1	1	1	1	1	0	1	1	1	1
	21	1	1	0	1	1	0	1	0	0	0	0	0	0	1	1	1	0	0	1	0
VLA	22	1	1	1	0	1	1	1	1	1	1	1	1	0	1	1	0	1	1	0	1
	23	1	0	1	0	1	1	1	0	1	0	0	0	0	1	1	1	1	0	1	0
	24	0	0	0	0	1	0	0	0	0	1	1	0	0	0	0	0	1	1	0	0
	25	1	0	0	1	1	0	0	0	0	1	1	0	1	1	1	0	1	1	1	0
	26	0	0	0	0	1	0	0	0	0	1	1	0	0	1	0	0	0	1	0	0
	27	0	0	0	0	1	0	0	0	0	1	1	0	0	0	0	0	0	1	0	0
	28	0	0	0	1	1	0	0	0	0	1	1	0	0	1	0	0	0	1	0	0
	29	0	0	0	1	1	0	0	0	0	1	1	0	1	1	0	0	1	1	0	0
	30	0	0	0	0	1	0	0	0	0	1	1	0	1	1	0	0	1	1	0	1
	31	0	0	0	0	1	0	0	0	0	1	1	0	0	1	0	0	1	1	0	0
	32	0	1	0	0	1	0	0	1	0	0	0	1	1	1	1	1	1	0	0	0
	1	1	0	0	1	1	0	0	1	0	1	1	0	0	1	0	0	1	1	1	0
	2	1	1	0	0	1	1	0	0	0	0	0	0	1	1	1	0	0	1	0	0
CAÑ	3	0	1	0	0	1	0	0	1	0	0	0	1	1	0	1	0	0	1	0	0
	4	1	1	0	0	1	1	0	1	0	0	0	0	0	1	0	0	0	1	0	0
	5	1	1	0	0	0	0	0	1	0	0	0	0	0	1	0	0	0	0	0	0

Supplementary Table S1. Continued.

Site	Ind.	Locus																			
		201	202	203	204	205	206	207	208	209	210	211	212	213	214	215	216	217	218	219	220
	12	0	1	0	1	1	0	0	1	0	0	1	0	0	0	0	1	1	0	1	1
	13	0	1	1	1	1	0	0	1	0	0	1	1	0	0	1	1	1	0	1	1
	14	0	1	0	1	0	1	0	0	0	0	1	1	0	0	1	1	1	0	0	1
	15	1	1	0	1	1	0	0	1	0	0	0	1	0	0	1	1	0	1	1	1
	16	0	1	0	1	1	1	0	1	0	0	1	1	0	0	1	1	0	0	1	1
	17	0	0	0	1	1	0	0	1	1	0	1	0	0	0	1	0	0	0	1	1
	18	1	0	0	0	0	0	1	0	0	1	0	0	0	0	1	1	0	0	1	1
	19	0	1	0	1	1	0	0	0	0	0	1	1	0	0	1	1	0	1	1	1
	20	1	1	1	1	1	0	0	1	0	0	0	1	0	0	1	1	0	1	1	1
	21	1	1	1	1	1	0	0	0	0	1	1	1	0	0	1	1	1	0	1	1
VLA	22	1	1	0	1	1	1	0	1	1	0	0	0	0	0	1	1	1	0	1	1
	23	0	1	0	0	1	0	1	0	0	0	0	0	0	1	1	0	1	0	1	1
	24	1	0	1	0	0	0	1	1	0	0	1	0	0	1	0	0	1	0	0	1
	25	1	0	1	0	0	1	1	1	0	0	1	0	0	0	0	1	1	0	1	1
	26	0	0	0	0	0	0	1	0	0	0	1	0	0	0	0	0	0	0	1	0
	27	0	0	0	0	0	0	1	0	0	0	1	0	0	0	0	0	0	0	0	1
	28	0	0	0	0	0	0	1	0	0	0	1	0	0	0	0	0	0	0	0	1
	29	0	0	0	0	0	0	1	0	0	0	1	0	0	0	0	1	1	0	0	1
	30	0	0	0	0	0	0	1	0	0	0	1	0	0	0	0	1	0	0	0	1
	31	0	0	0	0	0	0	1	0	0	0	1	0	0	0	1	0	0	0	0	1
	32	0	0	0	0	1	0	0	0	0	0	1	0	0	0	1	1	1	1	1	1
	1	1	0	1	1	1	0	1	1	0	0	0	0	0	0	0	1	0	1	0	1
	2	0	1	0	0	0	0	0	1	0	0	0	0	0	0	1	1	0	1	1	1
CAÑ	3	0	1	0	1	1	0	0	0	0	0	1	0	0	0	0	0	1	1	1	1
	4	0	1	0	1	0	0	0	0	0	0	1	1	0	0	1	0	0	0	1	1
	5	0	0	0	0	1	0	0	0	0	0	0	0	0	0	0	1	0	0	1	1

Supplementary Table S1. Continued.

Site	Ind.	Locus																			
		221	222	223	224	225	226	227	228	229	230	231	232	233	234	235	236	237	238	239	240
	12	1	1	0	0	1	0	0	1	1	1	0	1	1	1	0	1	1	1	0	1
	13	1	1	1	1	1	0	0	1	1	1	0	0	1	1	0	1	1	1	0	1
	14	1	1	0	0	1	0	0	0	0	1	0	1	0	1	0	1	1	0	0	1
	15	1	1	0	1	1	0	0	1	1	1	1	1	1	1	0	1	1	1	0	1
	16	1	1	0	1	0	0	0	1	1	1	1	0	1	1	0	0	1	1	0	1
	17	1	0	0	1	0	0	0	1	0	1	0	1	0	1	1	1	1	1	0	1
	18	1	1	0	1	1	0	0	1	0	1	0	1	0	0	0	1	0	1	1	1
	19	1	1	1	1	1	0	0	1	1	1	0	0	1	0	0	1	1	1	1	1
	20	1	1	0	1	1	0	0	1	1	1	1	0	0	1	0	1	1	1	1	1
	21	1	1	0	0	1	0	0	1	1	1	0	0	0	0	0	0	1	0	0	1
VLA	22	1	1	0	0	0	0	0	1	1	1	1	0	0	0	0	0	1	1	0	1
	23	1	1	0	0	0	0	0	1	1	1	0	1	0	0	0	1	1	0	0	1
	24	1	0	0	1	0	0	0	0	1	0	1	0	1	0	1	0	1	1	1	0
	25	1	0	1	1	0	1	0	0	1	0	1	0	0	0	1	0	0	1	1	0
	26	1	0	0	1	0	0	0	0	1	0	1	0	0	0	1	0	0	1	1	0
	27	1	0	0	1	0	0	0	0	0	1	0	0	0	0	0	0	0	1	1	0
	28	1	0	0	1	0	0	0	0	0	1	0	0	0	0	0	0	0	1	0	0
	29	1	0	0	0	0	0	0	0	0	0	0	0	0	0	0	0	0	1	1	0
	30	1	0	1	1	0	0	0	0	1	0	1	0	1	0	1	0	0	1	0	0
	31	1	0	1	1	0	0	0	0	0	1	1	0	0	0	1	0	0	0	0	0
	32	1	1	0	0	0	0	0	1	0	1	0	0	0	1	0	1	1	0	0	1
	1	1	0	1	1	1	0	0	0	1	0	0	0	0	0	0	0	1	0	0	1
	2	1	1	0	0	0	0	0	1	0	0	0	0	0	1	0	1	0	0	1	1
CAÑ	3	0	1	0	1	0	0	0	1	0	1	0	0	0	0	0	1	1	0	1	1
	4	0	0	0	1	1	0	0	0	0	1	0	0	0	1	0	0	1	1	1	1
	5	1	0	0	1	0	0	0	0	1	0	0	0	0	0	0	0	0	0	0	1

Supplementary Table S1. Continued.

Site	Ind.	Locus																			
		241	242	243	244	245	246	247	248	249	250	251	252	253	254	255	256	257	258	259	260
	12	0	1	1	0	0	1	1	0	1	1	1	0	0	1	1	1	0	1	1	0
	13	1	1	0	0	1	1	1	0	1	1	1	0	1	1	1	1	0	1	1	0
	14	1	0	0	0	0	1	1	0	1	0	1	0	1	1	1	0	0	1	1	0
	15	0	1	1	0	1	1	1	0	1	0	1	0	0	1	1	0	0	1	1	0
	16	0	1	0	1	0	0	1	0	1	1	1	0	0	1	1	1	0	1	1	0
	17	0	0	0	0	0	0	1	0	0	0	1	0	0	1	1	1	1	1	0	0
	18	0	1	1	0	1	1	1	0	0	0	0	0	0	1	1	1	0	1	1	0
	19	0	1	0	0	1	0	1	1	1	1	1	0	0	1	1	1	0	0	1	0
	20	1	1	0	0	1	1	1	0	1	1	1	0	0	1	1	1	0	0	1	0
	21	0	0	0	1	0	1	1	1	1	0	1	0	0	1	1	1	0	1	1	0
VLA	22	0	1	0	1	1	1	0	1	1	1	1	0	0	1	1	0	0	1	0	0
	23	0	0	0	0	1	1	1	1	0	0	0	0	0	1	1	0	0	1	1	0
	24	1	1	1	0	0	0	0	0	0	1	0	1	0	1	0	0	1	0	1	0
	25	1	1	1	0	0	1	0	1	0	1	0	1	0	1	0	0	0	0	1	0
	26	1	0	0	0	0	1	0	1	0	0	0	1	0	1	1	0	1	1	1	0
	27	1	0	0	0	0	0	0	0	0	0	0	1	0	1	0	0	0	1	1	0
	28	0	0	0	0	0	0	0	0	0	0	0	0	0	0	0	0	0	1	1	0
	29	0	0	0	0	0	1	0	0	0	0	0	0	0	0	0	0	0	1	1	0
	30	0	0	1	0	0	1	0	0	0	0	0	1	0	0	0	0	1	0	1	0
	31	0	1	0	0	0	0	0	0	0	0	0	0	0	1	0	0	1	1	1	0
	32	1	1	0	0	1	1	1	0	1	0	1	0	0	0	1	1	0	1	1	0
	1	1	0	0	1	0	0	0	1	0	1	1	1	0	0	0	0	0	1	1	0
	2	1	1	0	0	0	1	1	0	0	1	0	0	0	1	0	1	0	1	1	0
CAÑ	3	0	1	0	1	1	0	1	1	0	1	1	0	0	1	1	1	0	1	1	0
	4	0	1	0	0	0	1	1	1	0	1	1	0	0	1	1	1	0	1	1	0
	5	0	0	0	0	1	1	1	0	0	0	1	0	0	1	1	1	0	1	1	0

Supplementary Table S1. Continued.

Site	Ind.	Locus 261	262	263	264	265	266	267	268
	12	1	0	0	1	0	0	1	1
	13	1	0	0	1	0	0	1	1
	14	1	0	0	1	0	0	0	1
	15	1	0	0	1	0	0	1	1
	16	1	0	0	1	0	0	0	1
	17	1	0	0	1	0	0	0	1
	18	1	0	0	1	0	0	1	1
	19	1	0	0	1	0	0	0	1
	20	1	0	0	1	0	0	1	1
	21	0	0	0	1	0	0	0	1
VLA	22	0	0	0	1	0	0	1	1
	23	1	0	0	1	0	1	1	1
	24	0	0	0	1	1	0	1	1
	25	0	0	0	0	1	0	1	1
	26	0	0	0	1	1	0	0	1
	27	0	0	0	1	1	0	0	1
	28	0	0	0	0	1	0	0	0
	29	0	0	0	1	1	0	0	0
	30	0	0	0	0	1	0	0	1
	31	0	0	0	1	1	0	1	1
	32	1	0	0	1	0	0	0	1
	1	1	0	0	0	1	0	1	0
	2	1	0	0	1	0	0	0	1
CAÑ	3	1	0	0	1	0	0	1	1
	4	1	0	0	1	0	0	0	1
	5	1	0	0	1	0	0	0	1

Supplementary Table S1. Continued.

Site	Ind.	Locus																			
		1	2	3	4	5	6	7	8	9	10	11	12	13	14	15	16	17	18	19	20
CAÑ	6	0	0	0	0	0	0	0	0	0	0	0	0	0	0	0	0	0	0	0	0
	7	0	0	0	0	0	0	0	0	0	0	0	0	0	0	0	0	0	0	0	0
	8	0	0	0	0	0	0	0	0	0	0	0	0	0	0	0	0	1	0	0	0
	9	0	0	0	0	0	0	0	0	0	0	0	0	0	0	0	0	0	0	0	0
	10	0	0	0	0	0	0	0	0	0	0	0	0	0	0	0	0	0	0	0	0
	11	0	0	0	0	0	0	0	0	0	0	0	0	0	0	0	0	0	0	0	0
GIC	1	1	1	1	0	0	1	0	0	1	0	1	0	0	0	1	0	1	0	1	1
	2	1	1	1	0	1	1	0	0	1	0	1	1	0	0	1	0	1	0	1	1
	3	1	0	1	1	1	1	0	0	1	0	0	1	0	0	0	0	0	0	1	1
	4	1	0	0	0	1	1	0	0	1	0	0	0	0	0	1	0	1	0	1	1
	5	0	0	0	0	0	0	0	0	0	0	0	0	0	0	0	0	0	0	0	0
	6	0	0	0	0	0	1	0	0	0	0	0	1	0	0	1	0	0	0	0	0
ICM	1	1	1	0	1	1	1	0	0	1	0	1	0	0	1	1	0	1	0	1	1
	2	0	1	1	1	1	1	0	0	1	0	1	0	0	0	0	0	0	0	1	1
	3	1	0	0	0	0	1	0	0	1	0	1	0	0	0	0	0	1	0	1	0
	4	1	0	1	1	1	1	0	1	1	0	0	1	0	1	1	0	0	0	1	1
	5	1	1	1	0	1	1	0	0	1	0	1	0	0	1	1	0	1	0	1	0
	6	1	1	1	0	0	1	0	0	1	0	1	0	0	1	1	0	0	0	1	1
	7	1	1	0	0	1	1	0	0	0	0	0	1	0	0	0	0	1	0	1	0
	8	1	0	0	1	1	1	0	0	1	0	0	0	0	0	1	0	0	0	0	0
	9	0	0	0	0	0	0	1	0	0	1	0	0	1	1	0	1	0	0	1	0
	10	0	0	0	0	0	0	1	0	0	1	0	0	1	1	0	0	0	1	0	0
	11	0	0	0	0	0	0	1	0	0	0	0	0	1	1	0	1	0	0	0	0
	12	0	0	0	0	0	0	1	0	0	0	0	0	1	0	0	1	0	0	0	0
PNC	1	0	0	0	0	0	0	0	0	0	0	0	0	0	0	0	0	0	0	0	0
	2	0	0	0	0	0	0	0	0	0	1	0	0	0	0	0	1	0	1	1	0

Supplementary Table S1. Continued.

Site	Ind.	Locus																			
		21	22	23	24	25	26	27	28	29	30	31	32	33	34	35	36	37	38	39	40
CAÑ	6	0	0	0	0	0	0	0	0	0	0	0	0	0	0	0	0	0	0	1	0
	7	0	0	0	0	0	0	0	0	0	0	0	0	0	0	0	0	0	0	0	0
	8	0	0	0	0	0	0	0	0	0	0	0	0	0	0	0	0	0	0	1	0
	9	0	0	0	0	0	0	0	0	0	0	0	0	0	0	0	0	0	0	1	0
	10	0	0	0	0	0	0	0	0	0	0	1	0	0	0	0	0	0	0	0	0
	11	0	0	0	0	0	0	0	0	0	0	0	0	0	0	0	0	0	0	0	0
GIC	1	1	0	0	1	1	0	1	0	1	0	1	1	0	0	0	0	0	1	0	0
	2	1	1	0	1	1	0	0	0	1	0	1	1	0	1	0	1	0	0	0	0
	3	0	0	1	1	1	0	1	0	1	0	1	1	0	0	0	0	0	0	1	1
	4	0	0	1	1	1	0	1	0	1	0	1	1	1	0	0	1	1	0	0	0
	5	0	0	0	0	0	0	0	0	0	0	0	0	0	0	0	0	0	0	1	0
	6	0	0	0	1	0	0	0	0	1	0	0	0	0	0	0	0	0	0	0	0
ICM	1	1	0	0	0	1	0	1	0	1	0	0	1	0	0	0	0	0	0	1	1
	2	1	0	0	1	1	0	0	0	1	0	0	0	0	0	0	1	1	1	0	0
	3	0	0	0	0	1	0	0	0	1	0	1	1	0	0	0	0	0	0	1	0
	4	1	0	0	1	1	0	1	0	1	0	1	1	0	1	0	1	0	0	0	0
	5	1	0	0	1	1	0	0	0	1	0	1	1	0	0	0	0	0	0	1	0
	6	1	0	0	1	1	0	1	0	1	0	1	1	1	0	0	0	0	0	1	0
	7	0	0	0	1	1	0	0	0	0	0	0	0	0	0	0	0	0	0	0	0
	8	1	1	1	1	1	0	1	0	0	0	0	1	1	0	0	0	0	0	0	0
	9	0	0	0	0	0	1	0	0	0	0	1	0	0	0	1	0	0	0	0	0
	10	0	0	0	0	0	1	0	0	0	0	1	0	0	0	1	1	0	0	0	1
	11	0	0	0	0	0	1	0	0	0	0	1	0	0	0	1	0	0	0	0	1
	12	0	0	0	0	0	1	0	0	0	0	1	0	0	0	1	0	0	0	0	0
PNC	1	0	0	0	0	0	0	0	0	0	0	0	0	0	0	0	0	0	0	0	0
	2	0	0	0	0	1	0	0	0	1	0	1	0	1	0	0	1	0	0	0	0

Supplementary Table S1. Continued.

Site	Ind.	Locus																			
		41	42	43	44	45	46	47	48	49	50	51	52	53	54	55	56	57	58	59	60
CAÑ	6	0	0	0	0	0	0	0	0	0	0	0	0	0	0	0	0	0	0	0	1
	7	0	0	0	0	0	0	0	0	0	0	0	0	0	0	0	0	0	0	0	1
	8	0	0	0	0	0	0	0	0	0	0	0	0	0	0	0	0	0	0	0	1
	9	0	0	0	0	0	0	0	0	0	0	0	0	0	0	0	0	0	0	0	1
	10	0	0	0	0	0	0	0	0	0	0	0	0	0	0	0	0	0	0	0	1
	11	0	0	0	0	0	0	0	0	0	0	0	0	0	0	0	0	0	0	0	1
GIC	1	0	0	1	1	0	1	1	0	0	0	0	0	0	0	1	1	1	1	1	1
	2	0	1	0	0	1	1	1	0	0	0	0	1	0	1	0	1	0	0	0	1
	3	0	0	1	0	1	1	1	1	0	1	1	0	0	1	0	0	0	0	1	1
	4	0	0	0	1	0	1	0	1	0	0	1	0	0	0	1	1	0	0	1	1
	5	0	0	0	0	0	0	0	0	0	0	0	0	0	0	0	0	0	0	0	1
	6	0	1	1	1	0	0	0	0	1	0	0	0	0	0	1	0	0	0	0	1
ICM	1	0	1	1	0	1	1	1	0	0	0	0	0	0	0	1	0	0	1	0	1
	2	0	0	0	0	0	0	0	0	0	0	0	0	0	0	0	0	0	1	0	0
	3	0	0	1	0	0	1	0	1	0	0	0	0	0	0	0	0	0	0	0	1
	4	0	0	0	1	1	1	1	0	0	0	0	1	0	1	1	0	1	0	0	1
	5	0	0	0	1	1	1	1	1	1	1	0	0	0	0	0	1	1	1	1	1
	6	0	0	0	0	0	1	0	1	0	1	0	0	0	0	0	1	0	0	1	1
	7	0	0	0	0	0	0	0	0	1	1	0	0	0	0	0	0	0	0	0	1
	8	0	0	0	1	1	0	0	1	0	1	0	0	0	0	1	0	0	0	0	0
	9	1	0	1	1	0	0	0	0	0	1	0	0	0	0	1	1	0	1	1	0
	10	1	0	1	1	0	0	1	0	0	0	0	0	0	0	1	1	0	1	1	0
	11	1	1	1	1	0	0	0	0	0	0	0	0	0	0	1	1	0	1	1	0
	12	1	0	1	0	0	0	0	0	0	0	0	0	0	0	1	1	0	1	1	0
PNC	1	0	0	0	0	0	0	0	0	0	0	0	0	0	0	0	0	0	0	0	0
	2	0	0	0	0	1	0	0	0	0	1	0	0	0	0	0	0	0	0	1	0

Supplementary Table S1. Continued.

Site	Ind.	Locus																			
		61	62	63	64	65	66	67	68	69	70	71	72	73	74	75	76	77	78	79	80
CAÑ	6	0	0	0	0	1	1	1	0	0	0	0	1	0	0	0	0	0	0	0	0
	7	0	0	0	0	1	1	1	1	0	0	1	1	1	1	0	0	0	0	0	0
	8	0	0	0	0	0	1	1	1	0	0	1	0	1	0	1	0	0	0	0	1
	9	0	0	0	0	1	1	1	1	0	0	1	0	0	0	0	0	0	0	0	0
	10	0	0	0	0	0	1	1	1	0	0	1	0	0	0	0	0	0	0	0	0
	11	0	0	0	0	1	1	1	1	0	0	1	1	0	1	0	0	0	0	0	1
GIC	1	1	1	0	0	0	1	0	0	1	0	0	1	1	0	1	0	0	0	0	1
	2	0	1	0	0	0	0	1	1	0	0	0	1	0	0	0	0	1	0	0	1
	3	1	1	0	0	0	0	0	0	1	0	0	1	1	0	1	1	0	0	1	1
	4	1	1	0	0	1	1	0	0	1	0	0	1	0	0	0	0	1	0	0	1
	5	0	0	0	0	1	1	1	1	0	0	1	1	0	0	0	0	0	0	0	1
	6	0	0	0	0	1	0	0	0	0	0	1	1	1	0	0	0	0	0	1	0
ICM	1	0	1	0	0	0	1	1	0	0	1	1	1	1	0	0	1	1	1	1	1
	2	0	1	0	0	0	1	0	0	1	0	0	1	0	0	0	1	1	0	0	0
	3	1	1	0	0	0	0	0	1	1	0	1	1	0	0	0	0	1	0	0	0
	4	1	1	0	0	1	0	0	0	1	0	0	1	1	0	0	0	1	1	1	1
	5	1	1	0	0	0	1	0	0	0	0	1	1	0	1	0	0	0	1	1	1
	6	0	0	0	0	0	0	0	1	0	0	0	0	0	0	0	0	0	1	1	1
	7	0	0	0	0	0	1	0	0	0	0	0	1	0	0	0	0	0	0	0	0
	8	0	0	0	0	1	1	0	1	0	0	1	0	1	0	1	0	1	0	1	1
	9	0	0	0	1	0	0	0	0	0	0	0	0	0	1	1	0	0	0	0	0
	10	0	0	0	1	1	0	0	0	0	0	0	0	0	1	1	0	0	0	0	0
	11	0	0	0	1	1	0	0	0	0	0	0	0	0	1	0	0	0	0	0	0
	12	0	0	0	1	0	0	0	0	0	0	0	0	0	1	1	0	0	0	0	0
PNC	1	0	0	0	0	0	0	0	0	0	0	0	0	0	0	0	0	0	0	0	0
	2	1	1	0	0	0	0	0	0	0	0	0	0	0	1	0	0	0	0	0	0

Supplementary Table S1. Continued.

Site	Ind.	Locus																			
		81	82	83	84	85	86	87	88	89	90	91	92	93	94	95	96	97	98	99	100
CAÑ	6	0	0	0	0	0	0	0	0	0	0	0	0	0	0	0	0	0	0	0	0
	7	0	0	0	0	0	0	0	0	0	0	0	0	0	0	0	0	0	0	0	0
	8	0	0	0	0	0	0	0	0	0	0	0	0	0	0	0	0	0	0	0	0
	9	0	0	0	0	0	0	0	0	0	0	0	0	0	0	0	0	0	0	0	0
	10	0	0	0	0	0	0	0	0	0	0	0	0	0	0	0	0	0	0	0	0
	11	0	0	0	0	0	0	0	0	0	0	0	0	0	0	0	0	0	0	0	0
GIC	1	1	0	1	0	1	1	0	0	0	0	1	0	0	0	0	0	0	1	0	0
	2	0	0	1	1	0	0	0	0	0	0	0	0	0	0	1	1	0	1	0	0
	3	1	0	0	0	1	0	0	0	0	1	0	1	1	0	0	0	0	1	1	1
	4	1	0	0	0	0	0	1	0	0	0	0	0	0	0	1	0	0	1	0	0
	5	1	0	0	0	0	0	0	1	0	1	0	0	0	0	0	0	0	0	0	0
	6	1	0	0	0	0	0	0	0	0	0	0	0	0	1	0	0	0	0	0	0
ICM	1	0	0	1	1	1	0	1	0	0	0	1	1	1	0	1	0	1	1	0	1
	2	0	0	0	0	1	0	0	0	0	0	1	0	0	0	1	0	0	0	1	0
	3	0	0	1	1	1	1	0	0	0	1	1	1	0	0	1	0	0	0	0	0
	4	1	0	0	0	1	0	1	1	0	0	1	0	0	0	1	0	1	1	0	1
	5	1	0	0	0	0	0	1	1	0	0	0	1	0	0	1	0	0	1	0	1
	6	0	0	0	1	0	0	0	0	0	0	0	0	0	0	1	0	0	0	0	1
	7	0	0	0	0	0	0	0	0	0	0	0	0	0	0	0	0	0	0	0	0
	8	0	0	0	1	0	0	1	0	0	0	0	0	0	0	0	1	1	0	0	0
	9	0	0	0	0	0	1	0	0	0	0	1	0	0	1	0	0	1	0	0	1
	10	1	0	0	0	0	1	1	1	0	0	1	0	0	1	0	1	1	0	0	1
	11	0	0	0	1	0	0	0	0	0	0	1	0	0	1	0	0	1	0	0	1
	12	0	0	0	1	0	0	1	0	0	0	1	0	0	1	0	0	1	0	0	1
PNC	1	0	0	0	0	0	0	0	0	0	0	0	0	0	0	0	0	0	0	0	0
	2	0	0	0	0	1	0	1	1	0	0	0	0	0	0	1	0	0	1	0	1

Supplementary Table S1. Continued.

Site	Ind.	Locus																			
		101	102	103	104	105	106	107	108	109	110	111	112	113	114	115	116	117	118	119	120
CAÑ	6	0	0	0	0	0	0	0	0	0	0	0	0	0	0	0	1	0	0	0	0
	7	0	0	0	0	0	0	0	0	0	0	0	0	0	0	0	0	0	0	0	0
	8	0	0	0	0	0	0	0	0	0	0	0	0	0	0	0	0	0	1	0	0
	9	0	0	0	0	0	0	0	0	0	0	0	0	0	0	0	0	0	0	0	0
	10	0	0	0	0	0	0	0	0	0	0	0	0	0	0	0	0	0	0	1	0
	11	0	0	0	0	0	0	0	0	0	0	0	0	0	0	0	0	0	0	0	0
GIC	1	1	1	0	1	0	0	0	0	0	0	0	0	0	0	0	0	0	1	1	1
	2	1	0	0	1	0	0	0	0	0	0	0	0	0	0	0	0	0	0	0	0
	3	0	0	0	1	1	0	1	0	0	0	1	0	0	0	0	0	1	0	0	0
	4	1	1	0	1	1	0	0	1	0	0	0	0	1	1	0	1	0	1	0	1
	5	0	0	0	0	0	0	0	0	0	0	0	0	0	0	0	1	0	0	0	0
	6	0	0	0	0	0	0	0	0	0	0	0	0	0	0	0	0	0	1	0	1
ICM	1	1	0	0	1	1	0	1	0	0	0	1	0	1	1	1	1	0	0	0	0
	2	0	0	0	0	1	0	0	0	0	0	0	0	0	0	0	0	0	0	1	0
	3	0	0	0	0	0	0	0	0	0	0	0	0	1	1	0	1	0	1	0	1
	4	1	0	0	0	1	0	1	0	0	1	0	0	1	0	0	1	1	1	1	0
	5	0	0	0	0	0	0	1	0	0	0	0	0	0	1	0	0	0	0	1	0
	6	0	0	0	0	0	0	0	0	0	0	0	0	0	1	0	0	0	0	0	0
	7	0	0	0	0	0	0	0	0	0	0	0	0	0	0	0	0	0	0	0	0
	8	0	0	0	0	0	0	0	0	0	1	0	0	0	0	0	0	0	0	0	0
	9	0	1	0	1	0	1	0	0	0	0	0	1	1	0	0	0	0	0	1	1
	10	1	1	0	1	0	1	0	0	0	0	0	1	1	1	1	0	0	0	1	1
	11	1	1	0	1	0	1	0	0	0	0	0	0	1	0	0	0	0	0	1	1
	12	0	0	0	1	0	1	0	0	0	0	0	0	1	1	1	0	0	0	1	1
PNC	1	0	0	0	0	0	0	0	0	0	0	0	0	0	0	0	0	0	0	0	0
	2	1	1	0	0	0	0	1	0	0	0	0	1	0	1	0	0	0	0	0	1

Supplementary Table S1. Continued.

Site	Ind.	Locus																			
		121	122	123	124	125	126	127	128	129	130	131	132	133	134	135	136	137	138	139	140
CAÑ	6	0	0	0	0	0	0	0	0	0	0	0	0	0	0	0	0	0	0	0	0
	7	0	0	0	0	0	0	0	0	0	0	0	0	0	0	0	0	0	0	0	0
	8	0	0	0	0	0	0	0	0	0	0	0	0	0	0	0	0	0	0	0	0
	9	0	0	0	0	0	0	0	0	0	0	0	0	0	0	0	0	0	0	0	0
	10	0	0	0	0	0	0	0	0	0	0	0	0	0	0	0	0	0	0	0	0
	11	0	0	0	0	0	0	0	0	0	0	0	0	0	0	0	0	0	0	0	0
GIC	1	1	1	1	0	0	0	0	0	0	0	0	0	0	0	1	1	0	0	0	0
	2	1	1	1	0	0	1	0	0	0	0	0	0	1	1	0	0	0	0	0	1
	3	1	1	1	0	0	0	0	1	0	1	0	0	1	0	1	1	1	0	0	0
	4	1	1	1	0	0	0	0	0	0	0	0	1	0	0	0	0	0	0	0	0
	5	0	0	0	0	0	0	0	0	0	0	0	0	0	0	0	0	0	0	0	0
	6	1	1	0	0	0	0	0	0	0	0	0	0	1	0	0	0	0	0	0	0
ICM	1	1	1	1	0	1	0	0	0	0	0	0	0	1	0	0	1	1	0	0	1
	2	0	1	1	1	0	0	0	0	0	0	0	0	0	0	0	0	0	0	0	0
	3	1	0	1	0	0	0	0	0	0	0	0	0	0	0	0	1	1	0	0	1
	4	1	1	1	0	1	1	0	1	0	0	1	0	1	0	0	1	1	0	0	1
	5	1	0	1	0	0	0	0	1	0	0	0	0	1	0	0	0	0	0	0	0
	6	1	0	1	0	0	0	0	0	0	0	0	0	0	0	0	0	0	0	0	0
	7	0	1	1	0	0	0	0	0	1	1	0	0	0	0	0	0	0	0	0	0
	8	0	0	0	0	1	0	0	0	0	0	0	0	0	0	0	0	0	0	0	0
	9	0	1	0	0	0	0	0	0	0	0	0	0	0	0	1	0	0	0	0	0
	10	0	1	0	0	0	0	0	0	0	0	0	0	0	0	1	0	0	0	0	0
	11	0	1	0	0	0	0	0	0	0	0	0	0	0	0	1	0	0	0	0	0
	12	0	1	0	0	0	0	0	0	0	0	0	0	0	0	0	0	0	0	0	0
PNC	1	0	0	0	0	0	0	0	0	0	0	0	0	0	0	0	0	0	0	0	0
	2	0	0	1	0	0	0	0	0	0	0	0	0	0	0	1	0	0	0	0	0

Supplementary Table S1. Continued.

Site	Ind.	Locus 141	142	143	144	145	146	147	148	149	150	151	152	153	154	155	156	157	158	159	160
CAÑ	6	0	0	0	0	0	0	0	0	0	0	1	1	0	0	0	0	0	0	0	0
	7	0	0	0	0	0	0	0	0	0	0	1	1	0	0	0	0	0	1	0	1
	8	0	0	0	0	0	0	0	0	0	0	0	0	0	0	0	0	0	1	0	0
	9	0	0	0	0	0	0	0	0	0	0	0	0	0	0	0	0	0	1	0	0
	10	0	0	0	0	0	0	0	0	0	0	0	0	0	0	0	0	0	1	0	0
	11	0	0	0	0	0	0	0	0	0	0	0	0	0	0	0	0	0	0	0	0
GIC	1	0	0	0	0	0	1	1	0	0	0	1	0	1	0	0	0	0	1	0	0
	2	0	0	0	0	0	0	1	0	1	0	0	1	0	1	0	0	0	1	0	0
	3	0	0	0	0	0	0	0	0	0	0	1	1	1	1	0	0	1	1	1	1
	4	0	0	0	0	0	1	1	1	1	0	1	1	0	0	1	1	0	1	1	0
	5	0	0	0	0	0	0	0	0	1	0	1	1	0	0	0	0	0	0	0	1
	6	0	0	0	0	0	0	0	0	0	0	1	1	0	0	0	0	1	0	0	1
ICM	1	1	0	1	0	0	0	0	1	0	1	1	0	0	0	1	0	1	0	0	1
	2	0	0	0	0	1	0	0	0	0	0	0	1	0	1	1	1	0	0	1	0
	3	1	0	0	0	1	0	1	0	0	0	1	1	0	1	1	0	0	0	0	0
	4	1	0	1	0	1	0	1	1	1	1	1	1	1	1	1	1	0	0	0	1
	5	0	0	0	0	1	0	0	0	1	1	1	0	1	1	0	0	0	1	0	1
	6	1	0	1	0	0	0	0	0	1	0	1	0	0	1	0	0	0	1	0	1
	7	0	0	0	0	0	0	0	0	0	0	1	0	0	0	0	1	0	1	0	1
	8	0	0	1	0	0	0	0	0	0	0	1	1	0	0	0	0	0	0	0	1
	9	0	1	0	0	0	0	0	0	0	0	1	1	0	0	0	0	0	0	0	0
	10	0	0	0	0	0	0	0	0	0	0	1	1	0	0	0	0	0	0	0	1
	11	0	0	0	0	0	0	0	1	0	0	1	1	0	0	0	0	0	0	0	1
	12	0	0	0	0	0	0	0	0	0	0	1	1	0	0	0	0	0	0	0	1
PNC	1	0	0	0	0	0	0	1	0	1	0	0	1	0	1	0	0	0	1	0	0
	2	0	1	0	0	0	0	0	0	0	0	1	1	1	1	0	0	1	1	1	1

Supplementary Table S1. Continued.

Site	Ind.	Locus 161	162	163	164	165	166	167	168	169	170	171	172	173	174	175	176	177	178	179	180
CAÑ	6	1	1	0	0	0	1	1	0	0	0	0	0	1	1	0	0	0	0	0	0
	7	0	1	0	0	0	1	0	0	1	0	0	0	1	0	0	0	0	0	0	0
	8	1	0	0	0	0	1	0	0	0	0	0	0	1	0	0	0	0	0	0	0
	9	0	0	0	0	0	1	0	0	0	0	0	0	0	0	0	0	0	0	0	0
	10	0	0	0	0	0	1	0	0	0	0	0	0	0	0	0	0	0	0	0	1
	11	0	0	0	0	0	1	0	0	0	0	0	0	0	0	0	0	0	0	0	0
GIC	1	1	0	1	0	0	0	0	1	1	0	0	1	0	0	1	1	0	0	0	0
	2	0	0	1	1	1	0	0	1	1	1	0	1	0	0	1	1	1	1	0	1
	3	0	0	1	0	1	1	0	1	0	0	0	0	0	0	0	0	0	1	1	1
	4	1	1	1	0	0	0	1	1	1	0	0	1	0	1	1	1	0	0	0	0
	5	1	0	0	1	0	0	1	0	0	0	0	0	1	0	0	1	0	0	0	0
	6	1	0	0	0	1	0	0	0	0	0	0	0	0	0	0	0	0	0	0	0
ICM	1	1	1	0	0	0	1	1	0	1	1	0	1	1	1	1	1	0	0	0	0
	2	1	0	0	0	0	1	0	0	0	0	0	0	1	1	0	0	0	0	0	1
	3	1	1	0	0	0	1	1	1	1	0	0	0	0	0	1	1	0	1	0	0
	4	1	0	1	0	0	1	1	0	1	1	1	0	1	0	1	1	0	0	0	1
	5	0	1	1	1	1	1	1	0	1	1	0	1	1	0	0	0	0	0	0	1
	6	1	0	1	0	1	0	0	0	1	1	0	0	0	0	0	0	0	0	0	0
	7	0	0	0	1	1	1	1	1	0	1	0	0	1	1	0	0	0	1	0	1
	8	1	1	0	0	0	0	1	0	1	0	0	0	1	0	0	0	0	0	0	0
	9	1	0	0	0	0	0	0	0	0	0	0	0	1	0	0	0	0	0	0	0
	10	1	1	0	0	0	1	1	0	1	0	0	0	0	1	1	0	0	0	0	0
	11	1	1	0	0	0	0	1	0	1	0	0	0	1	1	1	0	0	0	0	0
	12	1	1	0	0	0	1	1	0	0	0	0	1	1	1	0	0	0	0	0	1
PNC	1	0	0	1	1	1	0	0	1	1	1	0	1	0	0	1	1	1	1	0	1
	2	0	0	1	0	1	1	0	1	0	0	0	0	0	0	0	0	0	1	1	1

Supplementary Table S1. Continued.

Site	Ind.	Locus																			
		181	182	183	184	185	186	187	188	189	190	191	192	193	194	195	196	197	198	199	200
CAÑ	6	1	1	1	0	1	0	0	0	0	0	0	1	0	1	1	0	0	0	1	0
	7	1	1	0	0	1	0	0	0	0	0	0	0	0	1	1	0	0	0	1	0
	8	1	1	0	0	1	0	0	0	0	0	0	0	1	0	1	0	0	0	0	0
	9	1	1	0	0	1	0	0	0	0	0	0	0	0	1	1	0	0	0	0	0
	10	0	0	1	0	0	0	0	0	0	0	0	1	0	0	0	0	0	0	0	0
	11	0	1	1	0	1	0	0	0	0	0	0	0	1	1	1	0	0	0	0	0
GIC	1	1	1	0	0	1	0	0	0	0	0	0	0	1	1	0	0	1	1	1	0
	2	0	0	0	1	1	0	0	0	1	0	1	1	1	0	1	0	0	0	0	0
	3	0	0	1	1	1	1	0	0	0	0	0	1	0	0	0	0	0	0	0	0
	4	1	1	0	1	0	1	0	0	0	0	0	1	0	1	0	0	0	0	1	1
	5	1	1	0	0	0	0	0	1	0	1	1	1	0	1	1	0	0	1	1	0
	6	1	1	0	0	0	0	0	0	0	1	1	1	0	1	1	0	0	0	1	0
ICM	1	1	1	0	0	0	0	1	0	0	1	1	1	0	1	1	1	0	1	0	1
	2	1	0	0	0	1	0	0	1	0	0	0	0	0	0	0	0	0	0	0	0
	3	0	1	0	0	1	1	1	1	1	0	0	0	0	0	0	0	0	1	0	1
	4	1	1	1	0	0	0	1	1	1	1	0	1	0	0	0	0	1	1	0	1
	5	1	1	1	1	1	1	0	0	0	0	1	1	0	1	1	1	1	1	1	1
	6	0	1	1	1	0	0	0	0	0	0	1	1	0	1	0	1	0	1	1	1
	7	1	1	0	1	1	1	1	1	0	0	0	0	0	1	0	0	0	0	0	0
	8	1	1	0	0	0	0	0	0	0	0	0	0	0	1	1	0	0	0	0	0
	9	1	1	0	0	1	0	0	0	0	0	0	0	0	1	0	0	0	0	0	0
	10	1	1	0	0	1	0	0	1	0	0	0	1	0	1	1	0	0	0	0	0
	11	1	1	0	0	0	1	0	1	0	0	0	0	0	1	0	0	0	0	0	0
	12	1	1	0	0	1	0	0	1	0	0	0	0	0	1	1	0	0	0	0	0
PNC	1	0	0	0	1	1	0	0	0	1	0	1	1	1	0	1	0	0	0	0	0
	2	0	0	1	1	1	1	0	0	0	0	0	1	0	0	0	0	0	0	0	0

Supplementary Table S1. Continued.

Site	Ind.	Locus 201	202	203	204	205	206	207	208	209	210	211	212	213	214	215	216	217	218	219	220
CAÑ	6	0	0	0	0	1	0	0	0	0	0	1	0	0	0	1	1	0	1	0	1
	7	0	0	0	0	1	0	0	0	0	0	1	0	0	0	1	1	0	1	1	1
	8	0	0	0	0	0	0	0	0	0	0	0	0	0	0	1	1	1	0	0	1
	9	0	0	0	0	0	0	0	0	0	0	0	0	0	0	1	1	0	1	1	1
	10	0	0	0	0	0	0	0	0	0	0	1	0	0	0	1	1	0	0	0	1
	11	0	0	0	0	0	0	0	0	0	0	0	0	0	0	0	1	0	0	0	1
GIC	1	0	0	0	0	1	0	0	0	0	0	0	0	0	1	1	0	1	0	0	1
	2	0	0	0	0	0	0	1	0	0	0	0	1	0	0	1	0	0	0	0	1
	3	0	0	0	0	1	0	0	0	0	0	1	1	0	0	0	0	1	1	0	1
	4	1	1	0	0	0	0	1	1	0	0	0	0	0	1	1	0	1	0	0	1
	5	0	1	0	1	1	0	0	0	0	0	0	0	0	0	1	1	1	1	0	1
	6	0	0	0	0	1	1	0	0	0	0	0	0	0	0	0	1	0	0	1	1
ICM	1	0	0	1	0	1	0	0	0	0	0	0	1	0	0	1	0	0	0	1	1
	2	0	1	0	1	1	0	0	1	1	0	0	0	0	0	1	1	1	1	0	1
	3	0	1	0	0	0	0	0	1	0	0	0	0	0	1	1	0	1	0	0	1
	4	1	1	1	1	1	0	0	1	1	0	1	1	0	0	1	0	1	1	1	1
	5	1	1	1	0	1	1	0	0	0	0	1	0	0	0	1	0	1	1	1	1
	6	1	1	1	0	1	0	0	0	0	0	0	0	0	0	0	0	0	0	1	1
	7	1	0	0	0	0	0	0	0	0	0	0	0	0	0	0	1	0	0	0	1
	8	0	0	0	0	1	0	0	0	0	0	0	0	0	0	1	1	0	0	1	1
	9	0	0	0	0	0	0	0	0	0	0	0	0	0	0	1	0	0	0	1	1
	10	0	1	0	0	1	0	0	1	0	0	0	0	0	0	1	1	0	1	1	1
	11	0	1	0	0	1	0	0	0	0	0	0	0	0	0	1	1	0	1	1	1
	12	0	1	0	0	1	0	0	0	0	0	0	0	0	0	1	0	0	0	1	1
PNC	1	0	0	0	0	0	0	1	0	0	0	0	1	0	0	1	0	0	0	0	1
	2	0	0	0	0	1	0	0	0	0	0	1	1	0	0	0	0	1	1	0	1

Supplementary Table S1. Continued.

Site	Ind.	Locus																			
		221	222	223	224	225	226	227	228	229	230	231	232	233	234	235	236	237	238	239	240
CAÑ	6	1	1	0	0	0	0	0	1	0	1	0	0	0	1	0	0	1	1	1	1
	7	0	0	0	0	0	0	0	1	1	1	0	0	0	1	0	0	0	0	0	1
	8	0	0	0	0	0	0	0	0	0	1	0	0	0	0	0	1	0	0	0	0
	9	0	0	0	0	0	0	0	0	0	0	0	0	0	0	0	0	0	0	0	0
	10	0	0	0	0	0	0	0	0	0	0	0	0	0	0	0	1	0	0	0	0
	11	1	0	0	0	0	0	0	0	0	0	0	0	0	0	0	0	0	0	1	0
GIC	1	1	0	1	1	1	0	0	0	1	0	1	0	0	0	0	0	0	1	1	1
	2	0	0	1	0	0	0	0	0	1	0	0	0	1	0	0	0	0	0	0	0
	3	1	0	1	0	0	0	0	0	1	0	0	0	0	0	1	1	0	1	1	0
	4	0	0	1	1	0	1	1	1	1	1	1	0	0	0	0	0	1	1	0	0
	5	0	0	0	1	0	0	0	1	0	1	0	0	0	1	0	1	1	0	0	1
	6	0	0	0	0	0	0	0	1	0	1	0	0	0	1	0	0	0	0	0	1
ICM	1	1	1	1	0	0	0	0	1	1	1	0	0	0	1	0	0	1	1	0	1
	2	0	0	0	0	0	0	0	0	0	0	0	0	0	0	0	0	1	0	1	0
	3	0	1	0	0	1	0	0	1	0	0	0	0	0	1	0	1	0	0	1	1
	4	1	0	0	1	0	0	0	1	1	1	0	1	0	1	0	1	1	1	0	1
	5	1	0	0	1	0	0	0	1	1	0	0	0	1	0	0	0	1	1	1	0
	6	1	0	0	1	0	0	0	1	1	0	0	0	0	0	0	0	0	0	0	0
	7	0	0	0	0	0	0	0	0	0	0	1	0	0	0	0	0	0	0	0	0
	8	1	0	0	1	0	0	0	1	0	0	0	0	0	1	0	0	0	0	0	1
	9	1	0	0	0	0	0	0	0	0	0	0	0	0	0	0	0	0	0	0	0
	10	1	1	0	0	0	0	0	1	1	1	0	0	0	1	0	0	1	1	0	1
	11	1	1	0	0	0	0	0	0	1	1	0	0	0	1	0	1	0	0	0	1
	12	1	1	0	0	0	0	0	0	1	1	0	0	0	1	0	0	0	0	0	0
PNC	1	0	0	1	0	0	0	0	0	1	0	0	0	1	0	0	0	0	0	0	0
	2	1	0	1	0	0	0	0	0	1	0	0	0	0	0	1	1	0	1	1	0

Supplementary Table S1. Continued.

Site	Ind.	Locus 241	242	243	244	245	246	247	248	249	250	251	252	253	254	255	256	257	258	259	260
CAÑ	6	0	1	0	0	0	1	1	0	0	0	1	0	0	1	1	1	1	1	1	0
	7	0	1	0	0	0	1	1	0	0	0	1	0	0	1	1	1	0	1	1	0
	8	1	0	0	0	0	1	1	0	0	0	0	0	0	1	1	0	0	1	1	0
	9	0	1	0	0	0	1	1	0	0	0	0	0	0	1	1	0	0	1	1	0
	10	0	1	0	0	0	1	0	0	0	0	0	0	0	1	1	0	0	1	1	0
	11	0	0	0	0	0	1	1	0	0	0	0	0	0	1	0	0	0	1	1	0
GIC	1	1	1	0	1	1	0	1	1	1	0	1	1	0	1	1	0	0	0	1	1
	2	1	1	0	0	0	0	0	1	1	1	1	0	0	1	0	0	1	1	1	0
	3	1	0	0	1	0	0	1	1	0	1	1	1	0	1	0	0	0	1	1	0
	4	1	0	1	1	1	0	1	0	0	0	0	0	0	1	1	0	1	1	1	0
	5	0	0	1	1	0	0	1	0	1	1	1	0	0	1	1	0	0	1	0	0
	6	0	0	1	0	0	1	1	1	0	0	1	0	0	1	1	1	0	1	1	0
ICM	1	1	1	0	1	0	1	1	0	0	0	1	0	0	1	1	0	0	1	0	0
	2	0	0	0	0	0	0	1	0	0	1	1	0	0	1	1	0	0	1	1	0
	3	0	0	0	0	0	1	1	0	0	1	1	0	0	1	1	1	0	1	1	0
	4	1	1	0	1	1	1	1	0	1	1	1	0	0	1	1	1	0	1	1	0
	5	0	1	1	0	1	1	1	0	0	0	1	1	0	1	1	0	0	0	1	0
	6	0	1	1	0	0	0	1	0	0	0	1	0	0	1	1	0	1	1	1	0
	7	0	0	1	0	0	0	0	0	0	0	0	0	1	1	1	0	0	1	1	0
	8	0	1	0	0	0	1	1	0	0	0	1	0	0	1	1	1	0	1	1	0
	9	0	0	0	0	0	0	0	0	0	0	1	0	0	1	0	1	0	1	1	0
	10	1	1	1	0	1	0	1	0	1	1	1	0	0	1	1	1	0	1	1	0
	11	0	1	0	0	0	1	1	0	0	0	1	0	0	1	1	1	0	1	1	0
	12	0	0	0	0	0	1	1	0	0	0	1	0	0	1	1	1	0	1	1	0
PNC	1	1	1	0	0	0	0	0	1	1	1	1	0	0	1	0	0	1	1	1	0
	2	1	0	0	1	0	0	1	1	0	1	1	1	0	1	0	0	0	1	1	0

Supplementary Table S1. Continued.

Site	Ind.	Locus 261	262	263	264	265	266	267	268
CAÑ	6	1	0	0	1	0	0	0	1
	7	1	0	0	1	0	0	0	1
	8	1	0	0	0	0	0	0	0
	9	0	0	0	0	0	0	0	1
	10	1	0	0	0	0	0	0	1
	11	1	0	0	1	0	0	0	1
GIC	1	0	0	0	0	0	0	1	0
	2	0	1	1	0	1	0	0	0
	3	0	0	1	0	1	0	0	0
	4	1	0	0	0	0	1	1	1
	5	1	0	0	1	0	0	1	1
	6	1	0	0	1	0	0	0	1
ICM	1	1	0	0	1	0	0	0	1
	2	0	1	0	1	0	0	0	1
	3	0	0	0	1	0	0	1	1
	4	0	0	0	1	1	0	0	1
	5	1	0	0	0	0	0	0	1
	6	0	0	0	0	0	0	0	1
	7	0	0	0	0	0	0	0	0
	8	1	0	0	1	0	0	0	1
	9	1	0	0	0	0	0	0	0
	10	1	0	0	1	0	0	0	1
	11	1	0	0	1	0	0	0	1
	12	1	0	0	1	0	0	0	1
PNC	1	0	1	1	0	1	0	0	0
	2	0	0	1	0	1	0	0	0

Supplementary Table S1. Continued.

Site	Ind.	Locus																			
		1	2	3	4	5	6	7	8	9	10	11	22	13	14	15	16	17	18	19	20
	3	0	0	0	0	1	1	1	0	1	1	0	0	1	1	0	1	1	1	1	0
	4	0	0	0	0	0	0	0	0	0	0	0	0	0	0	0	0	0	0	0	0
	5	0	0	0	0	0	1	1	0	1	1	1	0	1	1	0	1	1	1	1	0
	6	0	0	0	0	1	1	1	0	0	1	1	0	1	1	0	0	1	1	1	0
	7	0	0	0	0	1	1	1	0	0	1	1	0	1	1	0	1	1	0	1	0
	8	0	0	0	0	0	1	1	0	0	1	1	0	1	1	0	1	1	1	1	0
	9	0	0	0	0	0	0	0	0	0	0	0	0	0	0	0	1	0	0	0	0
	10	0	0	0	0	1	1	1	0	1	1	1	0	1	1	0	1	0	1	1	0
	11	0	0	0	0	0	0	0	0	0	0	0	0	0	0	0	0	0	0	0	0
	12	0	0	0	0	1	1	1	0	0	1	0	0	1	1	0	1	1	0	1	0
	13	0	0	0	0	1	0	1	0	0	1	0	0	1	1	0	1	1	1	1	0
	14	0	0	0	0	0	1	1	0	1	1	1	0	0	1	0	1	1	0	0	0
PNC	15	0	0	0	0	0	0	0	0	0	0	0	0	0	0	0	0	0	0	0	0
	16	0	0	0	0	0	0	0	0	0	0	0	0	0	0	0	0	0	0	0	0
	17	0	0	0	0	1	1	1	0	1	1	1	0	1	1	0	1	1	1	1	0
	18	0	0	0	0	0	0	1	0	1	1	0	0	1	1	0	1	0	0	1	0
	19	0	0	0	0	1	0	1	0	1	1	1	0	1	1	0	1	1	1	1	0
	20	0	0	0	0	1	1	1	0	1	1	1	0	1	1	0	1	1	1	1	0
	21	0	0	0	0	0	1	1	0	1	1	1	0	1	1	0	1	1	1	1	0
	22	0	0	0	0	0	1	0	0	1	1	0	0	0	0	0	0	0	0	0	0
	23	0	0	0	0	0	0	1	0	0	1	0	0	1	1	0	1	1	1	1	0
	24	0	0	0	0	0	0	0	0	0	1	1	0	1	1	0	1	0	1	0	0
	25	0	0	0	0	0	0	1	0	1	1	1	0	1	1	0	1	1	1	1	0
	26	0	0	0	0	0	1	1	0	1	1	1	0	1	1	0	1	1	1	1	0
	27	0	0	0	0	1	1	1	0	0	1	0	0	1	1	0	1	1	1	1	0
	28	0	0	0	0	1	1	1	0	0	1	1	0	0	1	0	1	0	1	0	0

Supplementary Table S1. Continued.

Site	Ind.	Locus																			
		21	22	23	24	25	26	27	28	29	30	31	32	33	34	35	36	37	38	39	40
PNC	3	0	1	1	1	1	1	1	0	1	0	1	0	1	1	1	0	1	0	0	1
	4	0	0	0	0	0	0	0	0	0	0	1	0	0	0	1	0	0	0	0	0
	5	0	1	1	1	1	1	0	0	1	0	1	0	0	0	1	1	0	0	1	1
	6	1	1	1	1	1	1	0	0	1	0	1	0	1	0	1	1	0	0	0	0
	7	0	0	1	1	1	1	1	0	1	0	1	0	0	0	1	1	0	0	1	0
	8	1	1	1	1	0	1	1	0	1	0	1	0	0	1	1	1	1	0	0	1
	9	0	0	1	0	0	0	0	0	0	0	0	0	0	0	1	1	0	0	0	1
	10	0	0	1	1	0	1	1	0	1	0	1	0	0	1	1	1	1	0	1	1
	11	0	0	0	0	0	0	0	0	0	0	1	0	0	0	1	1	0	0	0	0
	12	1	0	1	1	1	1	0	0	1	0	1	0	0	0	1	0	1	0	1	1
	13	1	1	1	1	1	1	0	0	1	0	1	0	0	0	1	0	1	0	1	1
	14	0	1	1	1	1	1	1	0	1	0	1	0	0	0	1	1	1	0	1	0
	15	0	0	0	0	0	0	0	0	0	0	1	0	0	0	1	1	0	0	0	0
	16	0	0	0	0	0	0	0	0	0	0	1	0	0	0	1	1	0	0	0	0
	17	1	0	1	1	1	1	0	0	1	0	1	0	1	0	1	1	1	0	1	1
	18	1	0	1	0	0	1	0	0	0	0	1	0	0	0	1	1	1	0	1	0
	19	0	1	1	1	1	1	0	0	1	0	1	0	1	0	1	1	1	0	1	1
	20	1	1	1	1	0	1	0	0	1	0	1	0	0	0	1	0	1	0	0	1
	21	0	1	1	1	1	1	0	0	1	0	1	0	0	0	1	1	1	0	0	1
	22	0	0	1	1	1	1	0	0	0	0	0	0	1	0	1	1	0	0	0	0
	23	0	1	1	1	1	1	0	0	1	0	1	0	0	0	1	1	1	0	0	1
	24	0	0	0	0	1	1	0	0	0	0	1	0	0	0	1	1	0	0	0	0
	25	0	0	1	1	1	1	0	0	1	0	1	0	0	0	1	1	1	0	0	0
	26	0	1	1	0	1	1	0	0	1	0	1	0	0	1	1	0	1	0	0	1
	27	0	1	1	0	0	1	0	0	1	0	1	0	0	0	1	1	0	0	0	1
	28	0	1	1	0	1	1	0	0	1	0	1	0	0	0	1	1	1	0	1	1

Supplementary Table S1. Continued.

Site	Ind.	Locus																			
		41	42	43	44	45	46	47	48	49	50	51	52	53	54	55	56	57	58	59	60
PNC	3	1	1	1	1	1	0	1	0	0	1	0	1	0	1	1	1	0	1	1	0
	4	0	0	0	0	0	0	0	0	0	0	0	0	0	0	0	0	0	0	1	0
	5	1	1	1	1	1	0	1	0	0	1	0	0	0	1	1	1	0	1	1	0
	6	0	0	1	1	0	0	0	0	0	0	0	1	0	0	1	0	0	0	0	0
	7	1	1	1	1	1	0	1	0	0	0	0	1	0	1	1	0	0	1	1	0
	8	1	0	1	1	1	0	1	1	0	1	0	1	0	1	1	0	0	1	1	0
	9	0	1	1	0	0	0	0	0	0	0	0	0	0	0	0	0	0	0	0	0
	10	1	1	1	1	0	0	1	0	0	0	0	1	0	1	1	1	0	1	1	0
	11	0	0	0	0	0	0	0	0	0	0	0	0	0	0	0	0	0	0	1	0
	12	1	1	1	1	0	0	1	0	0	1	0	0	0	1	1	1	0	1	1	0
	13	1	1	1	1	0	0	1	0	0	1	0	1	0	1	1	1	0	1	1	0
	14	0	0	0	1	0	0	0	0	0	1	0	1	0	1	1	1	0	0	1	0
	15	0	0	0	0	0	0	0	0	0	0	0	0	0	0	0	0	0	0	0	0
	16	0	0	0	0	0	0	0	0	0	0	0	0	0	0	0	0	0	0	1	0
	17	1	0	1	1	1	0	1	1	0	0	0	0	0	1	1	1	0	1	1	0
	18	1	1	1	1	0	0	1	1	0	0	0	1	0	0	0	1	0	0	1	0
	19	1	1	1	1	1	0	1	0	0	0	0	1	0	1	1	1	0	1	1	0
	20	1	0	1	1	1	0	1	0	0	0	0	0	0	1	1	1	0	1	1	0
	21	1	1	1	1	1	0	1	0	0	0	0	0	0	1	1	1	0	1	1	0
	22	0	1	0	1	0	0	0	1	0	0	0	0	0	0	1	0	0	0	1	0
	23	1	1	1	1	1	0	1	0	0	1	0	0	0	1	1	1	0	1	1	0
	24	1	1	1	0	0	0	1	0	0	0	0	0	0	0	1	0	0	0	1	0
	25	1	1	1	1	0	0	1	0	0	1	0	0	0	1	1	1	0	1	1	0
	26	1	1	1	1	0	0	1	0	0	1	0	0	0	1	1	1	0	1	1	0
	27	1	1	1	1	0	0	1	0	0	0	0	0	0	1	1	1	0	1	1	0
	28	1	1	1	1	1	0	1	0	0	0	0	0	0	1	1	1	0	1	1	0

Supplementary Table S1. Continued.

Site	Ind.	Locus																			
		61	62	63	64	65	66	67	68	69	70	71	72	73	74	75	76	77	78	79	80
	3	1	0	0	0	1	0	0	0	0	1	0	0	1	1	0	1	0	1	1	1
	4	0	1	0	0	0	1	0	0	0	0	0	0	0	1	0	0	0	0	0	0
	5	0	0	0	0	0	0	0	0	1	0	0	0	1	1	1	1	0	1	1	0
	6	1	0	0	1	0	0	1	0	1	1	0	0	0	1	0	0	0	0	1	0
	7	0	1	0	1	0	0	0	0	1	0	0	0	0	1	1	1	0	1	1	1
	8	0	0	0	1	1	0	0	0	1	1	0	1	1	1	0	1	0	1	1	1
	9	0	0	0	1	1	1	0	0	0	0	0	0	0	1	0	0	0	0	0	0
	10	0	1	0	1	1	0	0	0	1	1	0	0	1	1	1	1	0	1	1	1
	11	0	1	0	1	1	1	0	0	0	0	0	0	0	1	0	0	0	0	0	0
	12	0	1	0	1	1	0	0	0	1	0	0	0	1	1	0	1	0	1	0	0
	13	0	1	0	1	1	1	0	0	1	0	0	0	1	1	0	1	0	1	1	0
	14	1	1	0	1	1	1	0	0	1	1	0	0	1	1	0	0	0	0	1	0
PNC	15	0	0	0	0	1	0	0	0	0	1	0	1	1	1	0	0	0	0	0	0
	16	0	0	0	1	0	0	0	0	0	0	0	0	0	1	0	0	0	0	0	0
	17	1	1	0	0	1	0	0	0	1	0	0	0	0	1	0	1	0	1	1	1
	18	1	1	0	0	1	0	0	0	1	1	0	1	1	1	0	0	0	0	0	0
	19	1	1	0	1	0	0	0	0	1	1	0	0	1	1	1	0	0	1	1	0
	20	1	1	0	0	0	1	0	0	1	0	0	0	0	1	1	1	0	1	1	0
	21	0	1	0	1	1	0	0	0	1	0	0	0	1	1	0	1	0	1	1	1
	22	1	1	0	1	1	1	0	0	0	0	0	0	0	1	0	0	0	0	0	0
	23	0	1	0	1	1	0	0	0	1	0	0	0	0	1	1	1	0	1	0	0
	24	0	1	0	1	1	0	0	0	1	0	0	0	0	0	0	0	0	0	0	0
	25	0	1	0	1	1	0	0	0	1	0	0	0	0	1	1	1	0	1	1	0
	26	0	1	0	1	1	0	0	0	1	0	0	0	0	1	1	1	0	1	1	0
	27	0	1	0	1	0	0	0	0	1	0	0	0	1	1	1	1	0	0	0	0
	28	0	1	0	1	0	0	0	0	1	0	0	0	0	1	1	1	0	1	1	0

Supplementary Table S1. Continued.

Site	Ind.	Locus																			
		81	82	83	84	85	86	87	88	89	90	91	92	93	94	95	96	97	98	99	100
PNC	3	1	1	1	1	1	1	1	1	0	1	1	0	1	1	1	1	1	1	1	1
	4	0	0	0	0	0	0	0	0	0	0	0	0	0	0	0	0	0	0	0	0
	5	1	1	0	1	0	1	1	1	1	1	1	0	1	1	1	1	1	1	1	1
	6	0	0	1	1	0	0	0	1	0	0	1	0	1	1	0	0	1	1	0	1
	7	1	1	1	1	1	1	1	0	0	0	1	0	1	1	1	0	1	0	1	1
	8	1	1	1	1	1	1	0	0	1	1	1	0	1	1	1	1	1	1	1	1
	9	0	0	0	0	0	1	0	0	0	0	0	0	0	0	0	0	0	0	0	1
	10	1	0	1	1	1	1	1	1	1	1	1	0	1	1	1	0	1	1	1	1
	11	0	0	0	0	0	0	0	0	0	0	0	0	0	0	0	0	1	0	0	0
	12	1	1	0	1	1	1	0	1	1	1	1	0	1	1	1	0	1	1	1	1
	13	1	1	0	1	0	0	1	1	1	1	1	0	0	1	1	1	1	0	1	1
	14	0	0	0	0	0	1	1	1	1	0	1	0	0	0	0	1	1	1	1	1
	15	0	0	0	0	0	0	0	1	0	0	0	0	0	0	0	0	1	0	0	0
	16	0	0	0	0	0	0	0	0	0	0	0	0	0	0	0	0	0	0	0	0
	17	1	1	1	1	1	1	1	1	0	0	1	0	1	1	1	1	1	1	1	1
	18	0	0	1	0	0	0	0	0	1	1	0	0	0	0	0	0	0	0	0	1
	19	0	1	1	1	1	1	1	0	0	1	1	0	1	1	1	1	1	0	0	1
	20	1	1	0	1	0	1	1	0	0	1	1	0	0	1	1	1	1	1	0	1
	21	1	1	0	1	1	1	0	0	0	1	1	0	0	1	1	1	1	0	0	1
	22	0	0	0	0	0	1	1	0	0	1	0	0	0	0	0	0	0	0	0	1
	23	1	1	1	1	1	1	1	0	0	0	1	0	0	1	1	1	1	0	0	1
	24	0	0	0	0	0	1	0	1	0	0	1	0	1	1	0	0	1	0	0	1
	25	1	1	0	1	1	1	1	0	0	0	1	0	1	1	1	1	1	0	0	1
	26	1	1	0	1	1	1	1	0	0	1	1	0	0	1	1	1	1	1	1	1
	27	1	1	0	1	1	1	1	0	0	0	1	0	0	1	1	1	1	0	1	1
	28	1	1	1	1	0	1	0	0	0	0	1	0	0	1	1	1	1	1	1	1

Supplementary Table S1. Continued.

Site	Ind.	Locus																			
		101	102	103	104	105	106	107	108	109	110	111	112	113	114	115	116	117	118	119	120
PNC	3	1	1	0	1	0	1	0	1	0	0	0	1	1	1	1	0	1	1	0	1
	4	0	1	0	0	0	0	0	0	0	0	0	1	0	0	0	0	0	0	0	0
	5	1	1	0	1	0	1	0	1	0	0	0	1	1	0	0	0	1	0	0	1
	6	1	1	0	1	1	0	0	0	0	0	0	0	0	1	1	0	0	0	1	1
	7	1	1	0	1	0	0	0	1	0	0	0	1	1	1	1	1	1	1	0	1
	8	0	1	0	1	0	1	0	1	0	0	0	1	1	1	1	0	1	0	1	1
	9	0	0	0	0	0	0	0	0	0	0	0	0	0	1	1	0	0	0	1	0
	10	1	1	0	1	0	1	1	1	0	0	0	1	1	1	1	0	0	1	1	1
	11	0	0	0	0	0	0	0	0	0	0	0	0	0	0	1	0	0	0	1	0
	12	1	1	0	1	0	1	0	1	0	0	0	1	1	1	1	0	1	1	1	1
	13	1	1	0	1	0	1	0	1	0	0	0	1	1	0	1	0	1	0	1	1
	14	1	1	0	1	1	1	1	1	0	0	0	1	1	1	1	1	0	1	0	1
	15	0	0	0	0	0	0	0	0	0	0	0	1	0	0	1	0	0	0	1	0
	16	0	0	0	0	0	0	0	0	0	0	0	1	0	0	0	0	0	0	0	0
	17	1	0	0	1	0	1	0	1	0	0	0	1	1	0	1	0	1	0	1	1
	18	0	0	0	0	0	0	0	1	0	0	0	1	0	0	1	0	0	0	0	0
	19	0	1	0	1	0	1	0	1	0	0	0	1	1	1	1	0	0	0	1	1
	20	0	1	0	1	0	1	0	1	0	0	0	0	1	0	0	0	0	0	1	1
	21	0	1	0	1	0	1	0	1	0	0	0	1	1	1	1	0	1	0	1	1
	22	0	0	0	0	0	0	0	0	0	0	0	1	1	1	1	0	0	0	1	0
	23	1	1	0	1	0	1	0	1	0	0	0	1	1	1	1	0	1	1	0	1
	24	0	0	0	1	0	0	0	0	0	0	0	0	1	0	1	0	0	0	0	0
	25	0	1	0	1	0	1	0	1	0	0	0	0	1	0	1	0	1	1	1	1
	26	0	1	0	1	0	1	0	1	0	0	0	0	1	0	1	0	1	0	1	1
	27	0	1	0	1	0	1	0	1	0	0	0	0	1	0	1	0	0	0	1	1
	28	0	1	0	1	0	1	0	1	0	0	0	0	1	0	1	0	1	1	0	1

Supplementary Table S1. Continued.

Site	Ind.	Locus																			
		121	122	123	124	125	126	127	128	129	130	131	132	133	134	135	136	137	138	139	140
	3	0	1	0	0	0	0	1	0	0	0	0	0	1	0	1	0	0	0	0	0
	4	0	0	0	0	0	0	0	0	0	0	0	0	0	0	1	0	0	0	0	0
	5	0	1	0	0	0	0	1	0	0	0	0	0	0	1	1	0	0	0	0	0
	6	0	1	0	1	0	0	0	0	0	0	0	0	1	0	1	0	0	0	0	0
	7	0	1	0	0	0	0	1	0	0	0	0	0	0	0	1	0	0	0	0	0
	8	0	1	0	0	0	0	1	0	0	0	0	0	0	1	1	0	0	0	0	0
	9	0	0	0	0	0	0	0	0	0	0	0	0	0	0	1	0	0	0	0	0
	10	0	1	0	0	0	1	1	0	0	0	0	0	0	1	1	0	0	0	0	0
	11	0	0	0	0	0	0	0	0	0	0	0	0	0	0	1	0	0	0	0	0
	12	0	1	0	0	0	0	1	0	0	0	0	0	0	1	1	0	0	0	1	0
	13	0	1	0	0	0	0	1	0	0	0	0	0	0	0	1	0	0	0	0	0
	14	0	1	1	0	0	0	1	0	0	0	0	0	0	1	1	0	0	0	0	0
PNC	15	0	0	0	0	0	0	0	0	0	0	0	0	0	0	1	0	0	0	0	0
	16	0	0	0	0	0	0	0	0	0	0	0	0	0	0	1	0	0	0	0	0
	17	0	1	0	0	0	0	1	0	0	0	0	0	0	0	1	0	0	0	0	1
	18	0	0	0	0	0	0	0	0	0	0	0	0	0	0	1	0	0	0	0	0
	19	0	1	0	0	0	0	0	0	0	0	0	0	0	0	1	0	0	0	0	0
	20	0	1	0	0	0	0	0	0	0	0	0	0	1	0	1	0	0	0	0	0
	21	0	1	0	0	0	0	0	0	0	0	0	0	0	0	1	0	0	0	0	0
	22	0	0	0	0	0	0	0	0	0	0	0	0	0	1	1	0	0	0	0	0
	23	0	1	0	0	0	0	0	0	0	0	0	0	0	0	1	0	0	0	0	1
	24	0	0	0	0	0	0	0	0	0	0	0	0	0	0	1	0	0	0	0	0
	25	0	1	0	0	0	0	0	0	0	0	0	0	0	0	1	0	0	0	0	0
	26	0	1	0	0	0	0	0	0	0	0	0	0	0	0	1	0	0	0	0	0
	27	0	1	0	0	0	0	0	0	0	0	0	0	0	0	1	0	0	0	0	0
	28	0	1	0	0	0	0	1	0	0	0	0	0	0	0	1	0	0	0	0	0

Supplementary Table S1. Continued.

Site	Ind.	Locus																			
		141	142	143	144	145	146	147	148	149	150	151	152	153	154	155	156	157	158	159	160
PNC	3	1	1	1	0	1	0	0	1	0	1	1	1	0	1	1	0	0	0	0	1
	4	0	0	0	0	0	0	0	1	1	1	1	1	0	1	1	1	0	1	1	1
	5	1	1	1	0	1	0	0	1	0	0	1	1	0	0	1	0	0	0	0	1
	6	0	0	0	0	1	0	0	0	0	1	1	1	0	0	1	1	0	0	0	0
	7	0	0	0	0	0	1	0	1	1	1	1	1	0	1	1	1	0	0	0	1
	8	1	1	1	0	1	0	0	1	0	1	1	0	0	0	0	1	1	0	0	0
	9	0	0	0	0	0	0	0	1	0	0	1	1	0	0	0	0	0	0	0	1
	10	1	1	0	0	1	0	0	1	0	1	1	1	1	0	1	1	1	0	0	1
	11	0	0	0	0	0	0	0	1	0	0	1	1	0	1	1	0	0	0	0	1
	12	1	1	0	0	1	0	0	1	1	1	1	1	1	1	1	1	0	0	0	1
	13	0	1	0	0	0	0	0	1	0	1	1	1	0	0	1	1	0	0	0	1
	14	0	0	0	0	1	1	0	0	0	1	1	1	1	0	1	1	0	0	0	0
	15	0	0	0	0	0	0	0	1	0	1	1	0	0	1	0	1	1	0	0	0
	16	0	0	0	0	0	0	0	0	0	0	1	1	0	0	0	0	0	1	0	1
	17	0	1	0	0	1	1	0	0	0	0	1	1	0	1	1	1	0	0	0	1
	18	1	0	0	0	0	0	0	0	1	1	1	0	1	1	1	1	0	0	0	0
	19	0	1	0	0	1	0	0	1	0	0	1	1	0	1	1	0	0	0	0	1
	20	1	1	1	0	1	0	0	1	0	1	1	1	0	1	1	1	0	0	0	0
	21	0	1	0	0	1	0	0	1	0	0	1	1	0	0	1	1	0	0	0	1
	22	0	0	0	0	0	0	0	0	0	0	1	1	0	0	1	1	1	0	0	1
	23	1	1	0	0	1	0	0	0	0	0	1	1	0	1	1	1	0	0	0	0
	24	0	0	0	0	0	0	0	0	0	0	1	1	0	0	1	0	0	0	0	0
	25	1	1	1	0	1	0	0	1	0	0	1	1	0	1	1	1	0	0	0	1
	26	1	1	1	0	1	0	0	1	0	0	1	1	0	0	1	1	0	0	0	0
	27	0	1	0	0	0	0	0	0	0	1	1	1	0	1	1	0	0	0	0	0
	28	1	1	0	0	1	0	0	0	0	1	1	1	0	1	1	1	0	0	1	1

Supplementary Table S1. Continued.

Site	Ind.	Locus																			
		161	162	163	164	165	166	167	168	169	170	171	172	173	174	175	176	177	178	179	180
	3	1	1	0	0	1	1	1	1	1	1	1	1	1	1	1	1	0	0	0	0
	4	1	1	0	0	1	1	1	0	1	1	0	1	0	1	1	1	0	0	0	0
	5	1	1	0	0	0	1	1	0	1	1	0	1	1	1	1	1	0	0	0	0
	6	0	1	0	1	1	1	0	0	1	1	1	1	1	0	1	1	0	0	1	1
	7	0	1	0	0	1	1	0	0	1	1	0	1	1	1	1	1	0	0	0	1
	8	1	1	0	0	1	1	1	1	1	1	1	1	0	1	1	1	0	0	0	0
	9	1	1	0	0	1	1	1	0	1	1	0	1	0	1	0	0	0	0	0	0
	10	1	1	1	1	0	1	1	0	1	1	1	1	1	1	1	1	0	1	0	0
	11	1	1	0	0	0	1	1	1	1	1	1	1	1	1	1	1	0	0	0	0
	12	0	1	1	0	1	1	1	1	1	1	0	1	1	1	1	0	0	0	0	0
	13	1	1	0	0	1	1	0	1	1	1	1	1	1	1	1	0	0	0	0	1
	14	1	1	0	1	1	1	0	0	0	1	1	0	0	0	0	0	0	0	0	0
PNC	15	1	1	0	0	1	1	1	1	1	1	1	1	1	1	1	1	0	0	0	1
	16	0	0	0	0	1	1	0	0	0	0	0	1	1	0	1	0	0	0	0	1
	17	1	1	0	0	1	1	1	1	1	0	0	1	1	1	1	0	0	0	0	0
	18	0	0	0	0	0	0	0	0	0	0	0	0	0	0	0	0	0	0	0	0
	19	1	1	0	0	1	1	1	1	1	1	0	1	1	1	1	0	0	0	0	0
	20	1	1	0	0	0	1	1	0	1	1	0	1	0	1	1	1	0	0	0	0
	21	1	1	0	0	0	1	1	0	1	1	0	1	1	1	1	0	0	0	0	0
	22	0	0	0	1	0	1	1	1	0	0	0	0	1	0	0	0	1	0	0	0
	23	1	1	0	1	0	1	1	0	0	0	0	1	0	1	1	0	0	0	0	0
	24	1	1	0	0	0	1	1	0	0	0	0	0	1	1	0	0	0	0	0	1
	25	1	1	0	0	0	1	1	1	0	1	0	1	0	1	1	1	0	0	0	0
	26	0	1	0	1	0	1	1	1	1	1	0	1	0	1	1	1	0	0	0	0
	27	1	1	0	0	0	1	1	0	1	1	1	1	1	1	1	1	0	0	0	0
	28	1	0	0	0	1	1	1	0	1	1	1	1	0	1	1	1	0	0	0	0

Supplementary Table S1. Continued.

Site	Ind.	Locus																			
		181	182	183	184	185	186	187	188	189	190	191	192	193	194	195	196	197	198	199	200
PNC	3	1	1	1	1	1	1	1	1	0	1	1	1	0	1	1	0	1	1	1	1
	4	1	1	1	1	1	0	1	1	1	1	1	1	0	1	1	0	1	0	1	1
	5	1	1	0	0	1	1	0	1	0	0	1	1	1	1	1	0	1	0	0	0
	6	0	1	1	1	1	1	0	0	0	1	0	0	0	0	1	1	1	0	1	1
	7	1	1	1	1	1	1	1	1	1	0	1	1	0	1	1	0	1	0	0	1
	8	1	1	1	0	1	1	1	1	1	1	1	1	0	1	1	0	1	1	1	1
	9	1	1	0	0	1	0	0	1	0	0	0	1	1	1	1	0	1	0	0	0
	10	1	1	1	1	1	0	1	1	1	1	1	1	0	1	1	0	1	1	0	1
	11	1	1	0	1	1	1	1	1	0	0	1	1	0	1	1	0	1	0	1	1
	12	1	1	1	0	1	1	1	1	1	1	1	1	0	1	1	0	1	1	0	1
	13	1	1	0	0	1	0	1	1	0	0	1	1	0	1	1	0	1	1	1	0
	14	1	1	1	0	1	1	1	1	0	0	0	0	0	1	1	0	1	0	0	0
	15	1	1	1	1	1	1	1	1	1	1	1	1	0	1	1	0	1	0	1	1
	16	1	1	0	1	1	1	0	0	0	0	1	0	0	1	0	0	0	0	0	0
	17	1	1	1	1	0	1	0	1	0	0	1	1	0	1	1	0	0	1	0	0
	18	0	1	1	1	0	0	0	0	0	0	0	0	0	0	0	0	0	1	0	0
	19	1	1	1	1	1	1	0	1	1	0	1	1	0	1	1	0	1	0	0	0
	20	1	1	0	0	1	0	1	1	1	1	1	0	1	1	1	0	1	1	0	1
	21	1	1	1	0	1	0	1	1	1	0	0	1	1	1	1	0	1	1	0	1
	22	0	1	1	0	0	0	0	1	0	1	0	1	1	1	0	0	0	0	0	0
	23	1	1	0	0	1	1	0	1	0	1	1	1	0	1	1	0	1	0	1	1
	24	1	1	0	0	0	0	0	1	0	1	0	0	0	0	0	0	1	0	0	0
	25	1	1	1	0	1	0	1	1	0	0	1	1	0	1	1	0	1	0	0	1
	26	1	1	1	0	1	1	1	1	1	0	1	1	1	1	1	0	1	1	1	1
	27	1	1	1	0	1	0	0	1	0	1	0	1	0	1	1	0	1	0	1	1
	28	1	1	1	0	1	0	0	1	1	0	1	1	1	1	1	0	1	0	1	0

Supplementary Table S1. Continued.

Site	Ind.	Locus																			
		201	202	203	204	205	206	207	208	209	210	211	212	213	214	215	216	217	218	219	220
	3	1	1	1	1	1	1	0	1	0	0	0	1	0	0	1	1	0	0	1	1
	4	1	1	0	1	1	0	0	1	0	0	1	1	0	0	1	1	0	1	1	1
	5	1	1	0	1	1	0	0	1	0	0	1	1	0	0	1	0	0	1	1	1
	6	0	1	0	1	0	1	1	1	0	0	1	0	0	0	1	0	1	1	0	1
	7	1	1	1	1	1	0	0	1	1	0	1	0	0	0	1	1	1	1	1	1
	8	1	1	0	1	1	0	1	1	0	0	1	0	0	0	0	1	0	0	1	1
	9	0	1	0	1	1	0	0	1	0	0	1	0	0	0	1	1	1	0	1	1
	10	1	1	0	0	1	0	0	1	0	0	1	1	0	1	1	0	0	1	1	1
	11	1	1	0	1	1	0	0	1	0	0	1	0	0	0	1	1	0	1	1	1
	12	1	1	1	1	1	0	0	1	0	0	1	0	0	0	1	0	0	1	1	1
	13	0	0	0	0	1	0	0	1	0	0	1	1	0	0	1	1	0	1	1	1
	14	0	1	0	0	0	0	0	0	1	1	1	0	0	0	1	1	0	1	0	1
PNC	15	1	1	1	1	1	0	1	1	0	0	1	1	0	0	1	1	0	1	1	1
	16	1	1	0	1	1	0	0	0	0	0	1	0	0	0	1	1	0	1	0	1
	17	1	1	0	1	1	0	0	1	0	0	1	0	1	0	1	1	0	1	1	1
	18	0	1	1	0	0	0	0	0	0	0	0	0	0	0	1	1	0	1	0	1
	19	1	1	0	1	1	0	0	1	0	0	0	1	0	0	1	1	1	0	0	1
	20	1	1	1	1	1	1	1	1	0	0	1	1	0	0	1	1	0	0	1	1
	21	1	1	0	1	1	0	0	1	0	0	1	1	0	0	1	1	1	1	1	1
	22	0	0	0	0	1	0	0	1	1	0	0	0	0	0	1	1	0	1	0	1
	23	1	1	0	1	1	0	0	1	0	0	0	0	0	0	1	1	0	0	1	1
	24	0	0	0	0	1	0	0	0	0	0	0	0	0	0	1	1	0	1	1	1
	25	1	1	1	1	1	1	0	1	0	0	1	0	0	0	1	1	1	0	1	1
	26	1	1	1	1	1	1	0	1	0	0	1	1	0	0	1	1	0	0	1	1
	27	0	1	0	1	1	0	1	1	0	0	1	0	0	0	1	1	0	0	1	1
	28	1	1	0	1	1	0	0	1	0	0	1	1	0	0	1	1	0	1	1	1

Supplementary Table S1. Continued.

Site	Ind.	Locus																			
		221	222	223	224	225	226	227	228	229	230	231	232	233	234	235	236	237	238	239	240
	3	1	1	1	1	1	0	0	1	1	1	0	0	1	1	0	1	1	1	0	1
	4	1	1	1	1	0	0	0	1	1	1	1	1	0	1	0	1	1	1	0	1
	5	1	1	0	1	1	0	0	1	1	1	0	0	0	1	0	1	1	0	0	1
	6	1	1	0	1	1	0	0	1	0	1	1	0	1	1	0	0	0	0	0	1
	7	1	1	0	1	0	0	1	1	1	1	0	0	1	1	0	1	1	1	1	1
	8	1	1	0	1	1	0	0	1	1	1	1	0	1	1	0	1	1	0	0	1
	9	1	1	0	0	0	0	0	1	1	1	0	0	0	1	0	1	1	0	1	1
	10	1	1	0	0	1	0	0	1	1	1	1	0	0	1	0	1	1	1	0	1
	11	1	1	1	1	0	0	0	1	1	1	0	0	1	1	0	1	1	1	0	1
	12	1	1	1	0	1	0	0	1	1	1	0	1	0	1	0	0	1	1	0	1
	13	1	1	0	0	1	0	0	1	1	1	0	0	1	1	0	1	1	0	0	1
	14	0	0	0	1	1	0	0	1	0	0	0	1	0	1	0	1	0	0	1	1
PNC	15	1	1	0	1	0	0	0	1	1	1	0	0	0	1	0	1	1	1	0	1
	16	1	1	0	1	0	0	0	0	1	1	0	0	0	0	0	0	1	1	1	1
	17	1	1	0	0	0	0	0	1	1	1	0	0	0	1	0	1	1	0	1	1
	18	0	0	0	0	0	0	0	0	0	0	0	0	0	0	0	1	0	1	1	0
	19	1	1	1	1	0	0	0	1	1	1	0	0	0	1	0	0	1	0	0	1
	20	1	1	1	1	0	0	0	1	1	1	1	0	0	1	0	0	1	1	0	1
	21	1	1	0	0	1	0	0	1	1	1	0	0	0	1	0	1	1	1	1	1
	22	1	1	1	1	1	0	0	0	0	1	0	0	0	0	0	1	1	1	0	1
	23	1	1	0	1	0	0	0	1	1	1	0	0	1	1	0	1	1	1	0	1
	24	1	0	0	0	0	0	0	0	1	0	0	0	0	0	0	0	0	1	0	0
	25	1	1	1	1	0	0	0	1	1	1	0	0	1	1	0	1	1	1	0	1
	26	1	1	1	1	1	0	0	1	1	1	0	0	0	1	0	1	1	1	0	1
	27	1	1	0	0	1	0	0	1	1	1	0	0	0	1	0	1	1	1	0	1
	28	1	1	1	0	1	0	0	1	1	1	0	0	1	1	0	1	1	1	0	1

Supplementary Table S1. Continued.

Site	Ind.	Locus																			
		241	242	243	244	245	246	247	248	249	250	251	252	253	254	255	256	257	258	259	260
PNC	3	1	1	0	0	1	1	1	1	1	0	1	0	1	1	1	1	0	1	1	0
	4	1	1	0	1	1	1	1	0	1	1	1	0	0	1	1	1	0	1	1	0
	5	0	1	1	0	1	1	1	0	1	1	1	0	0	1	1	0	0	1	1	0
	6	1	1	0	0	0	1	1	0	1	0	1	1	0	0	1	0	0	1	1	0
	7	0	1	0	1	1	1	1	0	1	1	1	0	1	1	1	1	1	1	1	1
	8	1	1	0	1	0	1	1	0	1	1	1	1	0	1	1	1	0	1	0	1
	9	0	0	0	0	0	1	1	0	0	0	1	0	0	1	1	0	0	1	1	0
	10	1	1	0	0	1	1	1	0	1	1	1	0	0	1	1	1	0	1	1	0
	11	1	1	0	0	1	0	1	1	1	0	1	0	0	1	1	0	0	1	0	0
	12	1	0	0	0	0	1	1	0	1	1	1	0	0	1	1	1	0	1	1	0
	13	1	0	0	0	0	1	1	0	1	0	1	0	0	0	1	1	0	1	1	0
	14	0	1	0	0	0	1	1	0	0	1	1	0	1	1	1	0	0	1	0	0
	15	0	1	1	0	1	1	1	0	1	1	1	0	1	1	1	1	0	1	1	0
	16	1	1	0	0	1	1	1	1	0	0	1	1	0	0	1	0	0	1	1	0
	17	0	1	0	0	1	1	1	0	1	0	1	0	0	1	1	0	0	1	1	0
	18	0	0	0	0	0	1	1	0	0	0	0	0	0	0	1	0	0	0	1	0
	19	1	1	1	0	0	1	1	0	0	0	1	0	0	1	1	1	0	1	1	0
	20	1	1	1	1	1	0	1	0	1	0	1	0	1	1	1	1	0	1	1	0
	21	0	1	1	1	1	1	1	0	1	1	1	0	0	1	1	1	0	1	0	0
	22	0	1	0	0	1	1	1	0	1	0	1	0	0	1	1	1	0	1	1	0
	23	1	1	0	0	1	1	1	0	1	0	1	0	0	1	1	1	0	1	1	0
	24	0	0	0	0	0	1	0	0	0	0	1	0	0	1	0	0	0	1	1	0
	25	0	1	1	0	1	1	1	0	1	0	1	0	0	1	1	0	0	1	1	0
	26	1	1	1	0	1	1	1	1	1	1	1	0	0	1	1	1	0	1	1	0
	27	1	1	0	0	1	1	1	0	1	1	1	0	0	1	1	1	0	1	1	0
	28	1	1	1	0	1	1	1	0	1	1	1	0	0	1	1	1	0	1	1	0

Supplementary Table S1. Continued.

Site	Ind.	Locus 261	262	263	264	265	266	267	268
PNC	3	1	0	0	1	0	0	1	1
	4	1	0	0	1	0	0	0	1
	5	1	0	0	1	0	0	0	1
	6	1	0	0	1	0	0	1	0
	7	0	0	0	1	0	0	0	1
	8	1	0	0	1	0	0	0	1
	9	1	0	0	1	0	0	0	0
	10	0	0	0	1	0	0	0	1
	11	1	0	0	1	0	0	0	1
	12	1	0	0	1	0	0	1	1
	13	1	0	0	1	0	0	0	1
	14	1	0	0	1	0	0	0	1
	15	1	0	0	1	0	0	0	1
	16	1	0	0	1	0	0	0	1
	17	1	0	0	1	0	0	0	1
	18	1	0	0	1	0	0	0	1
	19	1	0	0	1	0	0	0	0
	20	1	0	0	1	0	0	1	1
	21	1	0	0	1	0	0	1	1
	22	1	0	0	1	0	0	1	1
	23	1	0	0	1	0	0	1	1
	24	1	0	0	1	0	0	0	1
	25	1	0	0	1	0	0	1	1
	26	1	0	0	1	0	0	1	1
	27	0	0	0	1	0	0	1	1
	28	1	0	0	1	0	0	1	1

Supplementary Table S1. Continued.

Site	Ind.	Locus																			
		1	2	3	4	5	6	7	8	9	10	11	22	13	14	15	16	17	18	19	20
	29	0	0	0	0	0	1	1	0	0	1	1	0	0	1	0	1	0	1	0	0
	30	0	0	0	0	0	0	1	0	1	1	1	0	1	1	0	1	1	1	1	0
	31	0	0	0	0	0	1	1	0	1	1	1	0	1	1	0	1	1	1	0	0
PNC	32	0	0	0	0	0	0	0	0	1	1	1	0	1	1	0	1	1	1	0	0
	33	0	0	0	0	0	0	1	0	1	1	1	0	0	1	0	1	1	1	0	0
	34	0	0	0	0	0	0	1	0	0	1	1	0	1	0	0	1	1	0	0	0
	35	0	0	0	0	0	1	1	0	1	1	1	0	1	1	0	1	1	1	0	0
	36	0	0	0	0	0	1	1	0	0	1	1	0	1	1	0	1	1	1	1	0
	1	0	0	0	0	1	1	0	0	1	1	1	0	1	1	0	1	1	1	1	0
	2	0	0	0	0	0	1	0	0	1	1	1	0	0	1	0	0	0	1	1	0
	3	0	0	0	0	1	1	1	0	1	1	1	0	1	1	0	1	1	1	1	0
MAL	4	0	0	0	0	0	1	1	0	1	1	1	0	0	1	0	1	1	1	1	0
	5	0	0	0	0	0	1	1	0	1	1	1	0	1	1	0	1	1	1	1	0
	6	0	0	0	0	1	1	1	0	1	1	0	0	1	1	0	1	0	1	1	0
	7	0	0	0	0	1	1	1	0	1	1	1	0	1	1	0	0	0	1	1	0

Supplementary Table S1. Continued.

Site	Ind.	Locus																			
		21	22	23	24	25	26	27	28	29	30	31	32	33	34	35	36	37	38	39	40
PNC	29	0	1	1	0	1	1	0	0	1	0	1	0	0	0	0	1	1	0	0	1
	30	1	1	0	0	1	1	0	0	1	0	1	0	0	0	1	0	1	0	0	1
	31	0	1	1	0	1	1	0	0	1	0	1	0	0	0	0	1	1	0	0	1
	32	0	1	1	0	1	1	0	0	1	0	1	0	0	1	1	1	1	0	1	1
	33	1	1	1	1	1	1	0	0	1	0	1	0	0	0	1	1	1	0	0	1
	34	1	1	1	0	1	1	0	0	1	0	1	0	1	0	1	1	0	0	0	1
	35	1	1	1	1	1	1	0	0	1	0	1	0	0	0	1	1	1	0	0	1
	36	1	1	1	0	1	1	0	0	1	0	1	0	0	0	1	0	1	0	0	1
MAL	1	1	1	1	1	1	0	1	0	1	0	1	0	1	1	1	0	1	0	1	0
	2	1	0	0	1	1	1	1	0	1	0	1	0	1	0	0	1	1	0	0	1
	3	1	1	1	1	1	1	1	0	1	0	1	0	1	0	0	1	1	0	1	0
	4	1	0	1	0	1	0	1	0	1	0	1	0	1	0	0	0	1	0	1	0
	5	1	0	0	1	1	1	1	0	1	0	1	0	1	0	0	1	1	0	1	0
	6	1	1	0	0	1	0	0	0	1	0	1	0	1	0	1	1	1	0	0	0
	7	1	0	0	1	1	0	1	0	1	0	1	0	1	0	0	1	0	0	0	1

Supplementary Table S1. Continued.

Site	Ind.	Locus																			
		41	42	43	44	45	46	47	48	49	50	51	52	53	54	55	56	57	58	59	60
PNC	29	1	1	1	1	0	0	1	0	0	0	0	1	0	1	0	1	0	1	1	0
	30	1	1	1	1	1	0	1	0	0	0	0	0	0	0	1	1	0	1	1	0
	31	1	1	1	1	1	0	1	0	0	0	0	0	0	1	1	1	0	1	1	0
	32	0	1	1	1	1	0	1	0	0	1	0	0	0	1	1	1	0	1	1	0
	33	1	0	1	1	1	0	1	0	0	1	0	0	0	1	1	1	0	1	1	0
	34	0	0	1	0	0	0	0	0	0	0	0	1	0	0	0	1	0	1	0	0
	35	1	1	1	1	1	0	1	0	0	1	0	0	0	1	1	1	0	1	1	0
	36	1	1	1	1	0	0	1	0	0	0	0	0	0	1	1	1	0	1	1	0
MAL	1	1	1	1	1	1	0	1	1	0	1	0	1	0	1	0	1	0	1	1	0
	2	0	0	1	1	1	0	0	1	0	1	0	0	0	1	1	0	0	0	1	0
	3	1	1	1	1	1	0	0	1	0	1	0	1	0	1	1	1	0	1	1	0
	4	1	0	1	1	1	0	0	1	0	1	0	0	0	1	1	1	0	1	1	0
	5	0	1	1	1	1	0	0	0	0	1	0	1	0	1	0	1	0	1	1	0
	6	0	1	0	0	1	0	0	0	0	1	0	1	0	1	1	1	0	0	1	0
	7	1	1	1	0	1	0	0	1	0	1	0	0	0	0	1	1	0	1	1	0

Supplementary Table S1. Continued.

Site	Ind.	Locus 61	62	63	64	65	66	67	68	69	70	71	72	73	74	75	76	77	78	79	80
PNC	29	0	0	0	1	0	0	0	0	1	0	0	0	0	1	1	1	0	1	0	1
	30	0	0	0	1	1	0	0	0	1	0	0	0	0	1	0	1	0	0	1	0
	31	0	0	0	1	1	0	0	0	1	0	0	0	1	1	1	0	0	0	1	1
	32	0	1	0	1	1	1	0	0	1	0	0	0	0	1	1	1	0	0	0	0
	33	0	1	0	1	1	0	0	0	1	0	0	0	1	1	0	1	0	1	1	0
	34	0	1	0	1	1	1	0	0	1	0	0	0	0	1	0	0	0	1	0	1
	35	0	1	0	1	1	0	0	0	1	0	0	0	0	1	0	1	0	1	1	1
	36	0	0	0	1	1	0	0	0	1	0	0	0	0	1	0	1	0	1	1	1
MAL	1	1	1	0	1	1	1	1	0	1	1	0	1	1	1	1	1	0	1	0	1
	2	1	1	0	0	0	0	0	0	1	0	0	0	1	1	1	0	0	1	0	0
	3	1	1	0	0	1	1	0	0	1	1	0	1	1	1	1	0	0	0	1	0
	4	1	1	0	0	1	1	0	0	1	1	0	1	0	1	1	0	0	1	0	0
	5	1	1	0	1	1	1	0	0	1	1	0	0	0	1	1	1	0	1	1	0
	6	1	1	0	0	1	0	0	0	1	0	0	0	0	0	0	1	0	0	1	0
	7	0	1	0	0	1	1	0	0	1	0	0	1	1	1	1	1	0	1	1	0

Supplementary Table S1. Continued.

Site	Ind.	Locus																			
		81	82	83	84	85	86	87	88	89	90	91	92	93	94	95	96	97	98	99	100
	29	1	1	0	1	1	1	0	0	0	0	1	0	1	1	1	0	1	0	0	1
	30	0	1	0	0	0	1	0	0	0	0	1	0	0	1	1	1	1	1	0	1
	31	1	1	0	1	1	1	1	0	0	0	1	0	1	1	1	1	1	1	0	1
PNC	32	1	1	1	0	1	1	1	0	0	0	1	0	1	1	1	0	1	0	1	1
	33	1	1	0	1	0	1	1	0	0	0	1	0	0	1	0	0	1	1	0	1
	34	1	0	0	0	1	0	0	0	0	0	0	0	1	1	1	0	1	0	0	1
	35	1	0	1	1	0	1	0	0	0	1	1	0	1	1	1	1	1	0	0	1
	36	1	1	1	1	0	1	1	0	0	0	1	0	1	1	1	1	1	0	1	1
	1	1	0	1	1	1	0	0	0	1	1	1	0	1	0	1	1	1	1	1	1
	2	0	0	0	0	1	1	1	1	0	1	0	0	0	0	1	0	0	1	0	0
	3	0	0	1	0	1	1	1	0	1	1	1	0	1	0	1	1	1	1	1	1
MAL	4	0	0	1	0	1	0	1	1	0	0	0	0	1	0	1	1	1	1	1	1
	5	1	1	1	0	1	1	1	0	1	0	0	0	1	0	1	0	1	1	0	1
	6	1	0	1	0	1	1	0	1	1	1	0	0	0	0	0	0	1	1	0	1
	7	0	1	1	0	1	1	1	1	1	0	1	0	1	0	1	1	0	1	0	1

Supplementary Table S1. Continued.

Site	Ind.	Locus																			
		101	102	103	104	105	106	107	108	109	110	111	112	113	114	115	116	117	118	119	120
PNC	29	0	1	0	1	0	1	0	1	0	0	0	0	1	0	1	0	1	0	1	1
	30	0	1	0	1	0	1	0	1	0	0	0	1	1	0	1	0	1	0	1	1
	31	0	1	0	1	0	1	0	1	0	0	0	1	1	1	1	0	1	0	1	1
	32	1	0	0	1	0	1	0	1	0	0	0	1	1	0	1	0	1	0	1	1
	33	0	1	0	1	0	1	0	1	0	0	0	1	1	0	1	0	1	0	1	1
	34	0	1	0	1	0	1	0	1	0	0	0	0	0	0	0	0	1	0	1	1
	35	0	1	0	1	0	1	0	1	0	0	0	0	1	1	1	0	1	1	1	1
	36	0	1	0	1	0	1	0	1	0	0	0	0	1	0	1	0	0	1	1	1
MAL	1	1	1	0	1	1	1	1	1	0	0	0	1	1	1	0	1	0	1	1	0
	2	1	0	0	1	1	0	1	1	0	0	0	1	0	1	1	0	1	1	1	1
	3	1	1	0	1	1	0	1	0	0	0	0	1	1	1	0	1	0	1	1	0
	4	1	0	0	1	1	0	1	1	0	0	0	1	0	1	0	0	1	1	1	1
	5	1	0	0	1	1	0	1	1	0	0	0	1	1	1	1	0	0	0	0	0
	6	1	0	0	1	1	0	0	0	0	0	0	1	0	1	0	1	0	0	1	0
	7	1	0	0	1	1	0	1	0	0	0	0	0	0	1	0	0	1	0	1	0

Supplementary Table S1. Continued.

Site	Ind.	Locus																			
		121	122	123	124	125	126	127	128	129	130	131	132	133	134	135	136	137	138	139	140
PNC	29	0	1	0	0	0	0	0	0	0	0	0	0	0	0	1	0	0	0	0	1
	30	0	1	0	0	0	0	0	0	0	0	0	0	0	0	1	0	0	0	0	0
	31	0	1	0	0	0	1	0	0	0	0	0	0	0	0	1	0	0	0	0	1
	32	0	1	0	0	0	0	0	0	0	0	0	0	0	0	1	0	0	0	0	0
	33	0	1	0	0	0	0	0	0	0	0	0	0	0	0	1	0	0	0	0	0
	34	0	1	0	0	0	0	1	0	0	0	0	0	1	0	1	0	0	0	0	0
	35	0	1	0	0	0	0	0	0	0	0	0	0	0	0	1	0	0	0	0	0
	36	0	1	0	0	0	1	0	0	0	0	0	0	0	0	1	0	0	0	0	0
MAL	1	0	0	1	0	0	1	0	0	0	0	0	0	1	0	1	0	0	0	0	0
	2	0	1	1	0	0	0	1	0	0	0	0	0	1	0	1	0	0	0	0	0
	3	0	1	1	1	0	1	1	0	0	0	0	0	1	0	0	0	0	0	0	1
	4	0	1	1	0	0	1	1	0	0	0	0	0	1	0	0	0	0	0	0	1
	5	0	1	1	0	0	1	1	0	0	0	0	0	1	0	1	0	0	0	0	1
	6	0	0	1	0	0	1	1	0	0	0	1	0	0	0	1	0	0	0	1	0
	7	0	0	1	0	0	0	1	0	0	0	0	0	1	0	1	0	0	0	0	1

Supplementary Table S1. Continued.

Site	Ind.	Locus 141	142	143	144	145	146	147	148	149	150	151	152	153	154	155	156	157	158	159	160
PNC	29	1	1	0	0	1	0	0	0	0	0	1	1	1	1	1	0	0	0	0	1
	30	1	1	0	0	1	0	0	0	0	1	1	1	0	1	1	1	0	0	0	1
	31	1	1	0	0	1	0	0	0	0	1	1	1	0	0	1	1	0	0	1	1
	32	1	1	0	0	1	0	0	0	0	1	1	1	0	0	1	1	0	0	0	1
	33	1	1	0	0	1	0	0	0	0	1	1	1	1	1	1	1	0	0	1	1
	34	0	1	1	0	0	0	0	0	1	1	1	1	0	0	0	1	0	0	1	1
	35	1	1	0	0	1	0	1	0	0	1	1	1	0	0	1	1	0	0	1	0
	36	0	1	0	0	0	0	0	1	0	1	1	1	0	0	1	1	0	0	0	1
MAL	1	1	1	0	0	1	1	1	1	1	0	1	0	1	1	1	1	1	1	1	1
	2	1	1	0	0	1	0	1	0	1	0	0	1	1	1	1	0	1	1	0	1
	3	1	1	0	0	0	0	1	1	1	0	1	1	1	1	1	1	0	1	0	1
	4	1	1	1	0	1	0	1	0	1	0	1	1	1	1	0	0	0	1	1	0
	5	1	1	1	0	1	1	1	0	0	0	0	0	1	1	0	0	0	1	1	0
	6	0	0	0	0	0	0	1	1	0	0	0	0	0	1	1	0	0	1	1	0
	7	1	1	0	0	1	0	0	0	0	0	1	0	1	1	1	0	0	1	0	0

Supplementary Table S1. Continued.

Site	Ind.	Locus																			
		161	162	163	164	165	166	167	168	169	170	171	172	173	174	175	176	177	178	179	180
PNC	29	1	0	0	0	1	1	1	0	1	1	0	1	1	1	1	1	0	0	0	0
	30	1	0	0	0	1	1	1	1	0	0	0	1	0	1	1	1	0	0	0	0
	31	0	0	0	1	0	1	1	1	1	1	0	0	0	1	1	0	0	0	0	0
	32	1	1	0	0	1	0	1	1	1	0	0	0	1	0	1	1	0	0	0	0
	33	1	1	0	0	0	1	1	1	0	1	1	1	0	1	1	1	0	0	0	0
	34	1	0	1	0	1	1	1	0	1	0	0	0	1	1	1	1	0	0	0	0
	35	1	0	0	1	1	1	1	0	1	1	0	1	0	0	1	1	0	0	0	0
	36	0	0	0	0	0	1	1	1	1	0	1	1	1	0	1	0	0	0	0	0
MAL	1	1	0	1	1	1	1	0	0	1	1	1	1	0	0	1	1	1	1	1	0
	2	0	0	1	1	0	1	0	1	1	1	1	1	1	0	0	1	1	1	0	1
	3	0	0	1	1	1	0	0	1	0	1	1	1	1	0	1	1	1	1	1	1
	4	0	0	1	1	1	0	0	1	0	0	0	1	0	0	0	1	1	1	1	0
	5	0	0	1	1	1	0	1	1	0	1	1	1	1	0	0	1	0	1	0	1
	6	1	1	1	1	1	0	0	0	0	0	1	1	0	0	0	0	0	0	0	0
	7	0	0	1	1	1	0	0	1	1	1	0	0	0	0	0	1	1	1	1	0

Supplementary Table S1. Continued.

Site	Ind.	Locus																			
		181	182	183	184	185	186	187	188	189	190	191	192	193	194	195	196	197	198	199	200
PNC	29	1	1	0	0	1	1	1	1	0	1	1	1	0	1	1	0	1	1	1	1
	30	1	1	1	0	1	1	1	0	1	0	1	1	0	1	1	0	1	0	0	0
	31	1	1	0	1	1	0	1	1	0	0	1	1	0	1	1	0	1	0	1	0
	32	1	1	1	0	1	0	1	0	0	0	1	1	0	1	1	0	1	0	1	1
	33	0	1	1	0	1	0	0	1	1	0	1	1	1	1	1	0	1	1	0	0
	34	1	1	1	0	0	0	1	1	1	1	0	1	1	0	1	0	1	0	1	0
	35	1	1	0	0	0	0	1	1	1	0	1	1	1	1	1	0	1	0	0	1
	36	0	1	1	0	1	0	1	1	0	0	0	1	0	1	1	0	1	0	0	0
MAL	1	1	1	1	1	1	1	1	0	1	1	1	1	1	1	1	0	1	1	1	0
	2	1	1	0	1	1	1	0	0	1	1	1	0	1	1	0	0	1	1	1	0
	3	1	0	0	1	1	1	0	1	1	1	1	1	1	0	1	0	1	1	1	1
	4	1	1	0	1	1	0	0	0	0	1	1	1	1	1	0	0	1	1	1	0
	5	1	1	0	1	1	0	0	0	0	1	1	1	1	1	0	0	1	1	1	0
	6	0	0	1	1	1	0	0	0	0	1	1	1	0	0	1	1	1	0	0	0
	7	1	1	0	1	1	0	0	0	0	0	1	0	0	1	0	0	1	1	1	0

Supplementary Table S1. Continued.

Site	Ind.	Locus																			
		201	202	203	204	205	206	207	208	209	210	211	212	213	214	215	216	217	218	219	220
PNC	29	1	1	0	1	1	0	0	1	0	0	1	1	0	0	1	0	1	0	1	1
	30	0	1	1	1	1	1	1	1	0	0	1	1	0	0	0	1	0	1	1	1
	31	0	1	1	1	1	0	1	1	0	0	1	1	0	0	1	1	1	0	1	1
	32	1	1	1	1	1	1	1	1	0	0	1	1	0	0	1	1	1	0	1	1
	33	1	1	1	1	1	1	0	1	0	0	1	0	0	0	1	1	1	0	1	1
	34	1	1	1	1	1	0	0	0	1	1	0	1	0	0	1	1	1	1	1	1
	35	0	1	0	0	1	1	0	1	0	0	1	1	0	0	1	1	1	0	1	1
	36	1	1	1	1	1	1	0	1	0	0	1	1	0	0	0	1	1	0	1	1
MAL	1	0	1	1	1	1	1	1	1	0	0	1	1	1	1	0	1	1	1	1	1
	2	1	0	0	1	0	1	1	1	0	0	1	0	0	0	0	1	1	0	0	1
	3	1	0	1	1	1	0	1	1	1	0	1	1	0	0	0	1	1	1	1	1
	4	1	0	1	1	1	1	1	1	0	0	1	0	0	0	0	0	1	0	1	1
	5	1	0	0	1	1	0	1	1	0	0	1	0	0	0	0	1	1	1	1	1
	6	0	1	1	0	0	1	1	1	1	0	0	0	0	0	0	1	1	0	1	0
	7	1	0	1	1	1	1	1	1	0	0	1	0	0	0	0	1	0	0	1	1

Supplementary Table S1. Continued.

Site	Ind.	Locus																			
		221	222	223	224	225	226	227	228	229	230	231	232	233	234	235	236	237	238	239	240
PNC	29	1	1	0	0	1	0	0	1	1	1	0	1	0	1	0	1	1	1	1	1
	30	1	1	1	1	0	0	1	1	1	1	0	0	0	1	0	1	1	1	0	1
	31	1	1	1	1	1	0	0	1	1	1	0	0	0	1	0	1	1	1	0	1
	32	1	1	0	0	1	0	0	1	1	1	0	0	1	1	0	1	1	1	1	1
	33	1	1	1	1	0	0	0	1	1	1	0	0	0	1	0	1	1	1	0	1
	34	1	1	1	1	1	0	0	1	1	1	0	0	0	1	0	0	1	1	1	1
	35	1	1	0	1	0	0	1	1	1	1	0	1	0	1	0	1	1	1	1	1
	36	1	1	1	1	1	0	0	1	1	1	0	1	0	1	0	1	1	1	0	1
MAL	1	1	1	1	1	1	1	1	1	1	1	1	0	1	1	1	1	1	1	1	1
	2	1	0	1	1	1	0	0	0	1	1	1	0	1	0	1	1	1	1	1	0
	3	1	0	1	1	1	1	0	0	1	0	1	0	1	0	1	1	1	1	0	1
	4	1	1	1	1	1	0	0	0	1	1	1	0	1	0	1	0	1	1	1	0
	5	1	0	1	1	0	0	0	0	1	1	1	0	1	0	1	1	1	1	1	0
	6	1	1	1	1	0	0	0	0	1	0	1	0	0	0	0	1	0	1	1	1
	7	1	0	1	0	1	0	0	0	1	0	1	0	1	0	1	1	1	1	1	0

Supplementary Table S1. Continued.

Site	Ind.	Locus																			
		241	242	243	244	245	246	247	248	249	250	251	252	253	254	255	256	257	258	259	260
	29	1	1	1	0	1	1	1	0	1	1	1	0	0	1	1	1	0	1	0	0
	30	1	1	1	0	1	1	1	0	1	0	1	0	0	1	1	1	0	1	1	0
	31	1	1	1	0	1	0	1	0	1	1	1	0	0	1	1	1	0	1	0	0
PNC	32	0	1	1	0	1	1	1	1	1	0	1	0	0	1	1	1	0	1	0	0
	33	0	1	0	0	1	1	1	0	1	1	1	0	0	1	1	1	0	1	1	0
	34	0	0	1	0	0	1	1	0	1	0	1	0	0	1	1	1	0	1	1	0
	35	0	1	0	0	1	1	1	0	0	0	1	0	0	1	1	0	0	1	0	0
	36	0	1	1	0	1	1	1	0	1	0	1	0	0	1	1	1	0	1	1	0
	1	1	1	1	1	0	1	1	1	1	1	1	1	0	1	1	1	0	1	1	0
	2	1	1	1	0	1	0	1	1	1	1	1	1	0	1	0	1	1	1	1	0
	3	1	1	1	0	0	1	1	1	1	1	1	1	1	1	1	1	0	1	1	0
MAL	4	1	1	1	1	0	1	1	1	1	1	1	1	1	1	1	1	1	1	1	0
	5	1	0	1	0	0	0	1	1	0	1	1	1	0	1	1	1	0	1	1	0
	6	1	0	0	0	0	1	0	1	0	1	1	1	0	0	0	0	0	1	1	0
	7	1	0	0	0	1	0	1	1	1	1	1	1	0	1	0	1	0	0	1	0

Supplementary Table S1. Continued.

Site	Ind.	Locus 261	262	263	264	265	266	267	268
PNC	29	0	0	0	1	0	0	1	1
	30	0	0	0	1	0	0	1	1
	31	1	0	1	1	0	0	0	1
	32	0	0	1	1	0	0	1	1
	33	1	0	0	1	0	0	0	1
	34	0	0	0	1	0	0	0	1
	35	1	0	0	1	0	0	1	1
	36	1	0	0	1	0	0	1	1
MAL	1	0	1	1	1	1	0	1	1
	2	0	0	1	1	1	0	0	1
	3	0	0	1	1	1	0	1	1
	4	1	1	1	0	1	0	1	1
	5	1	0	1	1	1	0	1	1
	6	0	1	0	1	1	1	1	1
	7	1	1	1	0	1	0	1	1

Supplementary Table S2. List of outlier loci.

Locus	Obs. Het. BP	Obs FST	FST P-value	1-FST quantile	Distribution Loci
4	0.56969264	0.67291898	0.042079582	0.042079582	Andean, Coastal
7	0.47132271	0.57194206	0.030190228	0.030190228	Andean
10	0.49625813	0.57306712	0.024166991	0.024166991	Andean, Coastal
16	0.50105519	0.49842087	0.049859422	0.049859422	Andean, Coastal
23	0.48156732	0.58119943	0.024941667	0.024941667	Andean
24	0.50526435	0.031649442	0.034457564	0.96554244	Andean, Coastal
26	0.50401507	0.52380643	0.040858057	0.040858057	Andean, Coastal
**53	0.48413631	0.70372389	0.0069286275	0.0069286275	Coastal
60	0.43597392	0.69760909	0.012004477	0.012004477	Andean, Coastal
63	0.46476097	0.64687813	0.016880985	0.016880985	Coastal
65	0.47879369	0.0053174292	0.024843702	0.9751563	Andean, Coastal
67	0.24851132	0.56717052	0.033487611	0.033487611	Andean
73	0.50666375	0.022983405	0.027060457	0.97293954	Andean, Coastal
79	0.50717038	0.037211905	0.037453319	0.96254668	Andean, Coastal
80	0.46749604	0.031646497	0.049932277	0.95006772	Andean, Coastal
92	0.53563909	0.66618445	0.010705466	0.010705466	Andean, Coastal
**109	0.48463676	0.69603928	0.0076236336	0.0076236336	Coastal
123	0.3448807	0.6235612	0.012182591	0.012182591	Andean
125	0.49339302	0.59835824	0.018726882	0.018726882	Andean, Coastal
138	0.4633009	0.64927588	0.01683347	0.01683347	Coastal
142	0.43465004	0.60448925	0.027411497	0.027411497	Andean
169	0.49386659	0.0075410248	0.021721052	0.97827895	Andean, Coastal
173	0.50216667	0.0055660376	0.017444777	0.98255522	Andean, Coastal
**180	0.39814736	-0.020926197	0.006470165	0.99352984	Andean, Coastal
**181	0.36985597	-0.028845788	0.0043924445	0.99560756	Andean, Coastal
185	0.35608434	0.022387515	0.031533971	0.96846603	Andean, Coastal
186	0.45964013	-0.016012616	0.012265035	0.98773496	Andean, Coastal
**194	0.34502548	-0.020092187	0.0050921034	0.9949079	Andean, Coastal
209	0.16973637	0.0021624005	0.035922241	0.96407776	Andean, Coastal
210	0.094439772	-0.020700119	0.022334674	0.97766533	Andean, Coastal

218	0.49452551	-0.0032244613	0.0144847	0.9855153	Andean, Coastal
232	0.25490911	0.015574278	0.04041211	0.95958789	Andean, Coastal
239	0.48013413	0.026102411	0.043733828	0.95626617	Andean, Coastal
242	0.47509735	0.028424946	0.047547941	0.95245206	Andean, Coastal
253	0.23413018	0.0057245419	0.029274726	0.97072527	Andean, Coastal
**254	0.17995257	-0.028467252	0.0073131538	0.99268685	Andean, Coastal
256	0.4953038	0.032190277	0.041976162	0.95802384	Andean, Coastal
258	0.18421479	0.0065122371	0.037382024	0.96261798	Andean, Coastal
259	0.20736159	0.010663228	0.034606961	0.96539304	Andean, Coastal
260	0.08132797	-0.021459525	0.027598483	0.97240152	Andean, Coastal
261	0.44707364	0.013813467	0.030202974	0.96979703	Andean, Coastal

**Highly significant

Supplementary Figure S1. Detection of loci under selection from genome scans based on F_{ST}.

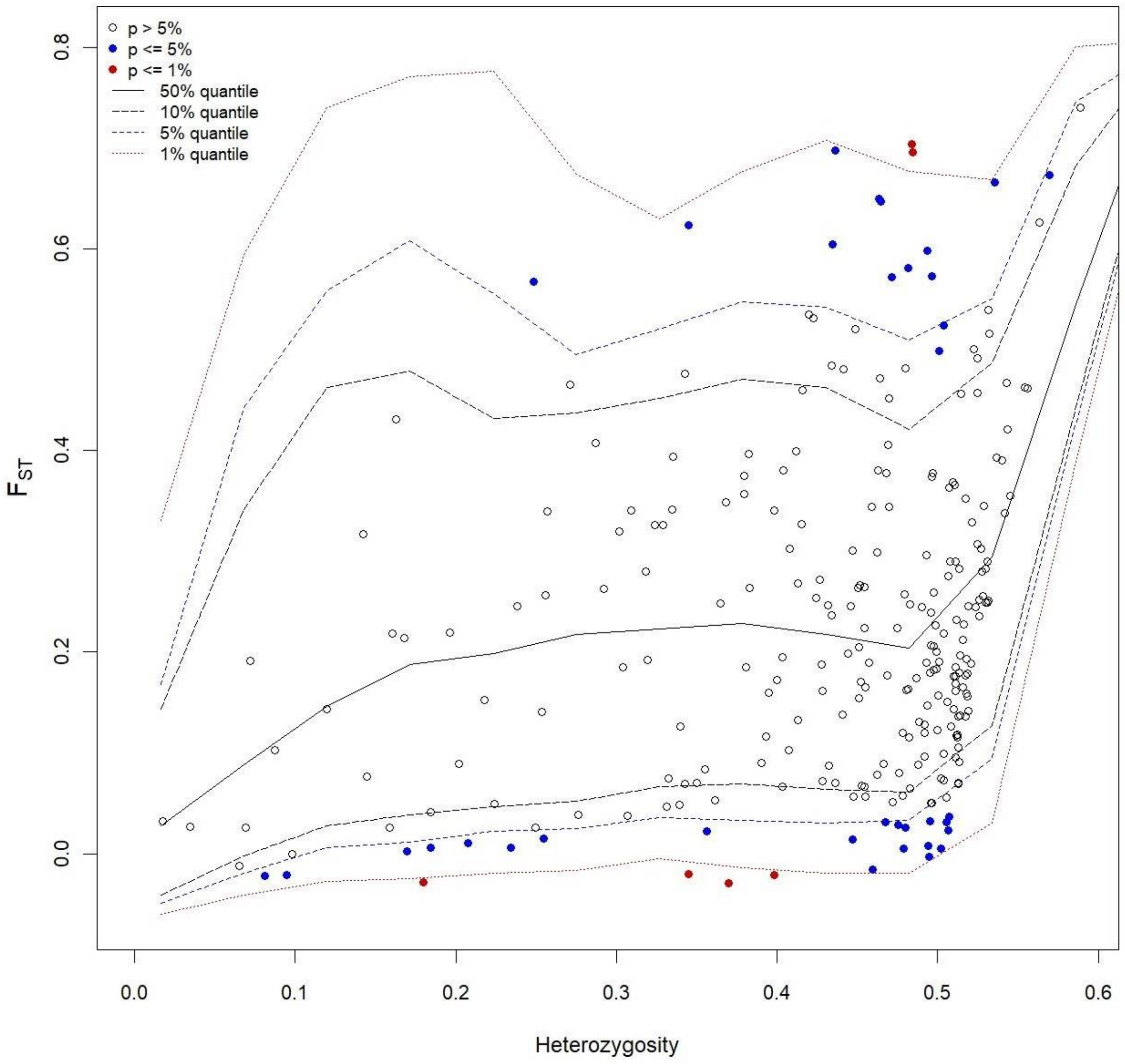

Supplementary Figure S2. Mismatch distribution (demographic expansion) for each studied site. POP1= PNN, POP2= VLA, POP3= CAÑ, POP4= GIC, POP5=ICM, POP6=PNC, POP7= MAL.

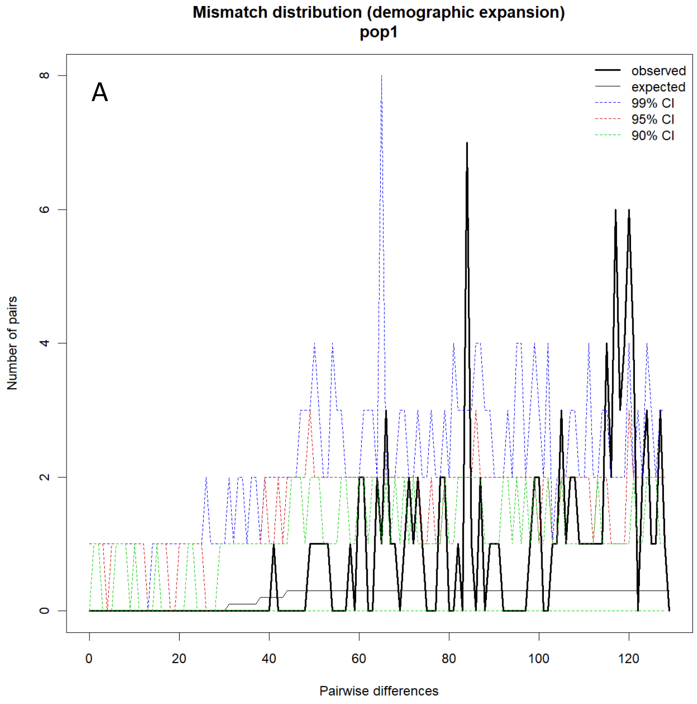

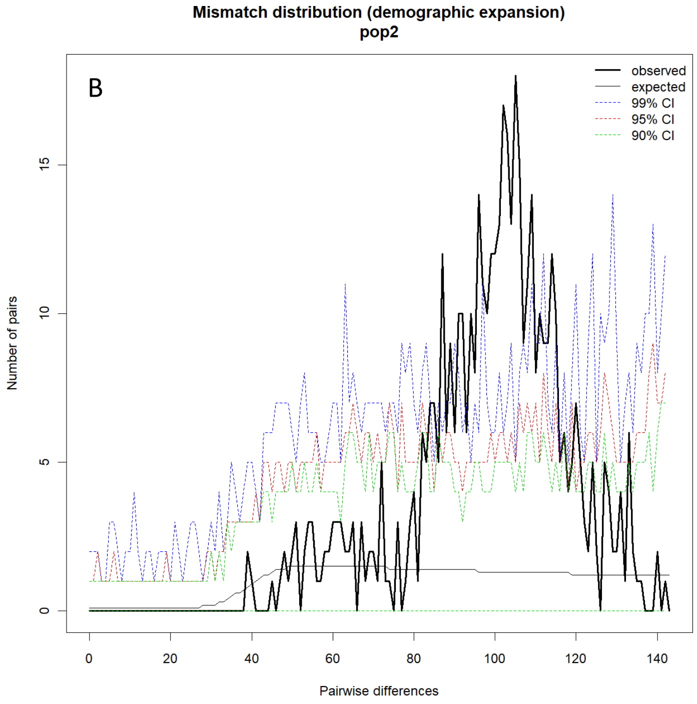

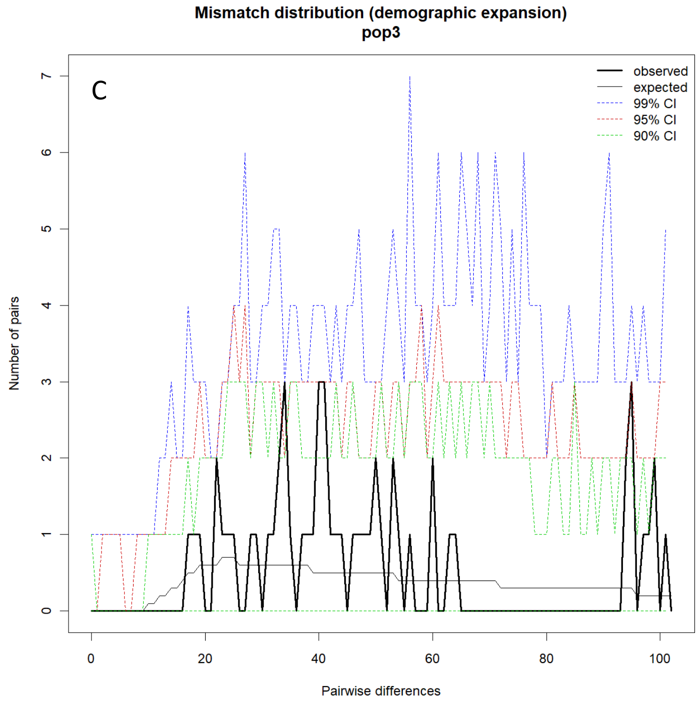
Mismatch distribution (demographic expansion)
pop3
C
observed
expected
99% CI
95% CI
90% CI
Number of pairs
Pairwise differences
0
1
2
3
4
5
6
7
0
20
40
60
80
100

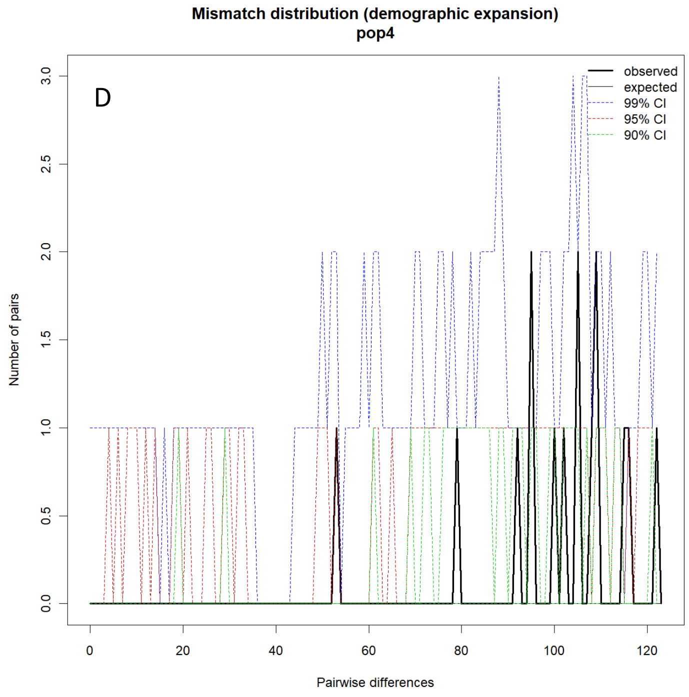
Mismatch distribution (demographic expansion)
pop4
D
observed
expected
99% CI
95% CI
90% CI
Number of pairs
Pairwise differences
0.0
0.5
1.0
1.5
2.0
2.5
3.0
0
20
40
60
80
100
120

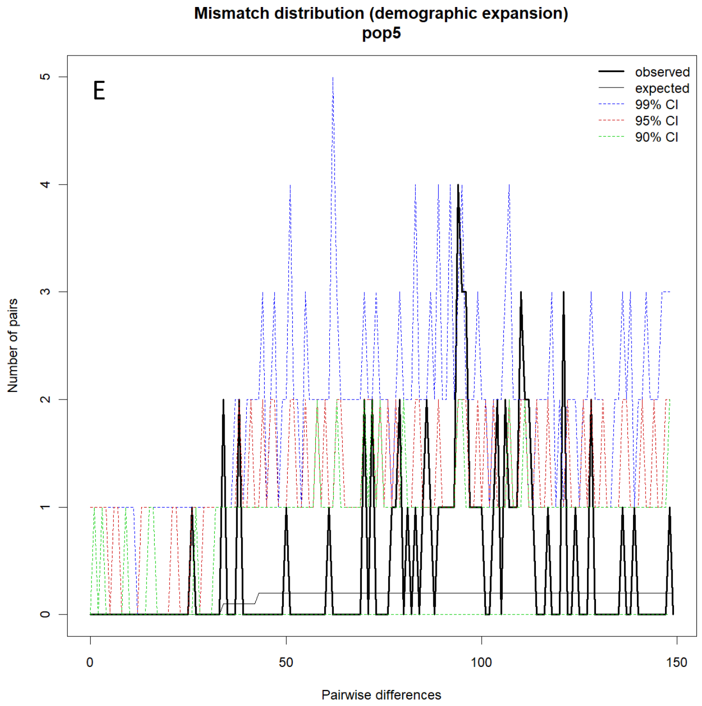

Mismatch distribution (demographic expansion)
pop5
E
observed
expected
99% CI
95% CI
90% CI
Number of pairs
0
1
2
3
4
5
0
50
100
150
Pairwise differences

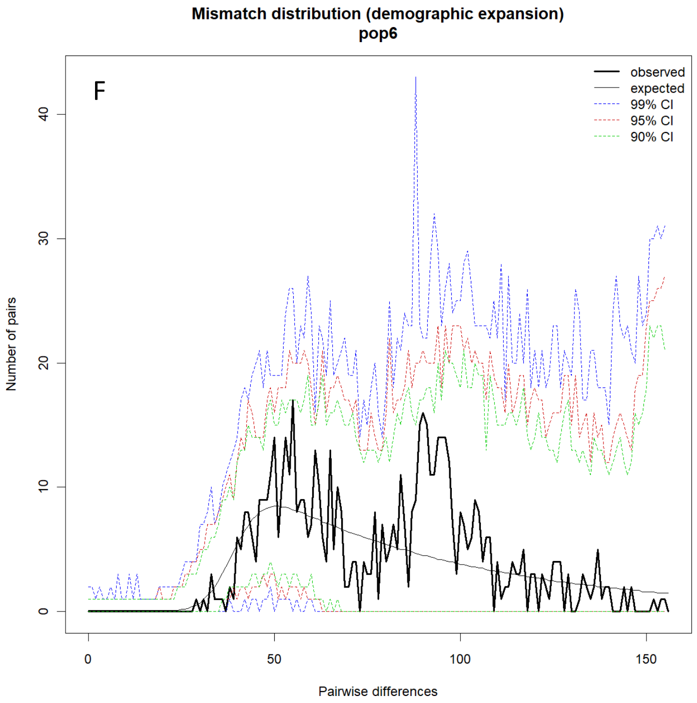

Mismatch distribution (demographic expansion)
pop6
F
observed
expected
99% CI
95% CI
90% CI
Number of pairs
0
10
20
30
40
0
50
100
150
Pairwise differences

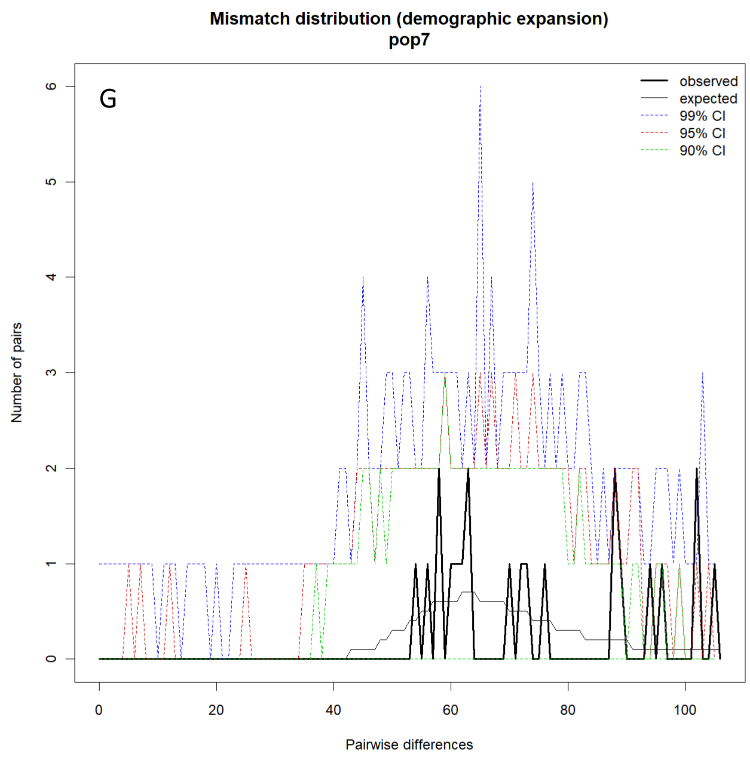

Mismatch distribution (demographic expansion)
pop7
G
observed
expected
99% CI
95% CI
90% CI
Number of pairs
0
1
2
3
4
5
6
0
20
40
60
80
100
Pairwise differences

Supplementary Table S3. Result of normality test Shapiro-Wilk (R project Package Rcmdr), for loci under selection and climatic variables. (+) positive result for normality test.

Outliers Loci	Normal	No- Normal
4	+	
7		+
10		+
16	+	
23	+	
24	+	
60	+	
63		+
65	+	
67	+	
73	+	
80	+	
92	+	
123		+
125		+
138		+
142	+	
169	+	
173	+	
185	+	
186	+	
209	+	
210	+	
218	+	
232	+	
239	+	
242	+	
253	+	
256	+	
258	+	
259	+	
260	+	
261	+	
194	+	
53		+
109		+
180	+	
181	+	
254	+	
ALT_W01	+	
BCLI15_W01	+	

BCLI18_W01	+	
BCLI02_W01	+	

Supplementary Figure S3. Barplot for different matrix.

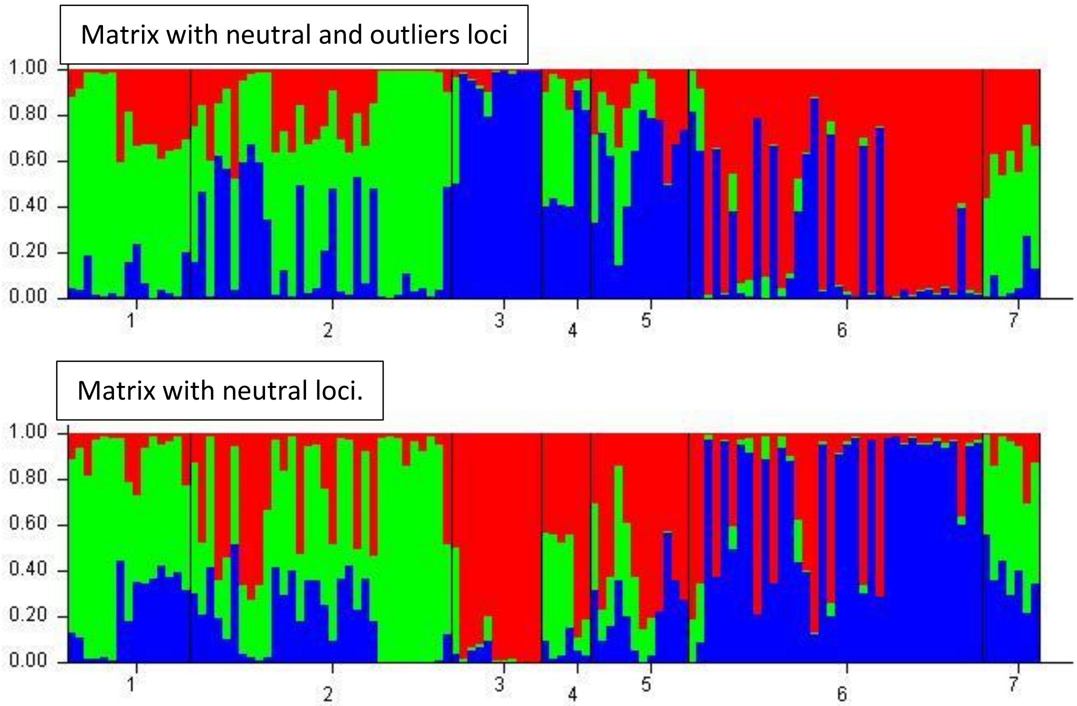

Supplementary Figure S4. Graphic of delta K for neutral matrix.

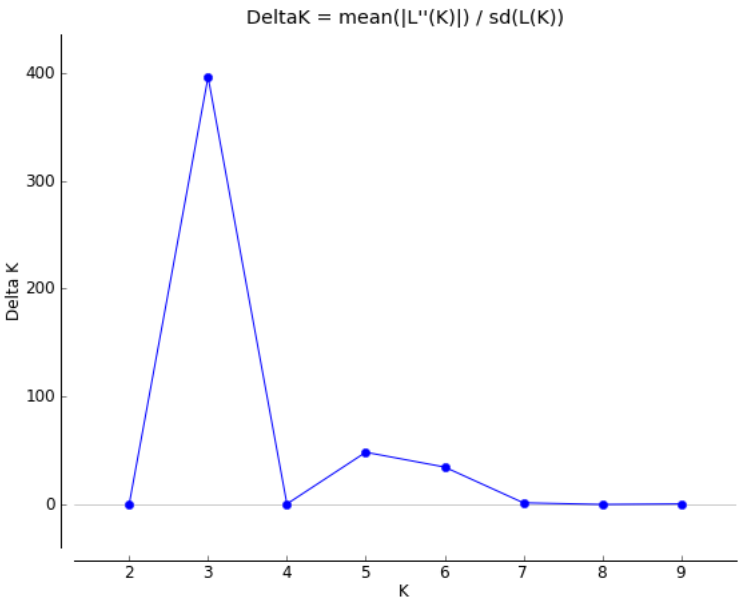

Supplementary Figure S5. Barplot for K=2, K=4, K=5, K=6, K=7 and K=8.

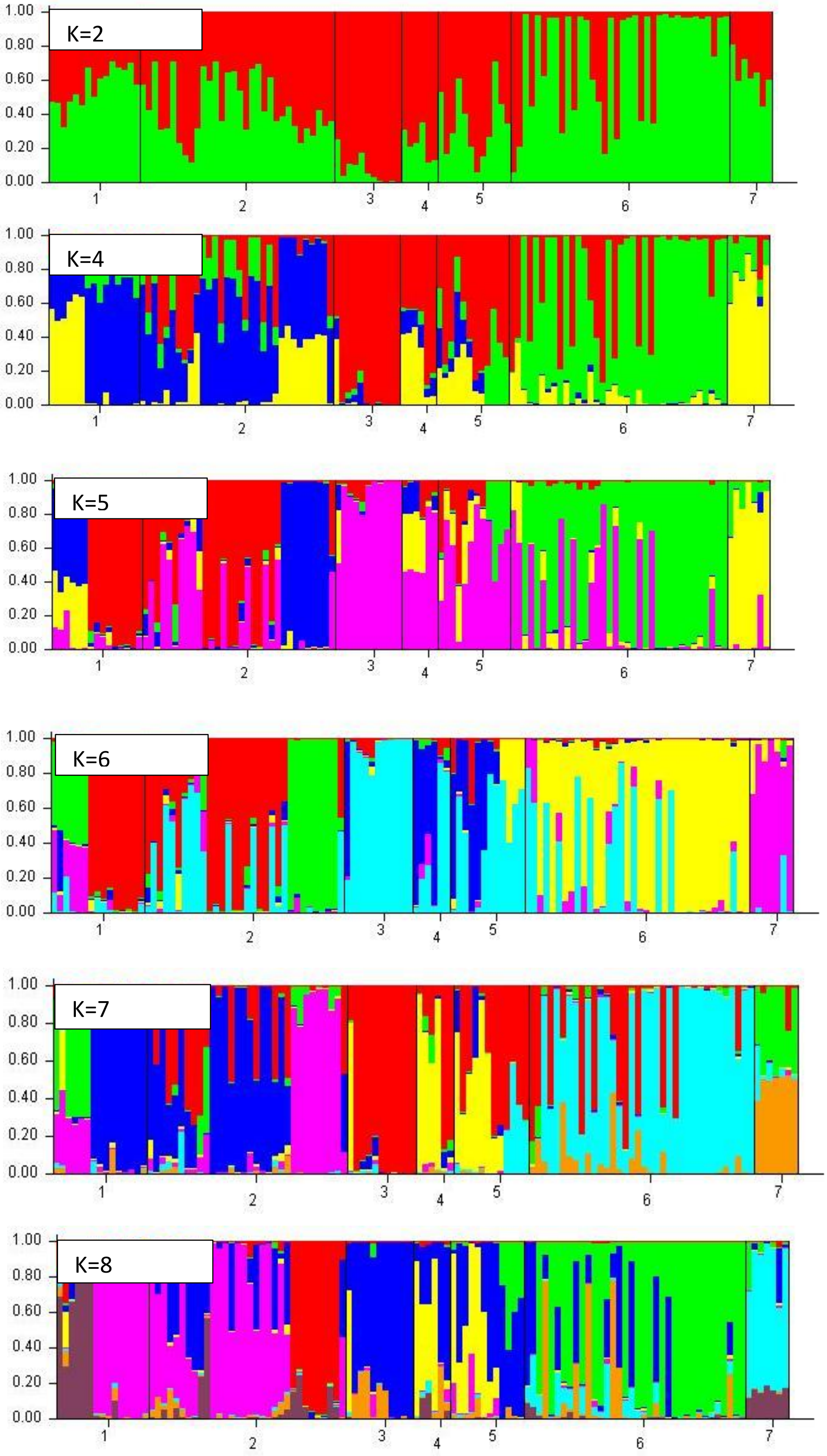

Supplementary Figure S6. Map of climatic variables and table of distribution in sites samples. Including Table with presence/absence of loci under selection. The maps were made in the program QGIS 3.16 Development Team
(2021, https://qgis.org/es/site/forusers/download.html). The climatic information was obtained of data base WorldClim (https://www.worldclim.org/data/bioclim.html) and map of Chile was obtained from the Ministerio del Medio Ambiente Gobierno de Chile (public information in https://www.ide.cl/)

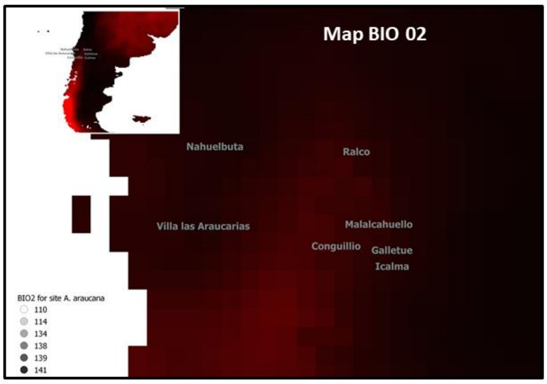

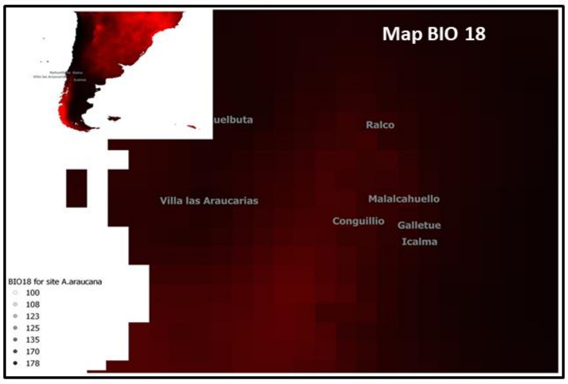

	4	7	10	16	23	26	53	60	63	67	92	109	123	125	138	142	209	210	232	253	260
PNN	1	0	1	1	0	1	1	1	1	0	1	1	0	1	1	0	1	1	1	1	1
VLA	1	0	1	1	0	1	1	1	1	0	1	1	0	1	1	0	1	1	1	1	1
CAÑ	1	0	0	0	1	0	0	1	0	1	0	0	1	0	0	0	0	0	0	0	0
GIC	1	0	0	0	1	0	0	1	0	1	1	0	1	0	0	0	0	0	0	0	1
ICM	1	0	1	1	1	1	0	1	0	1	1	0	1	1	0	1	1	0	1	1	0
PNC	0	1	1	1	1	1	0	0	0	1	0	0	1	0	0	1	1	1	1	1	1
MAL	0	1	1	1	1	1	0	0	0	1	0	0	1	0	0	1	1	0	0	1	0

Coastal sites
Andean Refugia
Andean recolonized areas

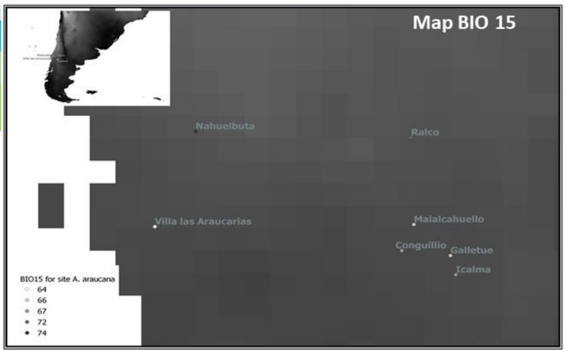

Maps of climatic variables and tables of presence/absence of loci under selection

Supplementary Figure S7. Graphic correlation of climatic variables BIO2 and loci outliers (26, 53, 259).

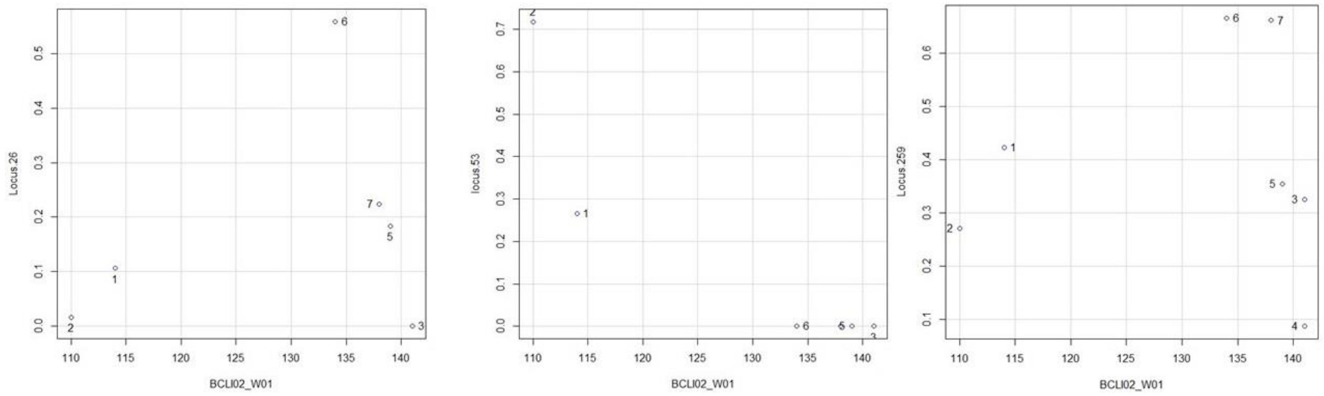

Supplementary Figure S8. Graphic correlation of climatic variables BIO18 and loci outliers (65, 65, 239).

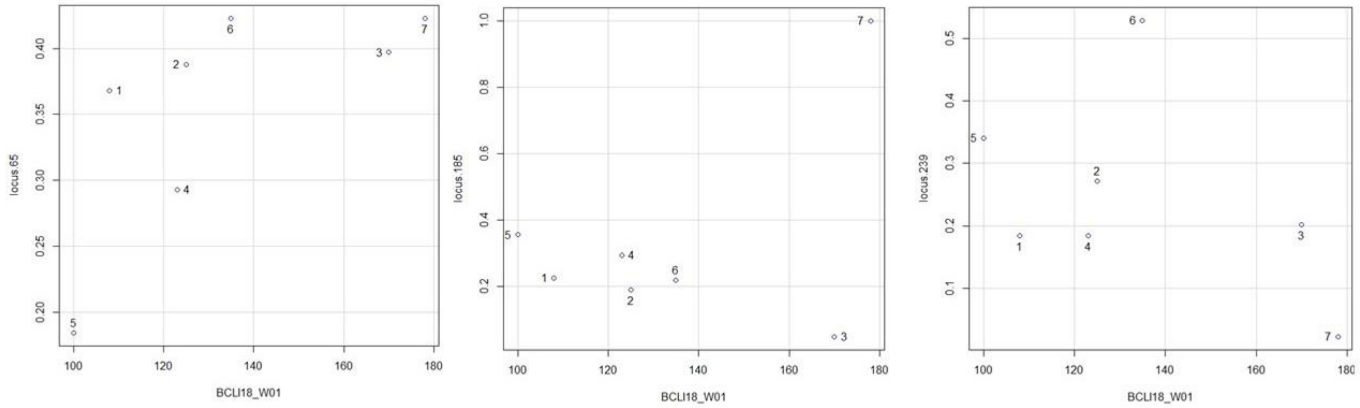

Supplement: Supplementary file 1 — Supplementary Information. [file 41598_2021_98662_MOESM1_ESM.pdf]
